# Assessing COVID-19 pandemic policies and behaviours and their economic and educational trade-offs across US states from Jan 1, 2020, to July 31, 2022: an observational analysis

**DOI:** 10.1016/S0140-6736(23)00461-0

**Published:** 2023-04-22

**Authors:** Thomas J Bollyky, Emma Castro, Aleksandr Y Aravkin, Kayleigh Bhangdia, Jeremy Dalos, Erin N Hulland, Samantha Kiernan, Amy Lastuka, Theresa A McHugh, Samuel M Ostroff, Peng Zheng, Hamza Tariq Chaudhry, Elle Ruggiero, Isabella Turilli, Christopher Adolph, Joanne O Amlag, Bree Bang-Jensen, Ryan M Barber, Austin Carter, Cassidy Chang, Rebecca M Cogen, James K Collins, Xiaochen Dai, William James Dangel, Carolyn Dapper, Amanda Deen, Alexandra Eastus, Megan Erickson, Tatiana Fedosseeva, Abraham D Flaxman, Nancy Fullman, John R Giles, Gaorui Guo, Simon I Hay, Jiawei He, Monika Helak, Bethany M Huntley, Vincent C Iannucci, Kasey E Kinzel, Kate E LeGrand, Beatrice Magistro, Ali H Mokdad, Hasan Nassereldine, Yaz Ozten, Maja Pasovic, David M Pigott, Robert C Reiner, Grace Reinke, Austin E Schumacher, Elizabeth Serieux, Emma E Spurlock, Christopher E Troeger, Anh Truc Vo, Theo Vos, Rebecca Walcott, Shafagh Yazdani, Christopher J L Murray, Joseph L Dieleman

**Affiliations:** a Council on Foreign Relations, Washington, DC, USA; b Institute for Health Metrics and Evaluation, University of Washington, Seattle, WA, USA; c Department of Applied Mathematics, University of Washington, Seattle, WA, USA; d Department of Health Metrics Sciences, School of Medicine, University of Washington, Seattle, WA, USA; e Department of Global Health, University of Washington, Seattle, WA, USA; f Henry M Jackson School of International Studies, University of Washington, Seattle, WA, USA; g Department of Political Science, University of Washington, Seattle, WA, USA; h Center for Statistics and the Social Sciences, University of Washington, Seattle, WA, USA; i Evans School of Public Policy & Governance, University of Washington, Seattle, WA, USA; j University of Pittsburgh, Pitssburgh, PA, USA; k Department of Public Policy, Harvard University, Cambridge, MA, USA; l Health Management and Policy, Drexel University, Philadelphia, PA, USA; m Seattle, WA, USA; n Munk School of Global Affairs and Public Policy, University of Toronto, Toronto, ON, Canada; o Department of Social and Behavioral Sciences, Yale University, New Haven, CT, USA; p Yale School of Public Health—Social and Behavioral Sciences, Yale University, New Haven, CT, USA

## Abstract

**Background:**

The USA struggled in responding to the COVID-19 pandemic, but not all states struggled equally. Identifying the factors associated with cross-state variation in infection and mortality rates could help to improve responses to this and future pandemics. We sought to answer five key policy-relevant questions regarding the following: 1) what roles social, economic, and racial inequities had in interstate variation in COVID-19 outcomes; 2) whether states with greater health-care and public health capacity had better outcomes; 3) how politics influenced the results; 4) whether states that imposed more policy mandates and sustained them longer had better outcomes; and 5) whether there were trade-offs between a state having fewer cumulative SARS-CoV-2 infections and total COVID-19 deaths and its economic and educational outcomes.

**Methods:**

Data disaggregated by US state were extracted from public databases, including COVID-19 infection and mortality estimates from the Institute for Health Metrics and Evaluation's (IHME) COVID-19 database; Bureau of Economic Analysis data on state gross domestic product (GDP); Federal Reserve economic data on employment rates; National Center for Education Statistics data on student standardised test scores; and US Census Bureau data on race and ethnicity by state. We standardised infection rates for population density and death rates for age and the prevalence of major comorbidities to facilitate comparison of states' successes in mitigating the effects of COVID-19. We regressed these health outcomes on prepandemic state characteristics (such as educational attainment and health spending per capita), policies adopted by states during the pandemic (such as mask mandates and business closures), and population-level behavioural responses (such as vaccine coverage and mobility). We explored potential mechanisms connecting state-level factors to individual-level behaviours using linear regression. We quantified reductions in state GDP, employment, and student test scores during the pandemic to identify policy and behavioural responses associated with these outcomes and to assess trade-offs between these outcomes and COVID-19 outcomes. Significance was defined as p<0·05.

**Findings:**

Standardised cumulative COVID-19 death rates for the period from Jan 1, 2020, to July 31, 2022 varied across the USA (national rate 372 deaths per 100 000 population [95% uncertainty interval [UI] 364–379]), with the lowest standardised rates in Hawaii (147 deaths per 100 000 [127–196]) and New Hampshire (215 per 100 000 [183–271]) and the highest in Arizona (581 per 100 000 [509–672]) and Washington, DC (526 per 100 000 [425–631]). A lower poverty rate, higher mean number of years of education, and a greater proportion of people expressing interpersonal trust were statistically associated with lower infection and death rates, and states where larger percentages of the population identify as Black (non-Hispanic) or Hispanic were associated with higher cumulative death rates. Access to quality health care (measured by the IHME's Healthcare Access and Quality Index) was associated with fewer total COVID-19 deaths and SARS-CoV-2 infections, but higher public health spending and more public health personnel per capita were not, at the state level. The political affiliation of the state governor was not associated with lower SARS-CoV-2 infection or COVID-19 death rates, but worse COVID-19 outcomes were associated with the proportion of a state's voters who voted for the 2020 Republican presidential candidate. State governments' uses of protective mandates were associated with lower infection rates, as were mask use, lower mobility, and higher vaccination rate, while vaccination rates were associated with lower death rates. State GDP and student reading test scores were not associated with state COVD-19 policy responses, infection rates, or death rates. Employment, however, had a statistically significant relationship with restaurant closures and greater infections and deaths: on average, 1574 (95% UI 884–7107) additional infections per 10 000 population were associated in states with a one percentage point increase in employment rate. Several policy mandates and protective behaviours were associated with lower fourth-grade mathematics test scores, but our study results did not find a link to state-level estimates of school closures.

**Interpretation:**

COVID-19 magnified the polarisation and persistent social, economic, and racial inequities that already existed across US society, but the next pandemic threat need not do the same. US states that mitigated those structural inequalities, deployed science-based interventions such as vaccination and targeted vaccine mandates, and promoted their adoption across society were able to match the best-performing nations in minimising COVID-19 death rates. These findings could contribute to the design and targeting of clinical and policy interventions to facilitate better health outcomes in future crises.

**Funding:**

Bill & Melinda Gates Foundation, J Stanton, T Gillespie, J and E Nordstrom, and Bloomberg Philanthropies.

## Introduction

In the words of one commentator, COVID-19 “defeated America”; it “humbled and humiliated the planet's most powerful nation”.^1^ The country's struggles have persisted throughout the pandemic and were not limited to a single time period or political leader. The USA has reported some of the highest COVID-19 death rates and confirmed SARS-CoV-2 infection rates in each year of the pandemic. Although the USA played a leading role in developing effective vaccines against COVID-19 and had an ample and early supply of doses, it nevertheless ranks 66th among countries and territories globally in terms of the proportion of residents who have completed their initial vaccine sequence.^2^ The USA has performed poorly, despite having (by most measures) the largest economy, spending the most on health care,^3^ and, before the emergence of SARS-CoV-2, having been rated the best prepared for a pandemic out of 195 nations considered.^3^, ^4^ Yet, while the USA has struggled against COVID-19, not all states struggled equally. Vermont (111 reported deaths per 100 000 people), Utah (157 reported deaths per 100 000), and Washington (193 reported deaths per 100 000) have reported COVID-19 death rates similar to those in Denmark (115 reported deaths per 100 000), Switzerland (155 reported deaths per 100 000), and Germany (170 reported deaths per 100 000) as of July 31, 2022. By contrast, the reported death rates in Mississippi (551 deaths per 100 000 people), Arizona (539 deaths per 100 000), and West Virginia (575 deaths per 100 000) are roughly three times higher and similar to the three nations with the highest death rates in the world during the same period—Russia (537 deaths per 100 000), Bulgaria (539 deaths per 100 000), and Peru (631 deaths per 100 000).

Several studies have sought to answer discrete policy questions concerning the differences in performance between individual US states during COVID-19, but the driving factors behind cross-state variation in SARS-CoV-2 infections and COVID-19 mortality remain poorly understood. For example, research suggests that state policy mandates on physical distancing,^5^, ^6^ mask use,^7^, ^8^ and vaccination have promoted positive health outcomes,^9^, ^10^ whereas income inequality, race, and poverty are linked to higher rates of SARS-CoV-2 infections and COVID-19 deaths,^11^, ^12^, ^13^ with partisan politics influencing mandate implementation and adherence at the state level.^14^, ^15^, ^16^, ^17^, ^18^ However, these studies have been limited in several important ways. First, many did not adjust for factors outside of policy makers' immediate control (eg, age or population density) that increase the risk of death after infection. Second, they often assessed only a subset of states or a short period. Third, they have used less reliable measures of infections and deaths that do not account for incomplete reporting. Furthermore, few studies have assessed the debate over whether better COVID-19 outcomes offset the economic, educational, and employment losses that are possibly associated with non-pharmaceutical interventions. A working paper considers the economic and educational trade-offs for improved COVID-19 health outcomes but aggregates measures in a way that prevents policy-relevant focused analysis, does not consider infections, and does not examine the drivers of interstate differences in outcomes.^19^

Identifying the factors associated with US cross-state variation in infection and mortality rates could help to answer fundamental policy questions that have emerged over the course of this pandemic and offer clues on how to better respond to this and future pandemic threats. Our results are broadly relevant, as there are other countries besides the USA that are democracies that are increasingly polarised,^20^, ^21^, ^22^ exhibit income and racial inequalities, have federal political systems,^23^, ^24^, ^25^ and have historically low levels of public trust.^26^ Notably, US politicians were not the only ones to argue that the “cure can't be worse than the disease”^27^ when opposing public health policies perceived to override individual rights and economic needs.^28^

We sought to complete an exploratory analysis of factors potentially associated with COVID-19 prevention and treatment across 50 US states and Washington, DC, between Jan 1, 2020, and July 31, 2022. We used that analysis to answer five key policy questions: 1) what role did social, racial, and economic inequities have in interstate variation in COVID-19 outcomes; 2) did states with greater health-care and public health capacity perform better; 3) how did politics influence the results; 4) did the states that imposed more policy mandates and sustained them longer do better; and 5) were there trade-offs between a state having fewer cumulative SARS-CoV-2 infections and total COVID-19 deaths and its economic and educational outcomes?

**Research in context**

**Evidence before this study**

The USA has captured researchers' attention for its suboptimal response to the COVID-19 pandemic. Despite having ranked the highest in pandemic preparedness out of 195 countries in the Global Health Security Index and scoring very well in WHO's Joint External Evaluation, the USA maintains the highest number of recorded COVID-19 deaths and has one of the highest per capita fatality rates from COVID-19 globally. However, the pandemic did not affect all US states equally. Between June 1, 2022 and Feb 14, 2023, we searched for published articles in Web of Science, Scopus, and PubMed using the terms “state,” “county,” “COVID-19,” and “United States”. We identified several studies that have sought to answer policy questions to help explain differences in individual states' performance during the COVID-19 pandemic, but a broad assessment of these questions and the mechanisms behind cross-state variation in SARS-CoV-2 infections and COVID-19 mortality has yet to be done. Some studies have highlighted the role of partisanship in the adoption of responsive policies such as physical distancing, mask use, and vaccine mandates, while others have examined links between racial, social, and economic inequities and state differences in COVID-19 outcomes. Many of these studies assess only a subset of US states or brief periods, do not adjust for factors outside of policy makers' immediate control (eg, age or population density), and fall short of explaining whether better COVID-19-related outcomes offset the economic, educational, and employment losses associated with these mandates.

**Added value of this study**

We analysed government policy responses and population behaviours in each US state to assess their associations with cumulative SARS-CoV-2 infections, total COVID-19 deaths, and economic and educational performance measures. We controlled for factors with known direct and biological connection to SARS-CoV-2 infection and COVID-19 death rates: age patterns, population density, and comorbidities such as obesity and diabetes. This study adds to the existing literature by conducting these analyses on all 50 US states and Washington, DC, and over a long study period—Jan 1, 2020, to July 31, 2022—to answer fundamental policy questions about US interstate variation in COVID-19 outcomes. The cross-state differences in COVID-19 infection and mortality rates suggest that the USA had the capacity to perform better than observed. There were a number of key policy-relevant findings. First, a subset of social and economic inequities were statistically associated with more SARS-CoV-2 infections and worse COVID-19 mortality rates, most notably poverty, lower educational attainment, higher rates of key comorbidities, limited access to quality health-care services, and lower interpersonal trust. This cluster of traits exists in states with large racial disparities and where most voters voted for the 2020 Republican presidential candidate. Second, access to quality health care was associated, on average, with fewer total COVID-19 deaths and SARS-CoV-2 infections, but higher public health spending and more public health personnel per capita were not associated with infection or death rates, at least not at the state level. Third, there was no association between the political affiliation of the state governor and lower SARS-CoV-2 infections or COVID-19 deaths, but there was an association between worse COVID-19 outcomes and the fraction of a state's voters who voted for the 2020 Republican presidential candidate. Fourth, our results suggest that vaccine coverage is linked to fewer COVID-19 deaths, and protective mandates and behaviours were associated with fewer infections. Fifth, we found no evidence of a choice between a state having a relatively strong economy or better COVID-19 health outcomes, but there were trade-offs with better employment rates. Moreover, several policy mandates were associated with lower fourth-grade mathematics test scores.

**Implications of all the available evidence**

Our analysis yields important insights for policy makers seeking to construct a more resilient and realistic response to future pandemic threats. COVID-19 magnified the polarisation and persistent social, economic, and racial inequities that already existed across US society, but the next pandemic threat need not do the same. Recognising the local contexts in which SARS-CoV-2 infections and COVID-19 deaths in the USA have disproportionately clustered in this pandemic enables policy makers to design and target clinical and policy interventions towards facilitating better health outcomes in future crises. Improving US pandemic preparedness and response should start with investing in those disproportionately affected communities and their local organisations, such as local clinics or faith-based institutions, to engage in ongoing public health promotion and data collection, solicit feedback, and communicate with constituents about vaccines and other public health interventions. The potential trade-offs in this pandemic warrant closer, transparent investigation so as to better target such protective measures in future health crises and to develop US job retention schemes and educational policies that can mitigate against unwelcome societal consequences**.** With those investments and others, more US states will be able to match the best-performing nations globally when the next pandemic arises.

## Methods

### Overview

To accomplish our dual objectives—to explore factors potentially associated with COVID-19 prevention and treatment, and to answer five key policy questions—we investigated basic correlates in relation to both SARS-CoV-2 infections and total COVID-19 deaths. Using regression analyses, we controlled for factors that have a known direct and biological connection to SARS-CoV-2 infection and COVID-19 death rates. These factors are generally outside the realm of policy makers in a crisis (eg, age profile, population density, and presence of comorbidities). We explored associations with a broad set of contextual factors that were set before the COVID-19 pandemic, policy responses that policy makers can regulate, and behavioural responses (ie, mask use, mobility, and age-standardised vaccination rate).

All analyses were done with R (version 4.0.3). This study complies with the GATHER recommendations (appendix pp 4–5).^29^ Code used to produce these analyses is available on GitHub.

### Extraction and standardisation of death and infection rates

Death and infection rates were standardised for direct, biological factors that influenced COVID-19 outcomes. For death rates, we standardised for age and presence of comorbidities, and infection rates were standardised for population density.^30^, ^31^ Other factors, such as poverty, income inequality, and race and ethnicity, were not included in the standardisation procedure because there is no direct biological link to COVID-19 outcomes. However, we assessed the association between COVID-19 outcomes, race and ethnicity, poverty, and other key predetermined factors using regression analysis. We also considered indirect effects of race and ethnicity when assessing factors such as access to quality care, key comorbidities, percentage of people without health insurance, educational attainment, and income inequality.^32^

We extracted estimates of daily COVID-19 deaths for all US states from the Institute for Health Metrics and Evaluation's (IHME) COVID-19 modelling database.^33^ Before extraction, these estimates were adjusted for under-reporting, such that they represent total COVID-19 mortality rather than reported death counts.^34^, ^35^, ^36^, ^37^ For each state, we calculated the cumulative death rate from Jan 1, 2020, to July 31, 2022. We used indirect age standardisation to adjust each state's cumulative death rate to reflect the national age pattern (state-specific age-specific death and infection rates were not available). The age-specific death proportions were based on age-specific mortality data as published by the National Center for Health Statistics.^38^ Next, we regressed the logged age-standardised cumulative death rate on the first component of a principal component analysis (PCA) of seven health conditions and risk behaviours that increase the risk of COVID-19 mortality, which included the prevalence of asthma, cancer, chronic obstructive pulmonary disease, cardiovascular disease, and diabetes, along with BMI and smoking prevalence (all of which were age standardised). The first component of the PCA was used, rather than the seven individual prevalence rates, because prevalence rates of these seven variables are highly correlated and our sample size is relatively small, such that using multiple comorbidity prevalence rates could lead to spurious results. This first component of the PCA can be considered a single measure of the underlying presence of comorbidities in each state. We calculated standardised cumulative mortality rates for each state using the fitted model and national health status. These standardised mortality estimates account for state characteristics that directly affect death and are not immediately policy-amenable, thus facilitating comparison across states.

We extracted estimates of daily SARS-CoV-2 infections for all US states from the IHME COVID-19 modelling database.^33^ For each state, we calculated the cumulative infection rate from Jan 1, 2020, to Dec 15, 2021, deliberately excluding the period when infections from the omicron variant led to fundamentally different infection rates. We regressed the logged cumulative infection rate on population density and used the fitted model and national rates to calculate standardised infection rates for each state.

### Assessing factors associated with COVID-19 infections and deaths

A full list of covariate data sources and definitions, including how each variable was measured and specified in our models, can be found in the appendix (pp 6–16). We explored factors that we hypothesised would explain some of the remaining interstate variation in mortality and infection, while controlling for the same features used for standardisation. These factors, which are amenable to public policy, might affect COVID-19 outcomes indirectly but are not believed to have a direct effect on outcomes; specifically, they were pre-COVID-19 state characteristics (such as social and economic factors, public health and health-system capacity, and political leanings) and COVID-19 responses (including policy mandates). We also assessed the association between COVID-19 outcomes and behaviours, such as mask use, vaccination, and physical distancing (which was proxied using metrics of how much a population was moving around relative to before COVID-19).

The social and economic factors explored included poverty rate (proportion of people living below the poverty line in 2019), level of income inequality (Gini coefficient in 2019), mean years of education, race and ethnicity, access to paid sick leave or family leave (existence of state-funded support), and the proportions of people expressing interpersonal trust, trust in the federal government, and trust in the scientific community in the Cooperative Congressional Election Study. To assess the role of race and ethnicity, we used US census data indicating the proportion of people in each state who identified as Black (non-Hispanic), Asian and Pacific Islander (non-Hispanic), American Indian and Alaska Native (non-Hispanic), White (non-Hispanic), or Hispanic. To capture health-system capacity, we used IHME's Healthcare Access and Quality (HAQ) Index; numbers of physicians per capita, health-care workers per capita, and public health employees per capita; public health spending per capita; health spending per capita; and percentage of the population without health insurance. We used two proxies for partisanship at the state level: the political affiliation of the governor and the proportions of the population that voted for the 2020 Republic presidential candidate or the 2020 Democratic presidential candidate. These social, economic, health-system capacity, and political indicators were normalised to the standard normal distribution to facilitate comparison of variables with different units of measurement (except those that were binary).

The policy mandates considered were closures of bars, restaurants, gyms, and schools, mask and vaccine mandates, and stay-at-home orders and gathering restrictions. These data (except vaccine mandates) were extracted from the IHME COVID-19 database.^33^ For each variable, we calculated the percentage of days during which the mandate was in place. Because the number of days that vaccine mandates were in place was unknown, states were assigned a value of 1 if they had a mandate in place as of Feb 10, 2022, a value of 0·5 if the state had previously enforced a mandate but had lifted this requirement before Feb 10, 2022, and a value of 0 if they never had such a mandate. We generated a summary variable indicating a state's overall use of policy mandates, which we called mandate propensity. This summary measure is the first component of the PCA including all the policy mandate variables.

Daily data on population behaviours were also extracted from IHME's COVID-19 database.^33^ We examined three behaviours: mask use, mobility, and vaccination. Mask use data originated from the Premise survey^39^ and were expressed as the daily proportion of adults who always wear a mask when leaving home. Mobility data originated from four sources of mobile phone GPS data and were expressed as a composite metric measuring daily population-level mobility relative to a prepandemic baseline. Vaccine coverage was expressed daily as the proportion of the population that was fully vaccinated. For each variable, we took the mean across days included in the study period.

To assess how each of these factors was associated with cumulative death and infection rates, we regressed each outcome on the controls used for standardisation and each social and economic factor, political variables, health-system capacity, policy mandate, and behavioural variables, separately. The variables of interest were not included collectively because, in some cases, they were quite correlated, and for this exploratory analysis, identifying factors associated with each outcome was the objective. Sensitivity analyses that evaluate the association of COVID-19 outcomes and policy mandates and behaviours controlling for the pre-COVID-19 factors that were shown to have statistically significant relationships with COVID-19 outcomes are included in the appendix (pp 59–60). When assessing factors that were not time-varying, such as poverty rate or partisanship, we used a commonly used log-transformation of the dependent variables. Sensitivity analyses using a negative-binomial generalised linear model are included in the appendix (p 56). When assessing factors that were time-varying, such as the policy and behavioural responses, the dependent variable was the mean of the log-transformed daily rates, a specification that can be shown to mitigate bias that occurs when using cumulative measures, despite the fact that the hypothesised mechanism connecting the dependent and independent variables is immediate. When assessing how COVID-19 outcomes were associated with policy mandates and vaccination coverage, we used shorter periods that focused on when policies or vaccination were used broadly. For policy regressions, we used the period from April 1, 2020, to June 1, 2021, which was the primary period for non-pharmaceutical interventions such as closures and gathering restrictions. For analyses related to vaccination, we started the analysis period on March 15, 2021, because vaccines were not accessible to most people before then. For the rest of the analyses, we used the period from Jan 1, 2020 to July 31, 2022 for deaths (which included the entire period for which we had data), and Jan 1, 2020, to Dec 15, 2021, for infections. The shorter period for infections was chosen to avoid the effect of the omicron variant. The omicron variant led to daily infection rates that were 9·7 times higher than any other point in the pandemic,^33^ and, therefore, is uncharacteristic of the broader pandemic. The variables measuring policy mandate use were correlated with each other. In an attempt to identify which mandates were independently associated with COVID-19 outcomes, we included the mandate intensity variable as a control in all of the regressions exploring the association of the individual mandates. This specification reports the association between a broad use of the policies (ie, the mandate propensity variable) and the potentially additive effect of the specific policies of interest.

### Assessing factors associated with state gross domestic product, employment, and student test scores

We calculated reductions in state gross domestic product (GDP), employment, and student test scores during the pandemic to assess the relationship between these outcomes and policies and behaviours, and to assess trade-offs between health, economic, and educational outcomes. Using monthly data, we estimated GDP and employment relative to the expected non-pandemic value of each. Expected non-pandemic GDP was forecasted up to July 1, 2020, using linear regression based on the 2 years before 2020. For paid employment, the expected value was set to be the average employment per capita from October, 2019, to December, 2019. We made adjustments so that each variable had a standardised composition of economic sectors, such as tourism and agriculture, to ensure fair cross-state comparisons, because some sectors of the economy were more likely to be affected by the pandemic. Test scores were extracted from the National Assessment of Educational Progress, and we calculated the changes in average scores for the mathematics and reading tests that the US Education Department administers to children in school grades four and eight (children aged approximately 9 and 13 years, respectively) for each state between 2019 and 2022.

We regressed relative GDP, employment, and changes in student test scores on the same COVID-19 policy mandate and behaviour response variables described above. Like death and infection rates, these regressions specified the dependent variable as the log-transformation if the independent variable was non-time-varying, and as the mean of the log-transformed monthly values if the independent variable was time-varying. Each regression controlled for state demographics (percentage of the population younger than 20 years, percentage of the population older than 65 years, and average years of education) and the duration and benefit length of each state's unemployment insurance. Using the same regression method, we also assessed the association between these economic, employment, and educational outcomes with infection and death rates. Throughout the study, p<0·05 was considered to indicate statistical significance.

### Uncertainty and sensitivity analyses

We incorporated two sources of uncertainty in our analyses. First, data uncertainty surrounding estimation of infections and deaths was included by conducting each analysis independently 100 times using a set of 100 draws of each outcome measure. These draws were generated by the IHME to capture uncertainty in estimates of COVID-19 outcomes. Second, we incorporated parameter uncertainty for each regression by taking 100 independent draws from the estimated variance–covariance matrix. Because each of the 100 draws from the infections and deaths databases incorporated 100 draws from the variance–covariance matrix, our resulting analysis generated 10 000 draws per estimate. We reported the 95% uncertainty interval (UI) spanning the 2·5th and 97·5th percentile of these 10 000 estimates. We also completed a broad set of sensitivity analyses considering alternative model specifications and estimation methods, using a negative-binomial model, and including alternative controls, which are reported in the appendix (pp 54–63).

### Role of the funding source

The funders of the study had no role in the study design, data collection, data analysis, data interpretation, or the writing of the report.

## Results

**Figure 1:**
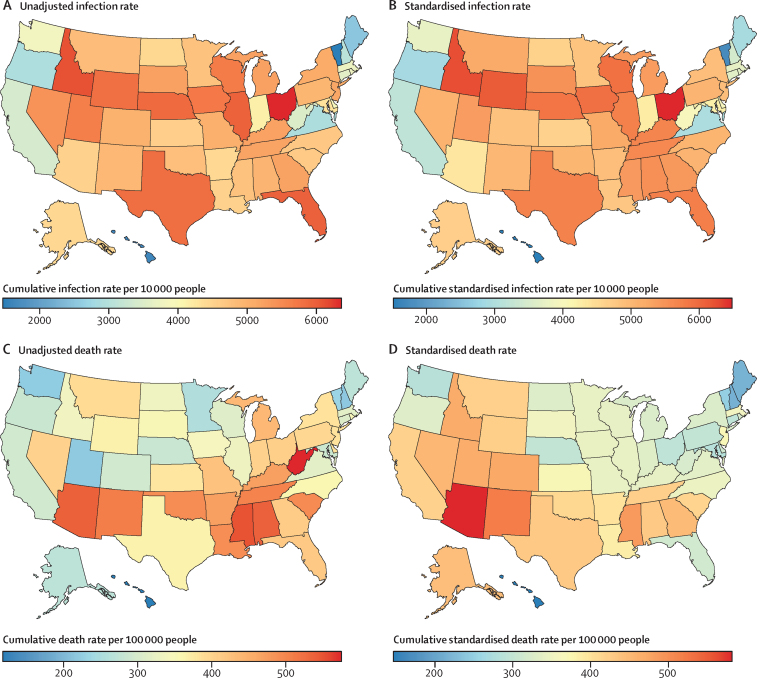
Cumulative COVID-19 infection and death rates by US state Daily infection (Jan 1, 2020, to Dec 15, 2021) and death rates (Jan 1, 2020, to July 31, 2022) that were further adjusted for under-reporting were extracted from the Institute for Health Metrics and Evaluation's COVID-19 database. Standardised cumulative infection rates were adjusted to approximate what the cumulative infection rate would have been if every state had the population density of the USA. Standardised cumulative death rates were adjusted to approximate what the cumulative death rate would have been if every state had the age profile and comorbidity prevalence of the USA. Age standardisation was done using indirect age standardisation. All other standardisation was done with linear regression.

Between Jan 1, 2020, and July 31, 2022, the cumulative COVID-19 death rate in the USA, accounting for under-reporting and lags in reporting, was 372 deaths per 100 000 population (95% UI 364–379). There was substantial variation in the cumulative death rate between the states. West Virginia's cumulative death rate (575 per 100 000 [474–697]), which was the highest across all states, was nearly five times that of Hawaii (119 per 100 000 [108–156]) and more than double that of New Hampshire (218 per 100 000 [195–274]), which were the two lowest rates (figure 1). If all states had the cumulative death rate of New Hampshire, it is estimated that there would have been 504 144 (332 895–590 902) fewer COVID-19 deaths in the USA. Without these additional deaths, the USA would have had a lower cumulative COVID-19 death rate than 12 high-income nations, instead of having the highest rate among all high-income nations (based on IHME COVID-19 projections).

**Figure 2:**
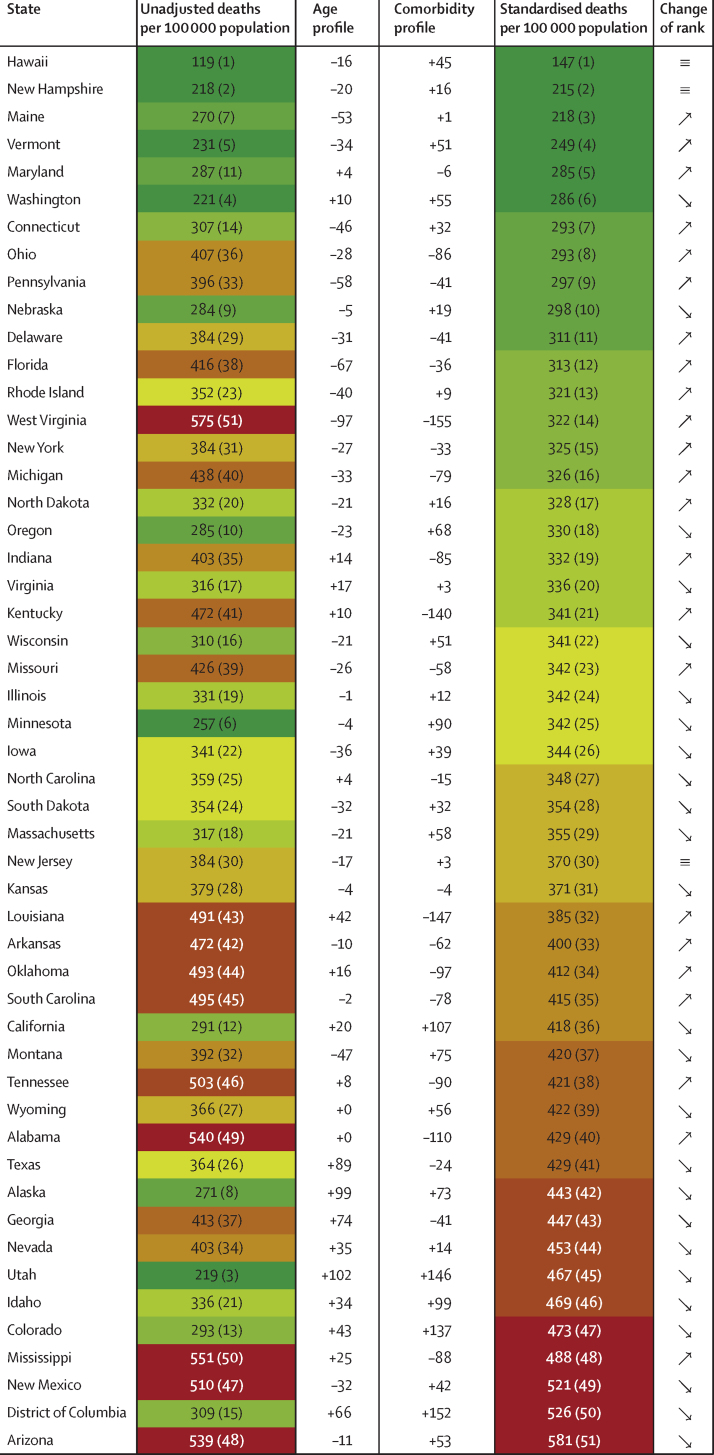
Cumulative death rate standardisation, Jan 1, 2020, to July 31, 2022 Cumulative death rates were adjusted for age profile and prevalence of key comorbidities. The resulting standardised cumulative rates reflect the cumulative death rate if each state had the national age profile and prevalence of comorbidities. Ranks are shown in parentheses. Comorbidities were proxied using the first component of a principal component analysis of asthma, cancer, chronic obstructive pulmonary disease, cardiovascular disease, diabetes, BMI, and smoking prevalence. The values expressed in the age and comorbidity profile columns represent the size of the adjustment (in deaths per 100 000) had a state exhibited the national pattern; positive values indicate that a state is younger or healthier than the nation as a whole, such that standardising the cumulative death rate to the national mean is associated with an increase in the cumulative death rate. The estimates were standardised for age by indirect age-standardisation, while comorbidities were adjusted with use of linear regression.

Crucially, these estimates do not standardise for risks that are known to increase the death rate. Utah, for example, has a healthy population relative to the rest of the USA**,** with low prevalence of key comorbidities that lead to an increase in the cumulative death rate when it is standardised for comorbidities (figure 2). When the cumulative death rate for Utah was adjusted for age and comorbidities, its standardised cumulative death rate (467 per 100 000 [95% UI 385–551]) was 112·8% (95·2–132·1) higher than the unadjusted rate (219 per 100 000 [186–260]). Similarly, West Virginia, which has a relatively young population but high prevalence of key comorbidities, has a standardised cumulative death rate (322 per 100 000 [260–403]) that is 44·0% (38·9–48·7) lower than the unadjusted rate (575 per 100 000 [474–697]). Despite this standardisation, the highest standardised cumulative death rate (in Arizona) was still 399·6% (279·6–477·6) higher than the lowest standardised cumulative death rate (in Hawaii). The states with the lowest standardised cumulative death rates were Hawaii (147 per 100 000 [127–196]), New Hampshire (215 per 100 000 [183–271]), Maine (218 per 100 000 [147–310]), Vermont (249 per 100 000 [163–337]), and Maryland (285 per 100 000 [243–330]). The states and territories with the highest standardised cumulative death rates were Arizona (581 per 100 000 [509–672]), Washington, DC (526 per 100 000 [425–631]), New Mexico (521 per 100 000 [439–609]), Mississippi (488 per 100 000 [412–586]), and Colorado (473 per 100 000 [402–569]).

### Pre-COVID-19 factors: social and economic factors, race, health-care and public health capacity, and politics

**Figure 3:**
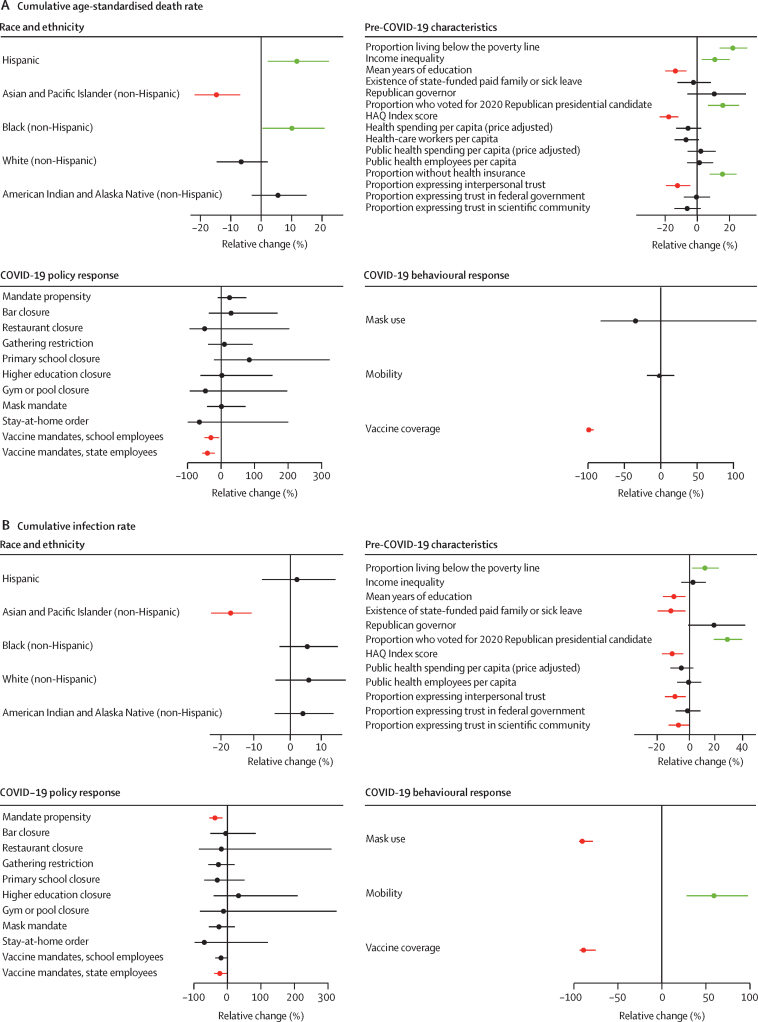
Factors associated with age-adjusted cumulative death rates (A) and infection rates (B) Graphs show the relative change in cumulative age-adjusted deaths or infections per capita that were associated with race and ethnicity (proportions of state population), pre-COVID-19 state characteristics, COVID-19 policy responses, and COVID-19 behavioural responses (values of each are provided in the appendix pp 67–71). For continuous pre-COVID-19 state characteristics, the reported relative change is that associated with a standard deviation increase from the national mean and age-standardised cumulative death rates. For continuous COVID-19 policy measures, the relative change is that associated with a state never having implemented a mandate versus implementing for the entire study period (see appendix pp 67–71 for further detail on all factors). All mortality models included that control and one of the variable of interest factors, meaning that each variable of interest was assessed separately. All infection models include population density as a control and one factor of interest. The comorbidity variable that was used as a control in the cumulative death rate models was constructed as the first principal component of asthma, cancer, chronic obstructive pulmonary disease, cardiovascular disease, diabetes, BMI, and smoking prevalence. The models assessing COVID-19 policy responses (other than mandate propensity) also include an additional control variable that was the first component of all the other policy responses. These estimated associations are not reported. The reported associations for the policy response should be interpreted as additional to the association tied to the mandate propensity variable. The analysis period is tailored to each independent variable (full details are provided in the appendix pp 67–71). Error bars are 95% CIs that account for uncertainty in death or infection data as well as modelling uncertainty. Statistical significance at the 95% level is indicated by green bars (significant increase) or red bars (significant decrease). HAQ=Healthcare Access and Quality.

After controlling for age and comorbidities, cumulative death rates were associated with several pre-COVID-19 social, economic, and race-related or ethnicity-related state characteristics (figure 3A). The states where larger proportions of the population identify as Black (non-Hispanic) or Hispanic had, on average, higher cumulative death rates. An increase of one SD (2·6%) from the mean US poverty rate (12·3%) was associated with a 23·3% (95% UI 14·8–32·5) increase in the cumulative death rate. The same analysis for income inequality found an increase of one SD (0·02) from the mean (0·5) to be associated with an 11·6% (2·7–21·3) increase in the cumulative death rate. An increase from the national mean number of years of education (12·7 years) of one SD (0·3) was associated with a 14·3% (7·1–20·9) decrease in the cumulative death rate. A one SD (0.1) increase from the mean level of interpersonal trust (0·4) was associated with a decrease of 12·9% (4·6–20·4) in the cumulative death rate.

In terms of health-system capacity, an increase of one SD (3·3 points) from the mean of the HAQ Index (86·9 points) was associated with an 18·6% (12·4–24·6) reduction in the cumulative death rate. Increases in the proportion of people without health insurance were also associated with an increase in the cumulative death rate (16·7% [8·5–25·8] for a one SD [0·03] increase over the mean [0·08]). We did not observe statistical associations between the cumulative death rate and state variation in public health spending per capita and number of public health employees per capita. A larger proportion of the population who voted for the 2020 Republican presidential candidate was significantly associated with a higher cumulative death rate, but we found no statistical association between the party affiliation of a state governor and cumulative death rates from COVID-19 (figure 3A).

Many of the same state characteristics that were associated with interstate differences in standardised cumulative death rates were also associated with reductions in the standardised cumulative infection rate (figure 3B). Lower poverty rate and higher HAQ Index, proportion of people expressing interpersonal trust, and mean years of education were all associated with fewer cumulative infections. The only factor that was associated with cumulative death rates but not interstate differences in cumulative infection rates was income inequality. Conversely, the existence of state-funded paid family and sick leave and the proportion of people expressing trust in the scientific community were associated with lower cumulative infection rates but not with lower cumulative death rates.

### COVID-19 response: policy mandates and behaviours

Mandate propensity (a summary measure that captures a state's use of physical distancing and mask mandates) was associated with a statistically significant and meaningfully large reduction in the cumulative infection rate (figure 3B), but not the cumulative death rate (figure 3A). According to this mandate propensity measure (appendix p 47), Oklahoma was the state with the lowest use of such policies. If Oklahoma had the policy response of California, which was estimated to have the largest policy response to COVID-19, we estimate it would have had 32% (95% UI 2–63) fewer infections. In addition to the effect of mandate propensity, vaccine mandates for state employees stood out as having associations with cumulative infection rates and cumulative death rates that were statistically significant.

All three behavioural responses to COVID-19 were statistically associated with lower cumulative infection rates (figure 3B), whereas only vaccine coverage was statistically associated with lower cumulative death rates (figure 3A). If our estimated associations are causal, an effect of this size would suggest that increasing mask use in the state with the lowest level (Wyoming) to the level of the state with the highest level (Hawaii) would result in a 38% (95% UI 8–69) reduction in the cumulative infection rate in the state. If the largest reduction in mobility (California) occurred in the state with the smallest observed reduction in mobility (South Dakota), we estimate that it would bring about a 37% (0–69) reduction in the cumulative infection rate. Additionally, the state with the least number of vaccinated person-days (Alabama) adopting the vaccination uptake of the state with the most vaccinated person-days (Vermont) would lead to a 30% (3–55) decrease in cumulative infections and a 35% (1–61) decrease in cumulative deaths.

**Figure 4:**
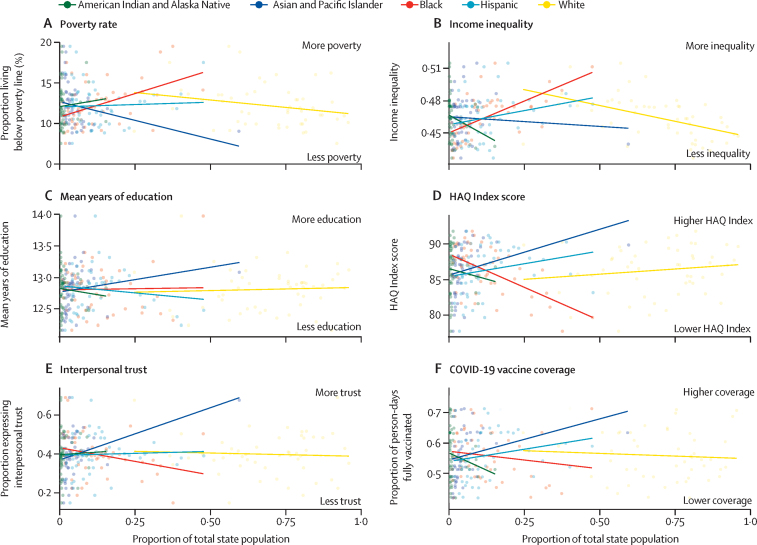
Associations of race or ethnicity with factors shown to be statistically associated with cumulative death rates Graphs show how the proportion of each state identifying as each racial or ethnic category is associated with poverty rate (A), income inequality (B), mean years of education (C), HAQ Index score (D), proportion of people expressing interpersonal trust (E) in 2019, and vaccine coverage (vaccinated person-days per total person-days) from March 15, 2021, to July 31, 2022 (F). HAQ=Healthcare Access and Quality.

**Figure 5:**
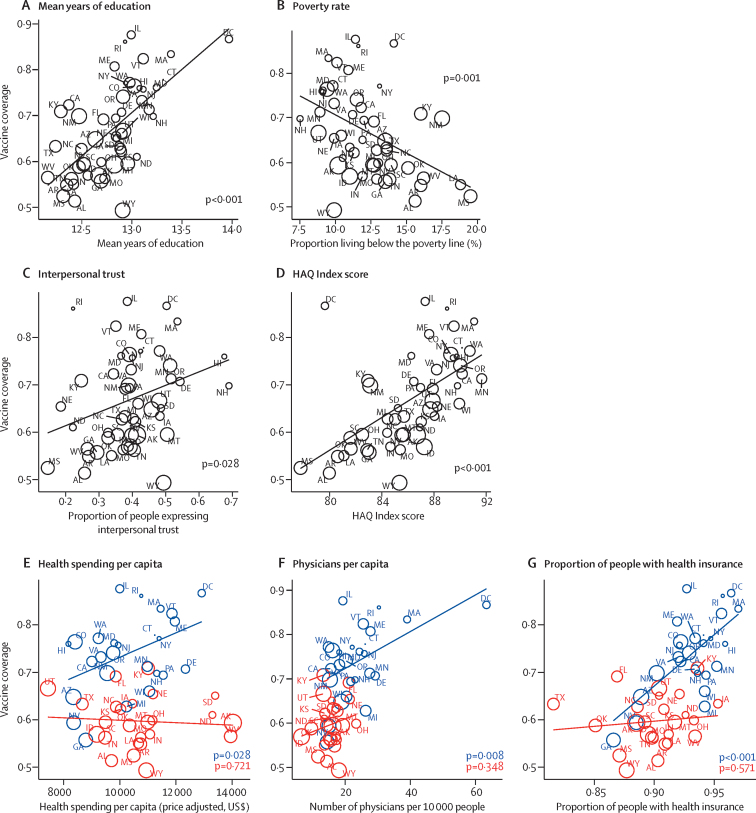
Associations between key pre-COVID-19 state characteristics and vaccine coverage, March 15, 2021, to July 31, 2022 Association of cumulative vaccine coverage (measured as the proportion of person-days a population was fully vaccinated between March 15, 2021, and July 31, 2022) with pre-COVID-19 state and health-system characteristics that were significantly associated with cumulative age-adjusted death rates and cumulative infection rates. For panels A–D, the fitted simple linear regression is shown and p values reflect the statistical significance of the relationship between the pre-COVID-19 factor and vaccine coverage. Initials identify each US state, and the size of a bubble reflects the standardised cumulative death rate for the same period. For panels E–G, states shown in blue voted for the Democratic Party's presidential candidate in 2020, while states shown in red voted for the Republican Party's presidential candidate in 2020; linear relationships between key health system variables and vaccine coverage (and corresponding p values) are shown separately for states that voted for the Democratic (blue) or Republican (red) presidential candidates in 2020. HAQ=Healthcare Access and Quality.

**Figure 6:**
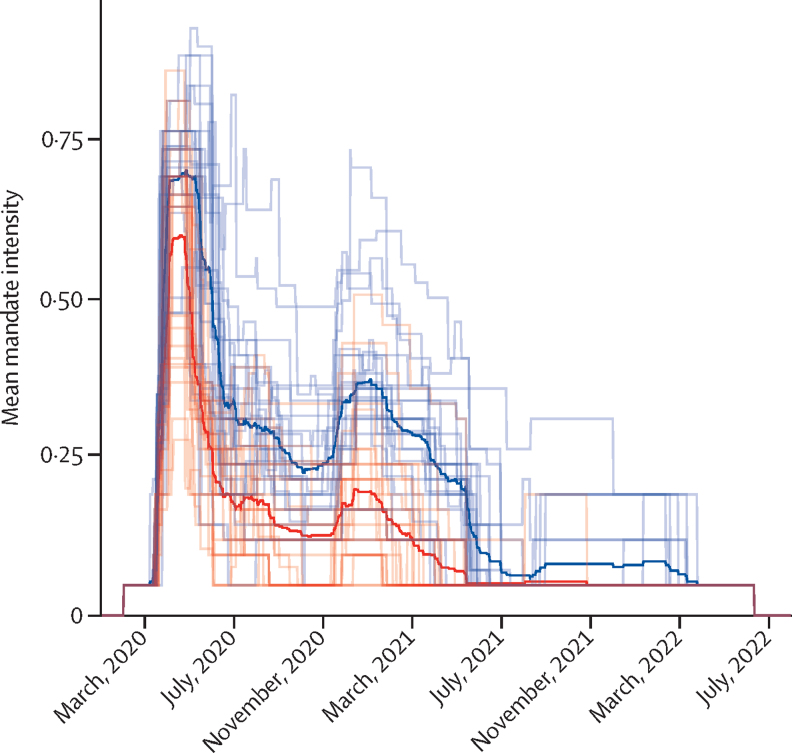
Timing and intensity of mandate adoption in Republican-leaning and Democratic-leaning states, Jan 1, 2020, to July 31, 2022 State-specific measures of mandate intensity over time. The mandate intensity variable combines information across 23 mandates covering seven categories: education closures, travel restrictions, gathering restrictions, stay-at-home orders, business closures, mask mandates, and curfews. Daily mandate variables are binary such that a value of 1 indicates the mandate was in effect for a particular location-day and a 0 indicates the mandate was not in effect on that location-day. To summarise overall mandate intensity, we took the mean by location-day within each of the seven mandate groups and then took the mean of those seven means to generate a single value for each location-day. Mandate intensity is presented as continuous values that vary from 0 to 1, where a 1 means all mandates were in effect on a given location-day and a 0 means no mandates were in effect on that location-day. Blue lines represent states that voted for the Democratic Party's presidential candidate in 2020, with the dark blue line representing the mean of those states. Red lines represent states that voted for the Republican Party's presidential candidate in 2020, with the dark red line representing the mean of those states.

Pre-COVID-19 state characteristics were associated with higher vaccination rates. States with a high poverty rate, low HAQ Index score, and other structural barriers to vaccination have disproportionately large Black (non-Hispanic) and American Indian and Alaska Native populations, while states with higher mean years in education, higher HAQ Index score, and a higher proportion of people expressing interpersonal trust have disproportionally larger Asian and Pacific Islander (non-Hispanic) populations (figure 4). Lower poverty rate, higher HAQ Index, more interpersonal trust, and greater mean years of education were all statistically associated with higher vaccination rates (figure 5). Major health-system characteristics—more health spending per capita, more physicians per capita, and fewer uninsured individuals—were statistically associated with higher vaccination rates in states that voted for the Democratic Party's presidential candidate in 2020, but these health-system characteristics were not associated with higher vaccination rates in states where the majority of voters voted for the Republican Party's presidential candidate (figure 5). Mandates were mainly used at two periods throughout the pandemic, although there was substantial variation across states. In general, states that voted for the Democratic presidential candidate in 2020 used more policy mandates for longer (figure 6).

### Economic and educational trade-offs

**Figure 7:**
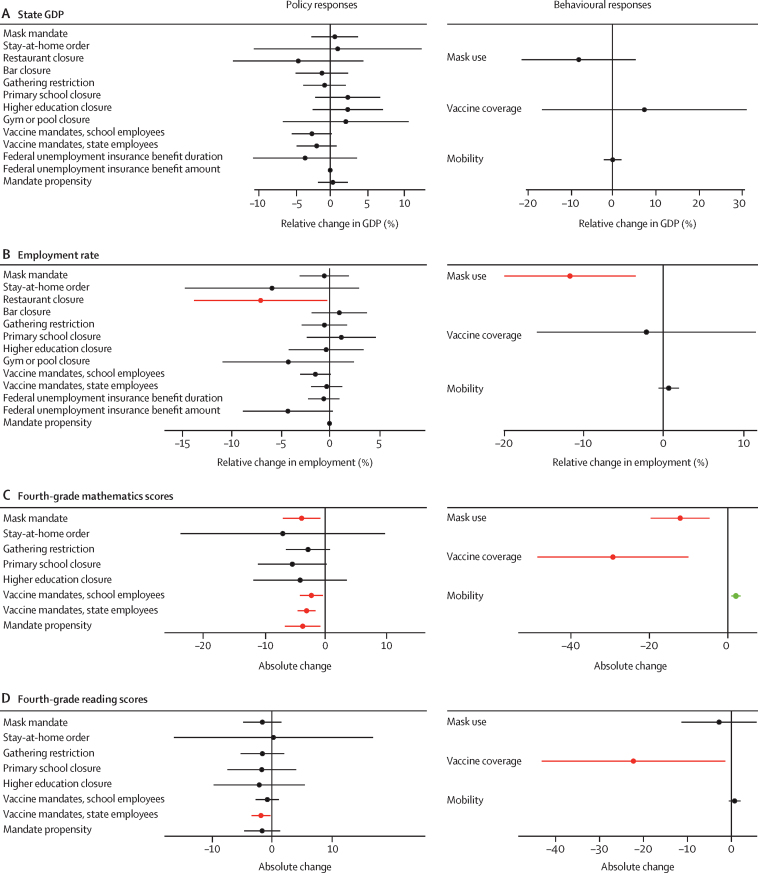
Factors associated with reduction in standardised GDP, employment rate, and mathematics and reading test scores Graphs show estimated associations of COVID-19 policy and behavioural responses with state GDP, sector-standardised and defined as the ratio of expected to actual GDP (A); employment per capita, sector-standardised and defined as the ratio of expected to actual employment (B); changes in fourth-grade mathematics test scores (C); and changes in fourth-grade reading test scores (D). For continuous COVID-19 policy measures, the relative change is that associated with a state never having implemented a mandate versus implementing for the entire study period. Values and more information about interpreting these results are provided in the appendix (pp 72–77). In panels A and B, all regressions include controls for education, proportion of the population older than 65 years, proportion of the population younger than 20 years, mean weekly state unemployment benefits, and mean state unemployment benefit duration. All regressions assessing specific policy interventions also control for mandate propensity and the individual estimates should be interpreted as estimates in addition to the general propensity to impose policy interventions. Error bars are 95% CIs. Statistical significance at the 95% level is indicated by green bars (significant increase) or red bars (significant decrease). GDP=gross domestic product.

**Figure 8:**
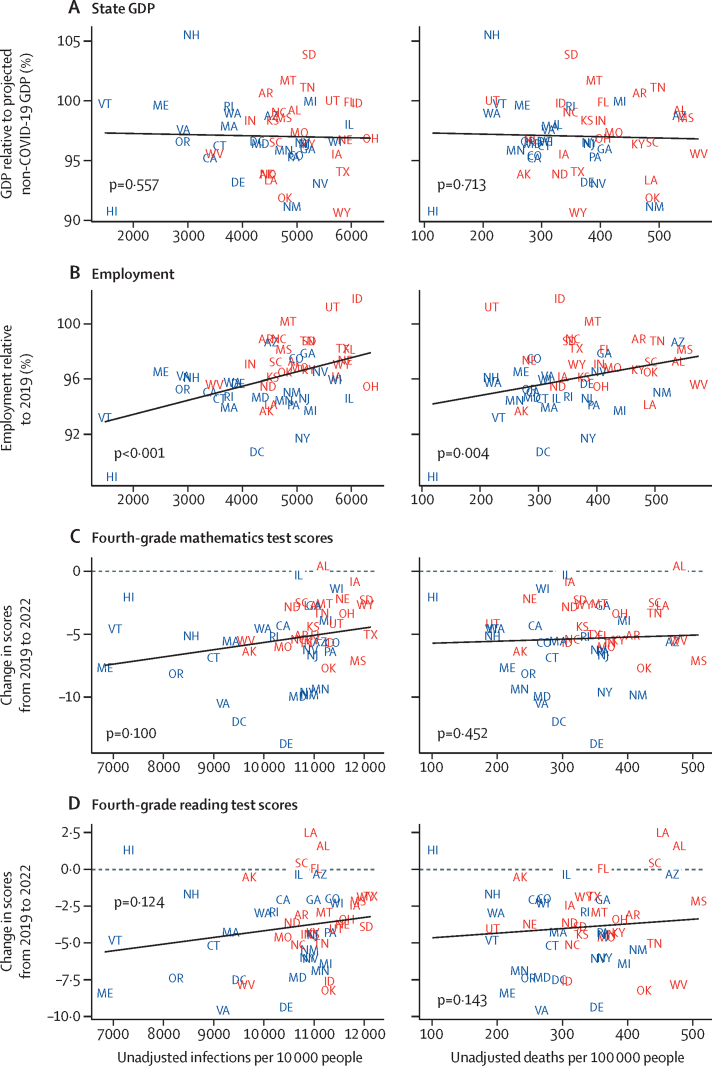
Economic indicators and education scores versus cumulative infection and death rates by state On the vertical axis of each figure is reduction in relative standardised GDP from Jan 1, 2020, to July 31, 2022 (A); relative standardised employment from Jan 1, 2020, to July 31, 2022 (B); change in the mean fourth-grade mathematics scores (possible scores range from 0 to 500) from autumn 2019 to autumn 2022 (C); and change in the mean of fourth-grade reading scores (possible scores range from 0 to 500) from autumn 2019 to autumn 2022 (D). Horizontal axes are cumulative infections per 10 000 people (Jan 1, 2020, to July 31, 2022) and cumulative deaths per 100 000 people (Jan 1, 2020, to July 31, 2022). p values show the statistical significance of the relationship between the two variables, with lines illustrating the association. Initials represent each state; those shown in blue voted for the Democratic Party's presidential candidate in 2020, and those shown in red voted for the Republican Party's presidential candidate in 2020. GDP=gross domestic product.

As with cumulative infection and death rates, there was a great deal of variation across the USA regarding relative decline in state GDP, state employment rate, and mathematics and reading test scores (appendix p 44). Despite this variation, our analysis found no policy mandates or behavioural responses to be systematically associated with reductions in state GDP (figure 7A), and there were no statistically significant correlations between infections or deaths and GDP (figure 8A). Mandated restaurant closures and increased mask use were associated with larger reductions in employment rates (figure 7B). Correspondingly, rates of infections and deaths were significantly correlated with higher employment rate (figure 8B); an increase of 143 deaths (95% UI 80–623) per 100 000 was associated with a one percentage point smaller reduction in employment rates. Increased vaccine coverage and vaccine mandates for state employees were associated with reductions in both fourth-grade mathematics and reading test scores (figure 7C, D). Increased mask use, more mobility relative to other states, mask mandates, vaccine mandates for school employees, and mandate propensity were also associated with reductions in fourth-grade mathematics scores (figure 7C). For mathematics and reading, there were no associations between more deaths and higher test scores, but there was a weak association (p=0·100 and p=0·124, respectively) between more infections and higher fourth-grade mathematics and reading test scores (figure 8C, D).

The absolute and relative performances of US states across key categories are summarised in the appendix (pp 40–42), highlighting the standardised infection and death rates, relative reductions in cumulative GDP and employment rates, and changes in fourth-grade mathematics and reading scores. The sensitivity analyses reinforce the primary findings described above (appendix pp 54–63). Crucially, the regression results illustrated in figure 3 persist when using reported cumulative deaths (rather than cumulative deaths adjusted for under-reporting), non-age-standardised deaths, and when not adjusting for comorbidities.

## Discussion

US states' struggles in the COVID-19 pandemic were not inevitable. The nearly four-fold differences that existed across states in COVID-19 death rates, even when standardised for factors such as age and comorbidities, suggest that lower death rates were achievable. The states with the lowest standardised COVID-19 death rates—Hawaii, New Hampshire, Maine, Vermont, and Maryland—are not confined to a single geographical region, nor did they all have governors from the same US political party. The same is true for the states and territories with the highest standardised death rates—Arizona; Washington, DC; New Mexico; Mississippi; and Colorado. Our analyses of this substantial variation provide important insights into the five key policy questions considered in this paper, which could be helpful to inform US responses to this and future pandemics.

### Social, racial, and economic inequities

Our results show that SARS-CoV-2 infections and COVID-19 deaths disproportionately clustered in US states with lower mean years of education, higher poverty rates, limited access to quality health care, and less interpersonal trust—the trust that people report having in one another. Fewer years of education, limited access to quality health care, and poverty are closely correlated with one another, as is, to a lesser extent, low interpersonal trust (appendix p 43). Accordingly, these factors are best viewed as a package of traits associated with states that had worse outcomes in the pandemic, rather than as individual contributors to COVID-19 outcomes.

This package of traits exists in US states and territories with a higher percentage of people identifying as Black and with higher percentages of people who voted for the 2020 Republican presidential candidate, such as Texas, Mississippi, and Alabama (appendix pp 45–46). While this category of states is correlated with higher prevalence of key comorbidities that worsen COVID-19 outcomes (eg, diabetes and obesity), it is otherwise distinct in terms of the factors assessed in this study. For example, this subset of states is associated with income inequality, but that is not true of the overall category of states with a higher percentage of people who voted for the 2020 Republican presidential candidate. Conversely, this subset of states is associated with higher rates of people without health insurance, but that is not true of the overall category of states with a higher percentage of people who identify as Black.

As such, the poor performance of the USA in the COVID-19 pandemic can be described as the product of a syndemic—one centred around the combination of race and politics. Syndemic is a term that scholars use to refer to the phenomenon when pre-existing local health conditions and socioeconomic disparities drive the spread of disease and worsen its adverse outcomes. The term syndemic was first used to describe the simultaneous epidemics of substance use, violence, and HIV, which were intertwined and mutually reinforcing in the local context of inner-city Hartford, CT, USA.^40^, ^41^, ^42^, ^43^ This concept also applies to the category of US states where COVID-19 has done its greatest damage.

Social, racial, and economic inequities and poor COVID-19 outcomes are linked in various ways. Poverty, income inequality, and low educational attainment keep people from living in healthy neighbourhoods and residences, maintaining healthy behaviours, and affording health care.^44^ Individuals deemed to be essential workers have, on average, lower incomes and education levels and were less likely to be able to work remotely and maintain physical distancing in this pandemic.^45^ Essential workers are also disproportionately Black, Hispanic, or Native American, and are more likely to live in multigenerational households where SARS-CoV-2 spreads more easily and to face systemic discrimination and socioeconomic disadvantages in accessing health-care services.^44^, ^46^, ^47^, ^48^ Many of the worst-performing states and territories in our study are also those with the highest populations of people identifying as Black (Washington, DC; Mississippi; and Georgia), Hispanic (Arizona and New Mexico), or American Indian and Alaska Native (Idaho, Nevada, Alaska, Wyoming, and Montana).^49^ States with higher percentages of Black Americans were associated with higher income inequality, more people living below the poverty line, lower trust in the scientific community, less access to quality health care, and lower vaccine coverage (figure 4; appendix pp 45–46). States with larger Hispanic populations were most closely linked to less health spending and fewer hospital beds (appendix pp 45–46). The proportion of people identifying as American Indian and Alaska Natives in most US states might be too small to show a national-level statistical association with worse COVID-19 outcomes in this study, but they are the group that has had the steepest overall declines in life expectancy over 2020 and 2021.^50^

Our results indicate that many of the same factors associated with rates of COVID-19 infections and deaths—lower poverty rates, more time in education, greater access to quality health care, and more interpersonal trust—are also associated with increased COVID-19 vaccine coverage. Vaccine coverage is not just a matter of securing adequate supply of doses; it also depends on overcoming structural barriers to administration, such as insufficient access to vaccination sites and an inability to get time off from work or caregiving, as well as demand constraints.^51^ The US federal government and many states were slow to adequately deploy mobile clinics to vaccinate essential workers and to administer doses at rural health clinics and federally qualified health centres, where people with low income and members of Black, American Indian and Alaska Native, and Hispanic Americans disproportionately get their medical care.^52^, ^53^ Only a quarter of US states included specific strategies to encourage vaccination in these minority racial and ethnic communities as part of their initial COVID-19 vaccine roll-out plans, despite ample research showing that those communities have historical reasons to mistrust public health campaigns.^54^, ^55^

Declining economic conditions among lower-income Americans without a university degree have also weakened bonds of interpersonal trust—civic organisations, family bonds, and unions.^56^ Our results suggest interpersonal trust (ie, trust in others) has played an outsized role in the COVID-19 pandemic, as it has in other epidemics where scientific uncertainties are great and public confidence is easily undermined.^57^, ^58^, ^59^, ^60^ Having greater interpersonal trust motivates individuals to protect others in the community and reduces their fear of being misled and exploited by their peers. States with a higher percentage of people who voted for the 2020 Republican presidential candidate are associated with lower interpersonal trust in our study results. Trust has long been partisan in the USA, with citizens reporting less trust in government when the president comes from a different party than their own.^61^ Without the public's trust, the US Government, as in many other democratic societies, is limited in its ability to compel vaccination outside of public schools, the military, and other government employment.^62^, ^63^, ^64^, ^65^

### Health-care access and public health capacity

Our analyses provide evidence that health-care access is associated with better COVID-19 outcomes. Higher access to quality health care^66^ and a smaller proportion of uninsured individuals were both, on average, associated with fewer cumulative infections and lower total COVID-19 deaths. Higher public health spending and more public health personnel per capita were not associated, on average, with fewer cumulative COVID-19 deaths or SARS-CoV-2 infections at the state level in our analysis. This analysis, however, does not capture the consequences of the “uneven patchwork”^67^ of US state and local public health capacities that were experienced at the federal level during the pandemic, especially with regard to poor data, threadbare information systems, and delayed reporting of infections, hospitalisations, and deaths.

Access to care—insurance coverage and the number and proximity of health-care facilities and providers—and quality of care are major influences on health behaviours, which might explain the link to cumulative infections in this pandemic. Having a regular physician helps, because doctors are often the most trusted messengers on vaccination and avoiding SARS-CoV-2 exposure and transmission,^68^, ^69^, ^70^ as well as being sources of improved treatment outcomes. However, there are considerable disparities in access to quality health care, especially in poorer US regions.^71^ The USA does not provide universal health coverage, and its health-care costs are ever-increasing. At the onset of the pandemic, nearly a third of US states had not expanded Medicaid coverage under the Affordable Care Act,^72^ the joint federal and state programme that helps cover medical costs for individuals with low incomes. At different points in the pandemic, the US federal government provided masks, testing, and vaccines for free to the public, but treatment costs were still high, especially in hospital settings and for Americans who lost their jobs along with employer-based insurance coverage during the pandemic.^73^, ^74^

### Partisanship

Our study suggests that partisanship made a nuanced contribution to state differences in COVID-19 outcomes. Our results do not show, on average, a statistically significant association between COVID-19 death rates and the political affiliation of the state governor. The highest elected officials in half of the ten states with the lowest standardised cumulative death rates—Vermont, New Hampshire, Maryland, Ohio, and Nebraska—were Republicans, with the remaining five best-performing states led by Democrats. On the other hand, our results show that states where a greater fraction of the population voted for the Republican Party's 2020 presidential candidate, on average, had more infections and more total COVID-19 deaths for the entire study period. Those findings are consistent with other studies.^14^, ^15^, ^16^, ^75^, ^76^, ^77^

Partisanship might mediate the relationship between health systems and COVID-19 outcomes (figure 5). In states that voted for the Democratic Party's 2020 presidential candidate, vaccine coverage was associated with stronger health systems (eg, more health workers and physicians, fewer people without insurance). In the states that voted for the Republican Party's 2020 presidential candidate, however, we found no association connecting health system factors with adoption of protective behaviours, such as vaccination. In other words, our results suggest that robust health systems mattered most in this pandemic in the states where the public was willing to use them. An important exception emerged in our results: we found an association between strong health systems and vaccine coverage in Republican-leaning states with higher mean years of education.

Our results suggest that partisanship reduced the use of protective mandates and behaviours. By late March, 2020, at least 90% of US states had at least one of the following policy mandates in effect: school closures, gathering restrictions, stay-at-home orders, and closures of bars, restaurants, and gyms. A year later, less than 10% of states had at least one of those same mandates in effect. In the interim, as the pandemic persisted and vaccines became available, mandate use became more heterogeneous among US states, and differences emerged between Democratic-leaning and Republican-leaning states. After that initial collective effort by most US states to curb the transmission of SARS-CoV-2, fatigue, protests, and election-year politics began to arise in April, 2020 (figure 6).^78^ The willingness to implement protective mandates and adopt behaviours became the lens through which many Americans and elected officials reacted to the COVID-19 pandemic.^79^ During this period, states with a Republican governor and that voted for the Republican Party's 2020 presidential candidate imposed fewer mask and vaccine mandates, had shorter stay-at-home orders and business closures, and exhibited higher levels of mobility and less mask use and vaccine coverage. When the delta variant (B.1.617.2) of SARS-CoV-2 emerged in the winter of 2020–21 and SARS-CoV-2 infections and COVID-19 deaths surged, more Democratic-leaning states responded by reinstituting protective mandates than states that voted for the Republican Party's 2020 presidential candidate (figure 6).

### Policy mandates and behaviours

The period in which most US states deployed at least some policy mandates was approximately 1 year long. Our results suggest that states that implemented and maintained more policy mandates, including those meant to encourage or require mask use, vaccination, and physical distancing, had, on average, lower infection rates during the period in which those mandates were in effect. The pathway through which these mandates affect transmission appears to be the public's adoption of protective behaviour. The states that implemented and maintained more mandates were statistically associated, on average, with higher mask use and greater vaccine coverage rates, which in turn were associated with fewer infections. Vaccine coverage also had a large association with US state variation in death rates during the period when vaccines were available.

Most states implemented the mandates assessed in this study during the same period, and those mandates interacted and worked synergistically in the pandemic. We found that few of these individual mandates alone were associated with better COVID-19 outcomes beyond the reduction achieved by the package of non-pharmaceutical interventions. The exception was vaccine mandates for state employees, which were statistically associated with lower rates of infections and with reductions in cumulative death rates. The category of state and local government employees includes millions of vaccine-eligible adults.^80^

The greater association of mandates with infections than with deaths highlights that factors beyond infection influenced death rates in this pandemic. Poverty rates, access to quality health care, time in education, and proportion of people with health insurance were all associated with deaths and independently affected the infection-fatality rate. At-risk, older, and less healthy individuals might still have adopted behaviours to avoid infection, such as mask use, or avoiding gatherings—even without states requiring them to do so—obscuring the effects of mandates on deaths.

### Economic and educational trade-offs

Our results provide no evidence of a trade-off between a state having a relatively strong economy and COVID-19 health outcomes in this pandemic. There were trade-offs between fewer infections and higher employment rates, and weaker evidence of a trade-off with higher fourth-grade mathematics and reading test scores.

Two competing critiques emerged over the US COVID-19 response. One prevailing viewpoint was that the health benefits for states that more heavily deployed protective mandates—ie, non-essential business and school closures, mask mandates, and stay-at-home orders—were not worth the disruption to lives, livelihoods, and children's education.^27^, ^81^ The other perspective was that a functioning US economy and school system depended on infection and death rates falling, so there were no trade-offs between health, jobs, and economic and educational policies.^82^, ^83^, ^84^ Our results suggest both critiques are partially correct.

We found no associations between GDP and most health mandates, lower infections, or fewer total deaths in the pandemic, indicating that the economy was neither hindered nor helped by interstate differences in COVID-19 health mandates and outcomes. State differences in employment in the pandemic were statistically associated with mandates on restaurant closures, but not with other business closures or gathering restrictions. The pandemic, however, can affect employment even in the absence of such policy mandates by reducing people's willingness to work (labour shocks), lowering or changing consumption patterns (eg, shifting commerce from in-person businesses to online retailers), or creating uncertainty that reduces investment.^85^ In our study, higher employment was associated with states with less mask use, more infections, and greater COVID-19 deaths during the study period. These results suggest that job losses might have been less severe in states where the population was more willing to incur the risks of SARS-CoV-2 transmission and severe COVID-19 outcomes in order to engage in in-person commercial and retail activities.

The COVID-19 pandemic coincided with substantial declines in US educational performance, but our results do not indicate that those learning losses were systematically associated with state-level primary school closures. 20 years of progress in fourth-grade mathematics and reading scores were reversed between 2019 and 2022, according to the 2021 National Assessment of Educational Progress examination, a nationally comparable test that the US Education Department administers every 2 years to 9-year-old children. Yet, California, a state with long school closures during the pandemic, had test score declines similar to or smaller than those in Florida and Maine, states with low rates of school closures.^86^, ^87^ It is likely, however, that our state-level estimates of school closures do not fully capture the diversity in school closure and reopening decisions that occurred within states at the district levels. Our results suggest that state declines in fourth-grade mathematics scores were associated with the intensity of mandates deployed and with mandates unrelated to school closures (mask mandates and vaccine mandates for state and school employees). Declines in fourth-grade mathematics scores were also associated with higher mask use, less mobility, and higher vaccine coverage, as well as showing a weak association with lower infection rates without reaching statistical significance at the 5% level. One possible explanation is that in states where the public and elected officials were more cautious about SARS-CoV-2 transmission generally, more parents might have elected to keep children in remote schooling, irrespective of state requirements. Another possibility is that mask and vaccine mandates for state and school employees affected school attendance and closures in ways that our study is not designed to measure. Several studies have found that remote schooling was associated with lower test scores, in mathematics especially.^88^ Other studies showed that Black and Hispanic students and those from low-income households were in fully remote schooling longer and, on average, had steeper declines in mathematics test scores, widening the already large and persistent racial and economic inequities in US education.^89^, ^90^, ^91^

### Limitations

This study was subject to several limitations. Assessing causal relationships using observational data is challenging and sometimes impossible. This study was not designed to definitively determine causality. Instead, in this study we tested the association of key social, economic, and health-care capacity factors with cumulative death and infection rates, controlling for biological factors such as age, comorbidities, and population density. These analyses were completed independently of each other because the factors were correlated, as unmeasured confounding connects them in ways that our study was not able to parse. Some factors might be mediated or modified by other unobserved factors. Moreover, person-level data would allow for more precise identification of relationships between pre-COVID-19 contextual factors, state policy responses, individual behaviours, and COVID-19 outcomes. Because this analysis is completed at the state level using ecological data, the evidence presented in this Article cannot prove that these factors have a causal relationship with COVID-19 outcomes, despite each factor having a statistically significant relationship and a plausible pathway. Although we assessed policy mandates and strategic behaviours—including mask use, vaccination rates, and mobility—that are more directly connected to the COVID-19 outcomes, they, like the other factors, are subject to the modifiable areal unit problem. While this problem is associated with variation within states with districts and localities imposing different mandates, it might cloud the impact of the state effect that we are estimating.

Our analyses were also based on cumulative estimates aggregated across time. This strategy makes it easier to assess broad levels of association between characteristics and responses but makes directionality challenging to assess. Moreover, mistiming of policy interventions or behaviours could lead our regression approach to estimate no effect on COVID-19 outcomes when there in fact is one. Finally, total COVID-19 infections and deaths are imperfectly measured due to under-reporting. To address this deficit, we used uncertainty drawn from the IHME COVID-19 database.

### Implications for policy

COVID-19 magnified the polarisation and persistent social, economic, and racial inequities that already existed across US society, but the next pandemic threat need not do the same. Recognising the local contexts in which SARS-CoV-2 infections and COVID-19 deaths in the USA have disproportionately clustered in this pandemic enables policy makers to design and target clinical and policy interventions towards facilitating better health outcomes in future crises.^92^ Our results underscore the need for policies such as paid family and sick leave and expanded Medicaid and insurance coverage,^84^, ^85^ which would help individuals with low incomes to get vaccinated, obtain effective treatment, and take the protective measures necessary to protect themselves in future pandemics. Earlier adoption of targeted, community-based efforts, such as the door-to-door vaccine ambassador programme used in Baltimore, MD, could help to narrow disparities associated with race, ethnicity, and political leaning in future vaccination campaigns.^93^, ^94^ One way to improve vaccine uptake generally among partisan and marginalised groups would be for states to invest in community-based organisations, such as local clinics or faith-based institutions, to engage in ongoing public health promotion and data collection, solicit feedback, and communicate with constituents during the COVID-19 crisis and beyond.^95^, ^96^ In future crises, federal and state governments could include in their public health campaigns emissaries, such as business leaders, community leaders, or talk-show hosts, who might appeal to those who doubt the current government on the safety and efficacy of vaccines and pandemic prevention.^97^ Clear, transparent, and timely communication can help to build public trust in a crisis.^81^, ^82^

Our study suggests that the policy mandates and protective behaviours adopted in this pandemic were effective in reducing SARS-CoV-2 infections but might have been associated with employment and educational trade-offs in some cases. These potential trade-offs warrant closer investigation so as to better target such protective measures in future crises and to develop US job retention schemes and educational policies that can mitigate against unwelcome societal consequences.^98^ In the meantime, the data indicate that more focused support might be needed to help the lowest-achieving students catch up and address US educational achievement gaps that have widened significantly over this pandemic.

### Conclusion

The Institute of Medicine has described public health as “what we as a society do collectively to assure the conditions in which people can be healthy”.^99^ Trust in that collective response was low before the pandemic and is lower still after the nation's struggles with COVID-19.^68^ An essential step in rebuilding trust in US public health and future pandemic responses is being transparent about the political contexts and social, economic, and racial inequities that might have contributed to US struggles in this crisis, and to identify where the economic and educational trade-offs might have been too great to justify the protective measures adopted. Our study suggests that where US states have been able to confront these structural inequities, deploy science-based measures, and mobilise the social solidarity that does exist in America, those states have been fully able to match the best-performing nations globally in this pandemic.

## Data sharing

Data used in this analysis are available to download from the Global Health Data Exchange website.

## Declaration of interests

CA reports support for the current work from the Benificus Foundation. ADF reports other financial or non-financial support from Johnson & Johnson, Sanofi, and SwissRe outside of the submitted work. NF reports financial support from WHO and Gates Ventures outside of the submitted work. All other authors declare no competing interests.
