# Supplementary material for: Assessing COVID-19 pandemic policies and behaviours and their economic and educational trade-offs across US states from Jan 1, 2020, to July 31, 2022: an observational analysis
Source: Lancet. 2023 Apr 22;401(10385):1341–60. doi: 10.1016/S0140-6736(23)00461-0 (PMC10036128; doi:10.1016/S0140-6736(23)00461-0)
Supplement: Supplementary appendix [file mmc1.pdf]

THE LANCET

Supplementary appendix

This appendix formed part of the original submission and has been peer reviewed. We post it as supplied by the authors.

Supplement to: Bollyky TJ, Castro E, Aravkin AY, et al. Assessing COVID-19 pandemic policies and behaviours and their economic and educational trade-offs across US states from January, 2020, to July, 2022: an observational analysis. *Lancet* 2023; published online March 23. https://doi.org/10.1016/S0140-6736(23)00461-0.

Appendix 1: Supplemental methods and results to "Assessing COVID-19 policies and behaviours and their economic and educational trade-offs across US states from January 1, 2020, to July 31, 2022: an observational analysis"

This appendix provides further methodological and supplementary results for "Assessing COVID-19 policies and behaviours and their economic and educational trade-offs across US states from January 1, 2020, to July 31, 2022: an observational analysis".

Table of Contents

Appendix 1: Supplemental methods and results to "Assessing COVID-19 policies and behaviours and their economic and educational trade-offs across US states from January 1, 2020, to July 31, 2022: an observational analysis" 1

Section 1: List of abbreviations 2

Section 2: GATHER criteria 4

2.1 GATHER checklist 4

Section 3: Data inputs 6

3.1 Death model inputs 6
3.2 Infection model inputs 13
3.3 GDP model inputs 17
3.4 Employment model inputs 26
3.5 Education model inputs 35
3.6 Dependent variables by state 40
3.7 Correlation among independent variables 43
3.8 Reduction in economic indicators and education scores by state 44
3.9 Syndemic framework for COVID-19 45
3.10 Mandate propensity 47

Section 4: Methods 48

4.1 Modeling COVID-19 Deaths 48
4.2 Modeling COVID-19 Infections 50
4.3 Modeling GDP 51
4.4 Modeling Employment 52
4.5 Modeling Education 53

Section 5: Sensitivity Analyses 54

5.1 Infection and death results using the logged cumulative rate as the dependent variable for all models 54
5.2 Infection and death results using negative binomial model 56
5.3 Infection and death results including poverty as a control for COVID-19 policies and behaviors 57
5.4 Infection and death results including the first principal component of a PCA of all statistically significant pre-COVID-19 factors as control variables for COVID-19 policies and behaviours 59
5.5 Death results without control variables 61
5.6 Death results with no control variables and no age standardization of cumulative death rate 62
5.7 Death results without control variables, dependent variable specified as cumulative reported deaths per capita 63
5.8 Reporting E-Values for unmeasured confounding 64

Section 6: Supplementary Results 67

6.1 Factors associated with age-adjusted cumulative death rates and cumulative infection rates 67
6.2 Factors associated with reduction in standardised GDP, employment rate, and mathematics and reading test scores 72

Section 1: List of abbreviations

**Abbreviation**	**Full phrase**

**ACA**	Affordable Care Act
**BMI**	Body mass index
**CDC**	United States Centers for Disease Control and Prevention
**COPD**	Chronic obstructive pulmonary disease
**COVID-19**	Coronavirus disease 2019
**IHME**	Institute for Health Metrics and Evaluation
**GATHER**	Guidelines for Accurate and Transparent Health Estimates Reporting
**GDP**	Gross domestic product
**NAEP**	National Assessment of Educational Progress
**NCHS**	National Center for Health Statistics
**NPI**	Non-pharmaceutical intervention
**PCA**	Principle component analysis
**SARS-Cov-2**	Severe acute respiratory syndrome coronavirus 2
**WHO**	World Health Organization

Section 2: GATHER criteria

This study complies with GATHER recommendations. We have documented the steps in our analytical procedures and detailed the data sources used. See Table S1 for the GATHER checklist. The GATHER recommendations can be found on the GATHER website.

2.1 GATHER checklist

Checklist for compliance with the Guidance for Accurate and Transparent Health Estimates Reporting (GATHER)

Item #	Checklist item	Reported on page #
Objectives and funding		
1	Define the indicator(s), populations (including age, sex, and geographic entities), and time period(s) for which estimates were made.	Summary. Main Text: Introduction, Methods
2	List the funding sources for the work.	Summary. Main Text: Acknowledgements and declarations.
Data Inputs		
For all data inputs from multiple sources that are synthesized as part of the study:		
3	Describe how the data were identified and how the data were accessed.	Main Text: Methods. Supplementary Appendix: Section 3.
4	Specify the inclusion and exclusion criteria. Identify all ad-hoc exclusions.	Main Text: Methods. Supplementary Appendix: section 3.
5	Provide information on all included data sources and their main characteristics. For each data source used, report reference information or contact name/institution, population represented, data collection method, year(s) of data collection, sex and age range, diagnostic criteria or measurement method, and sample size, as relevant.	Supplementary Appendix: Section 3. Main characteristics of data, metadata, and/or NIDs available through: http://ghdx.healthdata.org/
6	Identify and describe any categories of input data that have potentially important biases (e.g., based on characteristics listed in item 5).	Main text: Limitations section. Supplementary appendix: Section 4.
For data inputs that contribute to the analysis but were not synthesized as part of the study:		
7	Describe and give sources for any other data inputs.	Supplementary Appendix: Section 3.
For all data inputs:		
8	Provide all data inputs in a file format from which data can be efficiently extracted (e.g., a spreadsheet rather than a PDF), including all relevant meta-data listed in item 5. For any data inputs that cannot be shared because of ethical or legal reasons, such as third-party ownership, provide a contact name or the name of the institution that retains the right to the data.	Available through: http://ghdx.healthdata.org/
Data analysis		
9	Provide a conceptual overview of the data analysis method. A diagram may be helpful.	Main text: Methods.

10	Provide a detailed description of all steps of the analysis, including mathematical formulae. This description should cover, as relevant, data cleaning, data pre-processing, data adjustments and weighting of data sources, and mathematical or statistical model(s).	Main text: Methods. Supplementary Appendix: Section 4
11	Describe how candidate models were evaluated and how the final model(s) were selected.	Supplementary Appendix: Sections 4 & 5
12	Provide the results of an evaluation of model performance, if done, as well as the results of any relevant sensitivity analysis.	Supplementary Appendix: Sections 4 and 5
13	Describe methods for calculating uncertainty of the estimates. State which sources of uncertainty were, and were not, accounted for in the uncertainty analysis.	Main Text: Methods. Supplementary Appendix: Section 4.
14	State how analytic or statistical source code used to generate estimates can be accessed.	Available through: https://github.com/ihmeuw
Results and Discussion		
15	Provide published estimates in a file format from which data can be efficiently extracted.	Available through: http://ghdx.healthdata.org/
16	Report a quantitative measure of the uncertainty of the estimates (e.g. uncertainty intervals).	Main text: Results
17	Interpret results in light of existing evidence. If updating a previous set of estimates, describe the reasons for changes in estimates.	Main Text: Discussion
18	Discuss limitations of the estimates. Include a discussion of any modelling assumptions or data limitations that affect interpretation of the estimates.	Main Text: Limitations

Section 3: Data inputs

3.1 Death model inputs

Description of all dependent and independent variables explored in the death regression analysis, including mathematical transformations, relevant time period, control variables, and data sources.

Variable name	Definition	Category	Transformation	Analysis Time Period	Dependent variable transformation	Controls	Source
**Deaths**	Age-standardised cumulative deaths per capita	Dependent variable	Log	NA	NA	NA	IHME COVID estimate, based on reporting from JHU
**Population density**	Proportion of the population living in an area with at least 500 ppl per sq km, 2019	Control	Normalize	NA	NA	NA	US Census Bureau; TIGERLS
**Comorbid conditions and risk factors**	First principal component of a PCA of the following conditions and behaviors: asthma, cancer, chronic obstructive pulmonary disease (COPD), cardiovascular disease, diabetes, body-mass index (BMI), and smoking. Each condition was expressed as the age-standardised prevalence.	Control	Normalize	NA	NA	NA	GBD 2019
**Hispanic**	Proportion of the population identifying as Hispanic	Race/ Ethnicity	Normalize	1/1/20 - 7/31/22	log(cumulative infections per capita)	comorbidities	US Census Bureau
**Non-Hispanic Asian**	Proportion of the population identifying as non-Hispanic and Asian or Pacific Islander	Race/ Ethnicity	Normalize	1/1/20 - 7/31/22	log(cumulative infections per capita)	comorbidities	US Census Bureau
**Non-Hispanic Black**	Proportion of the population identifying as non-Hispanic and Black	Race/ Ethnicity	Normalize	1/1/20 - 7/31/22	log(cumulative infections per capita)	comorbidities	US Census Bureau

**Non-Hispanic White**	Proportion of the population identifying as non-Hispanic and White	Race/ Ethnicity	Normalize	1/1/20 - 7/31/22	log(cumulative infections per capita)	comorbidities	US Census Bureau
**Non-Hispanic American Indian**	Proportion of the population identifying as non-Hispanic and American Indian or Alaska Native	Race/ Ethnicity	Normalize	1/1/20 - 7/31/22	log(cumulative infections per capita)	comorbidities	US Census Bureau
**Poverty**	% of the population living below the poverty line, 2019	Pre-COVID state characteristic	Normalize	1/1/20 - 7/31/22	log(cumulative infections per capita)	comorbidities	GBD 2019
**Income inequality**	Gini coefficient, 2019	Pre-COVID state characteristic	Normalize	1/1/20 - 7/31/22	log(cumulative infections per capita)	comorbidities	US Census Bureau
**Health access and quality**	Health access and quality index (HAQI), 2019	Pre-COVID state characteristic	Normalize	1/1/20 - 7/31/22	log(cumulative infections per capita)	comorbidities	GBD 2019
**Public health FTEs**	Public health full-time equivalent (FTE) hours per capita, 2019	Pre-COVID state characteristic	Normalize	1/1/20 - 7/31/22	log(cumulative infections per capita)	comorbidities	US Census: State Government Employment & Payroll
**Public health spending**	Price-adjusted public health spending per capita, 2019	Pre-COVID state characteristic	Normalize	1/1/20 - 7/31/22	log(cumulative infections per capita)	comorbidities	State Health Access Data Assistance Center (SHADAC)
**Health spending**	Price-adjusted health spending per capita, 2019	Pre-COVID state characteristic	Normalize	1/1/20 - 7/31/22	log(cumulative infections per capita)	comorbidities	CMS State Health Expenditure Accounts
**Hospital beds**	Hospital beds per capita, 2019	Pre-COVID state characteristic	Normalize	1/1/20 - 7/31/22	log(cumulative infections per capita)	comorbidities	Kaiser Family Foundation
**ICU beds**	ICU beds per capita, 2019	Pre-COVID state characteristic	Normalize	1/1/20 - 7/31/22	log(cumulative infections per capita)	comorbidities	Kaiser Family Foundation

**Physicians**	Physicians per capita, 2019	Pre-COVID state characteristic	Normalize	1/1/20 - 7/31/22	log(cumulative infections per capita)	comorbidities	Kaiser Family Foundation
**Healthcare workers**	Healthcare workers per capita, 2019	Pre-COVID state characteristic	Normalize	1/1/20 - 7/31/22	log(cumulative infections per capita)	comorbidities	Kaiser Family Foundation
**Health insurance**	Proportion of the population who are uninsured, 2019	Pre-COVID state characteristic	Normalize	1/1/20 - 7/31/22	log(cumulative infections per capita)	comorbidities	US Census: Smaller Area Health Insurance Estimates
**Governor party affiliation**	Governor's political party affiliation (Republican/Democrat), 2019	Pre-COVID state characteristic	None	1/1/20 - 7/31/22	log(cumulative infections per capita)	comorbidities	Harvard Dataverse
**Vote share (2020)**	% of the vote that went for the Republican presidential candidate in 2020	Pre-COVID state characteristic	Normalize	1/1/20 - 7/31/22	log(cumulative infections per capita)	comorbidities	Harvard Dataverse
**Interpersonal trust**	Most people can be trusted	Pre-COVID state characteristic	Normalize	1/1/20 - 7/31/22	log(cumulative infections per capita)	comorbidities	2016/2018 Cooperative Congressional Election Survey (CCES)
**Trust in science**	A lot or moderate amount of trust in the scientific community	Pre-COVID state characteristic	Normalize	1/1/20 - 7/31/22	log(cumulative infections per capita)	comorbidities	2018 Cooperative Congressional Election Survey (CCES)
**Trust in government**	Always or almost always trust the federal government to do what is right	Pre-COVID state characteristic	Normalize	1/1/20 - 7/31/22	log(cumulative infections per capita)	comorbidities	2016/2018 Cooperative Congressional Election Survey (CCES)
**Paid family/sick leave**	Existence of state-funded paid family or medical leave (Neither/One/Both), 2019	Pre-COVID state characteristic	None	1/1/20 - 7/31/22	log(cumulative infections per capita)	comorbidities	Kaiser Family Foundation
**Education**	Average years of education, 2019	Pre-COVID state characteristic	Normalize	1/1/20 - 7/31/22	log(cumulative infections per capita)	comorbidities	American Community Survey

**Mandate propensity**	First principal component of a PCA of the following variables: mask mandates, stay-at-home orders, restaurant closures, bar closures, gathering restrictions, primary school closures, higher education closures, and gym/pool/leisure closures.	COVID policy	None	4/1/20 - 6/1/21	mean(log(daily infections per capita))	comorbidities	*Constructed*
**Mask mandates**	% of days during the analysis time period with a mask mandate in effect	COVID policy	None	4/1/20 - 6/1/21	mean(log(daily infections per capita))	comorbidities + leave-one-out mandate pca	COVID19StatePolicy.org; Christopher Adolph, Kenya Amano, Bree Bang-Jensen, Nancy Fullman, John Wilkerson. 2021. "Pandemic Politics: Timing State-Level Social Distancing Responses to COVID-19." Journal of Health Politics, Policy and Law; Christopher Adolph, Kenya Amano, Bree Bang-Jensen, Nancy Fullman, Beatrice Magistro, Grace Reinke, Rachel Castellano, Megan Erickson, John Wilkerson. 2021. "The Pandemic Policy U-Turn: Partisanship, Public Health, and Race in Decisions to Ease COVID-19 Social Distancing Policies in the United

							States." Perspectives on Politics.
**Stay at home orders**	% of days during the analysis time period with a stay-at-home order in effect	COVID policy	None	4/1/20 - 6/1/21	mean(log(daily infections per capita))	comorbidities + leave-one-out mandate pca	
**Restaurant closures**	% of days during the analysis time period with restaurants mandated shut	COVID policy	None	4/1/20 - 6/1/21	mean(log(daily infections per capita))	comorbidities + leave-one-out mandate pca	
**Bar closures**	% of days during the analysis time period with bars mandated shut	COVID policy	None	4/1/20 - 6/1/21	mean(log(daily infections per capita))	comorbidities + leave-one-out mandate pca	
**Gathering restrictions**	% of days during the analysis time period with restrictions in place for indoor gatherings of 50 people or more and outdoor gatherings of 100 people or more	COVID policy	None	4/1/20 - 6/1/21	mean(log(daily infections per capita))	comorbidities + leave-one-out mandate pca	

**Primary school closures**	% of days during the analysis time period with primary schools mandated shut for in-person activities	COVID policy	None	4/1/20 - 6/1/21	mean(log(daily infections per capita))	comorbidities + leave-one-out mandate pca	
**Higher education closures**	% of days during the analysis time period with higher education institutions mandated shut for in-person activities	COVID policy	None	4/1/20 - 6/1/21	mean(log(daily infections per capita))	comorbidities + leave-one-out mandate pca	
**Gym/pool/leisure closures**	% of days during the analysis time period with gyms, pools, and other leisure destinations mandated shut	COVID policy	None	4/1/20 - 6/1/21	mean(log(daily infections per capita))	comorbidities + leave-one-out mandate pca	
**Vaccine mandates (school employees)**	History of COVID vaccine mandate for school employees (Yes/No)	COVID policy	None	3/15/21 - 07/31/22	log(cumulative infections per capita)	comorbidities + leave-one-out mandate pca	Kaiser Family Foundation
**Vaccine mandates (state employees)**	History of COVID vaccine mandate for state employees (Yes/No/Partial)	COVID policy	None	3/15/21 - 07/31/22	log(cumulative infections per capita)	comorbidities + leave-one-out mandate pca	Kaiser Family Foundation
**Mask use**	Average proportion of the population who always wear a mask when leaving home	COVID behavior	Mean of daily measure	4/1/20 - 07/31/22	mean(log(daily infections per capita))	comorbidities	IHME COVID-19 database
**Mobility**	Average daily relative change in mobility from pre-pandemic baseline	COVID behavior	Mean of daily meaure	4/1/20 - 07/31/22	mean(log(daily infections per capita))	comorbidities	Google Global COVID-19 Community Mobility Report; Apple Inc. COVID-19 Mobility Trends Report; United States Mobility Changes in Response to COVID-19 2020 - Descarte Labs; United States SafeGraph Social Distancing Metrics 2020.

**Vaccine uptake**	Age-standardized fully vaccinated person-days per total person-days, where fully vaccinated is defined as receiving 2 doses of a two-dose regimen or a single dose of a one-dose regimen	COVID behavior	None	3/15/21 - 07/31/22	mean(log(daily infections per capita))	comorbidities	IHME COVID-19 database

3.2 Infection model inputs

Description of all dependent and independent variables explored in the infection regression analysis, including mathematical transformations, relevant time period, control variables, and data sources.

Variable name	Definition	Category	Transformation	Analysis Time Period	Dependent variable transformation	Controls	Source
**Infections**	Cumulative infections per capita	Dependent variable	Log	NA	NA	NA	IHME COVID estimate, based on case reporting from JHU
**Population density**	Proportion of the population living in an area with at least 500 ppl per sq km, 2019	Control	Normalize	NA	NA	NA	US Census Bureau; TIGERLS
**Hispanic**	Proportion of the population identifying as Hispanic	Race/ Ethnicity	Normalize	1/1/20 - 12/15/21	log(cumulative infections per capita)	population density	US Census Bureau
**Non-Hispanic Asian**	Proportion of the population identifying as non-Hispanic and Asian or Pacific Islander	Race/ Ethnicity	Normalize	1/1/20 - 12/15/21	log(cumulative infections per capita)	population density	US Census Bureau
**Non-Hispanic Black**	Proportion of the population identifying as non-Hispanic and Black	Race/ Ethnicity	Normalize	1/1/20 - 12/15/21	log(cumulative infections per capita)	population density	US Census Bureau
**Non-Hispanic White**	Proportion of the population identifying as non-Hispanic and White	Race/ Ethnicity	Normalize	1/1/20 - 12/15/21	log(cumulative infections per capita)	population density	US Census Bureau
**Non-Hispanic American Indian**	Proportion of the population identifying as non-Hispanic and American Indian or Alaska Native	Race/ Ethnicity	Normalize	1/1/20 - 12/15/21	log(cumulative infections per capita)	population density	US Census Bureau
**Poverty**	% of the population living below the poverty line, 2019	Pre-COVID state characteristic	Normalize	1/1/20 - 12/15/21	log(cumulative infections per capita)	population density	GBD 2019
**Income inequality**	Gini coefficient, 2019	Pre-COVID state characteristic	Normalize	1/1/20 - 12/15/21	log(cumulative infections per capita)	population density	US Census Bureau

**Health access and quality**	Health access and quality index (HAQI), 2019	Pre-COVID state characteristic	Normalize	1/1/20 - 12/15/21	log(cumulative infections per capita)	population density	GBD 2019
**Public health FTEs**	Public health full-time equivalent (FTE) hours per capita, 2019	Pre-COVID state characteristic	Normalize	1/1/20 - 12/15/21	log(cumulative infections per capita)	population density	US Census: State Government Employment & Payroll
**Governor party affiliation**	Governor's political party affiliation (Republican/Democrat), 2019	Pre-COVID state characteristic	None	1/1/20 - 12/15/21	log(cumulative infections per capita)	population density	Harvard Dataverse
**Vote share (2020)**	% of the vote that went for the Republican presidential candidate in 2020	Pre-COVID state characteristic	Normalize	1/1/20 - 12/15/21	log(cumulative infections per capita)	population density	Harvard Dataverse
**Interpersonal trust**	Most people can be trusted	Pre-COVID state characteristic	Normalize	1/1/20 - 12/15/21	log(cumulative infections per capita)	population density	2016/2018 Cooperative Congressional Election Survey (CCES)
**Trust in science**	A lot or moderate amount of trust in the scientific community	Pre-COVID state characteristic	Normalize	1/1/20 - 12/15/21	log(cumulative infections per capita)	population density	2018 Cooperative Congressional Election Survey (CCES)
**Trust in government**	Always or almost always trust the federal government to do what is right	Pre-COVID state characteristic	Normalize	1/1/20 - 12/15/21	log(cumulative infections per capita)	population density	2016/2018 Cooperative Congressional Election Survey (CCES)
**Paid family/sick leave**	Existence of state-funded paid family or medical leave (Neither/One/Both), 2019	Pre-COVID state characteristic	None	1/1/20 - 12/15/21	log(cumulative infections per capita)	population density	Kaiser Family Foundation
**Education**	Average years of education, 2019	Pre-COVID state characteristic	Normalize	1/1/20 - 12/15/21	log(cumulative infections per capita)	population density	American Community Survey
**Mandate propensity**	First principal component of a PCA of the following variables: mask mandates, stay-at-home orders, restaurant closures, bar closures, gathering restrictions, primary school closures, higher education closures, and gym/pool/leisure closures.	COVID policy	None	4/1/20 - 6/1/21	mean(log(daily infections per capita))	population density	*Constructed*
**Mask mandates**	% of days during the analysis time period with a mask mandate in effect	COVID policy	None	4/1/20 - 6/1/21	mean(log(daily infections per capita))	proportion_500ppl_km + leave-one-out mandate pca	COVID19StatePolicy.org; Christopher Adolph, Kenya Amano, Bree Bang-Jensen, Nancy Fullman, John

							Wilkerson. 2021. "Pandemic Politics: Timing State-Level Social Distancing Responses to COVID-19." Journal of Health Politics, Policy and Law; Christopher Adolph, Kenya Amano, Bree Bang-Jensen, Nancy Fullman, Beatrice Magistro, Grace Reinke, Rachel Castellano, Megan Erickson, John Wilkerson. 2021. "The Pandemic Policy U-Turn: Partisanship, Public Health, and Race in Decisions to Ease COVID-19 Social Distancing Policies in the United States." Perspectives on Politics.
**Stay at home orders**	% of days during the analysis time period with a stay-at-home order in effect	COVID policy	None	4/1/20 - 6/1/21	mean(log(daily infections per capita))	proportion_500ppl_km + leave-one-out mandate pca	
**Restaurant closures**	% of days during the analysis time period with restaurants mandated shut	COVID policy	None	4/1/20 - 6/1/21	mean(log(daily infections per capita))	proportion_500ppl_km + leave-one-out mandate pca	
**Bar closures**	% of days during the analysis time period with bars mandated shut	COVID policy	None	4/1/20 - 6/1/21	mean(log(daily infections per capita))	proportion_500ppl_km + leave-one-out mandate pca	
**Gathering restrictions**	% of days during the analysis time period with restrictions in place for indoor gatherings of 50 people or more and outdoor gatherings of 100 people or more	COVID policy	None	4/1/20 - 6/1/21	mean(log(daily infections per capita))	proportion_500ppl_km + leave-one-out mandate pca	
**Primary school closures**	% of days during the analysis time period with primary schools mandated shut for in-person activities	COVID policy	None	4/1/20 - 6/1/21	mean(log(daily infections per capita))	proportion_500ppl_km + leave-one-out mandate pca	

**Higher education closures**	% of days during the analysis time period with higher education institutions mandated shut for in-person activities	COVID policy	None	4/1/20 - 6/1/21	mean(log(daily infections per capita))	proportion_500ppl_km + leave-one-out mandate pca	
**Gym/pool/leisure closures**	% of days during the analysis time period with gyms, pools, and other leisure destinations mandated shut	COVID policy	None	4/1/20 - 6/1/21	mean(log(daily infections per capita))	proportion_500ppl_km + leave-one-out mandate pca	
**Vaccine mandates (school employees)**	History of COVID vaccine mandate for school employees (Yes/No)	COVID policy	None	3/15/21 - 12/15/21	log(cumulative infections per capita)	proportion_500ppl_km + leave-one-out mandate pca	Kaiser Family Foundation
**Vaccine mandates (state employees)**	History of COVID vaccine mandate for state employees (Yes/No/Partial)	COVID policy	None	3/15/21 - 12/15/21	log(cumulative infections per capita)	proportion_500ppl_km + leave-one-out mandate pca	Kaiser Family Foundation
**Mask use**	Average proportion of the population who always wear a mask when leaving home	COVID behavior	Mean of daily measure	4/1/20 - 12/15/21	mean(log(daily infections per capita))	population density	IHME COVID-19 database
**Mobility**	Average daily relative change in mobility from pre-pandemic baseline	COVID behavior	Mean of daily measure	4/1/20 - 12/15/21	mean(log(daily infections per capita))	population density	Google Global COVID-19 Community Mobility Report; Apple Inc. COVID-19 Mobility Trends Report; United States Mobility Changes in Response to COVID-19 2020 - Descarte Labs; United States SafeGraph Social Distancing Metrics 2020.
**Vaccine uptake**	Fully vaccinated person-days per total person-days, where fully vaccinated is defined as receiving 2 doses of a two-dose regimen or a single dose of a one-dose regimen	COVID behavior	None	3/15/21 - 12/15/21	mean(log(daily infections per capita))	population density	IHME COVID-19 database

3.3 GDP model inputs

Description of all dependent and independent variables explored in the GDP regression analysis, including mathematical transformations, relevant time period, control variables, and data sources.

Variable name	Definition	Category	Transformation	Analysis Time Period	Dependent variable transformation	Controls	Source
**GDP**	Sector-standardised GDP relative to 2019	Dependent variable	None	NA	NA	NA	Bureau of Economic Analysis (BEA)
**Education**	Average years of education, 2019	Control	Normalize	NA	NA	NA	GBD 2019
**Population 65+**	Proportion of the population aged 65 and up, 2019	Control	Normalize	NA	NA	NA	GBD 2019
**Population <20**	Proportion of the population under 20 years old, 2019	Control	Normalize	NA	NA	NA	GBD 2019
**Unemployment insurance, benefit length**	Average length (in weeks) of unemployment insurance benefit, 2019	Control	Normalize	NA	NA	NA	US Department of Labor, Center on Budget and Policy Priorities, Missouri Economic Research and Information Center

**Unemployment insurance, benefit amount**	Average price-adjusted unemployment insurance benefit amount (in dollars), 2019	Control	Normalize	NA	NA	NA	US Department of Labor, Center on Budget and Policy Priorities, Missouri Economic Research and Information Center
**Poverty**	% of the population living below the poverty line, 2019	Pre-COVID state characteristic	Normalize	1/1/20 - 6/30/22	log(cumulative infections per capita)	education + population over 65 + population under 20 + UI benefit length + UI benefit amount	GBD 2019
**Income inequality**	Gini coefficient, 2019	Pre-COVID state characteristic	Normalize	1/1/20 - 6/30/22	log(cumulative infections per capita)	education + population over 65 + population under 20 + UI benefit length + UI benefit amount	US Census Bureau
**Governor party affiliation**	Governor's political party affiliation (Republican/Democrat), 2019	Pre-COVID state characteristic	None	1/1/20 - 6/30/22	log(cumulative infections per capita)	education + population over 65 + population under 20 + UI benefit length + UI benefit amount	Harvard Dataverse

**Vote share (2020)**	% of the vote that went for the Republican presidential candidate in 2020	Pre-COVID state characteristic	Normalize	1/1/20 - 6/30/22	log(cumulative infections per capita)	education + population over 65 + population under 20 + UI benefit length + UI benefit amount	Harvard Dataverse
**Interpersonal trust**	Most people can be trusted	Pre-COVID state characteristic	Normalize	1/1/20 - 6/30/22	log(cumulative infections per capita)	education + population over 65 + population under 20 + UI benefit length + UI benefit amount	2016/2018 Cooperative Congressional Election Survey (CCES)
**Trust in science**	A lot or moderate amount of trust in the scientific community	Pre-COVID state characteristic	Normalize	1/1/20 - 6/30/22	log(cumulative infections per capita)	education + population over 65 + population under 20 + UI benefit length + UI benefit amount	2018 Cooperative Congressional Election Survey (CCES)
**Trust in government**	Always or almost always trust the federal government to do what is right	Pre-COVID state characteristic	Normalize	1/1/20 - 6/30/22	log(cumulative infections per capita)	education + population over 65 + population under 20 + UI benefit length + UI benefit amount	2016/2018 Cooperative Congressional Election Survey (CCES)

**Paid family/sick leave**	Existence of state-funded paid family or medical leave (Neither/One/Both), 2019	Pre-COVID state characteristic	None	1/1/20 - 6/30/22	log(cumulative infections per capita)	education + population over 65 + population under 20 + UI benefit length + UI benefit amount	Kaiser Family Foundation
**Mandate propensity**	First principal component of a PCA of the following variables: mask mandates, stay-at-home orders, restaurant closures, bar closures, gathering restrictions, primary school closures, higher education closures, and gym/pool/leisure closures.	COVID policy	None	4/1/20 - 6/1/21	mean(log(daily infections per capita))	population density + comorbidities	*Constructed*
**Mask mandates**	% of days during the analysis time period with a mask mandate in effect	COVID policy	None	4/1/20 - 6/1/21	mean(log(daily infections per capita))	education + population over 65 + population under 20 + UI benefit length + UI benefit amount + leave-one-out mandate PCA	COVID19StatePolicy.org; Christopher Adolph, Kenya Amano, Bree Bang-Jensen, Nancy Fullman, John Wilkerson. 2021. "Pandemic Politics: Timing State-Level Social Distancing Responses to COVID-

**Stay at home orders**	% of days during the analysis time period with a stay-at-home order in effect	COVID policy	None	4/1/20 - 6/1/21	mean(log(daily infections per capita))	education + population over 65 + population under 20 + UI benefit length + UI benefit amount + leave-one-out mandate PCA	19." Journal of Health Politics, Policy and Law; Christopher Adolph, Kenya Amano, Bree Bang-Jensen, Nancy Fullman, Beatrice Magistro, Grace Reinke, Rachel Castellano, Megan Erickson, John Wilkerson. 2021. "The Pandemic Policy U-Turn: Partisanship, Public Health, and Race in Decisions to Ease COVID-19 Social Distancing Policies in the United States." Perspectives on Politics.
**Restaurant closures**	% of days during the analysis time period with restaurants mandated shut	COVID policy	None	4/1/20 - 6/1/21	mean(log(daily infections per capita))	education + population over 65 + population under 20 + UI benefit length + UI benefit amount + leave-one-out mandate PCA	
**Bar closures**	% of days during the analysis time period with bars mandated shut	COVID policy	None	4/1/20 - 6/1/21	mean(log(daily infections per capita))	education + population over 65 + population under 20 + UI benefit length + UI benefit amount + leave-one-out mandate PCA	
**Gathering restrictions**	% of days during the analysis time period with restrictions in place for indoor gatherings of 50 people or more and outdoor gatherings of 100 people or more	COVID policy	None	4/1/20 - 6/1/21	mean(log(daily infections per capita))	education + population over 65 + population under 20 + UI benefit length + UI benefit amount +	

						leave-one-out mandate PCA	
**Primary school closures**	% of days during the analysis time period with primary schools mandated shut for in-person activities	COVID policy	None	4/1/20 - 6/1/21	mean(log(daily infections per capita))	education + population over 65 + population under 20 + UI benefit length + UI benefit amount + leave-one-out mandate PCA	
**Higher education closures**	% of days during the analysis time period with higher education institutions mandated shut for in-person activities	COVID policy	None	4/1/20 - 6/1/21	mean(log(daily infections per capita))	education + population over 65 + population under 20 + UI benefit length + UI benefit amount + leave-one-out mandate PCA	
**Gym/pool/leisure closures**	% of days during the analysis time period with gyms, pools, and other leisure destinations mandated shut	COVID policy	None	4/1/20 - 6/1/21	mean(log(daily infections per capita))	education + population over 65 + population under 20 + UI benefit length + UI benefit amount + leave-one-out mandate PCA	

**Federal unemployment insurance benefit duration**	Average length (in weeks) of unemployment insurance benefit, 2019	COVID policy	None	4/1/20 - 06/30/22	mean(log(daily infections per capita))	education + population over 65 + population under 20 + UI benefit length + UI benefit amount	US Department of Labor, Center on Budget and Policy Priorities, Missouri Economic Research and Information Center
**Federal unemployment insurance benefit amount**	Average price-adjusted unemployment insurance benefit amount (in dollars), 2019	COVID policy	None	4/1/20 - 06/30/22	mean(log(daily infections per capita))	education + population over 65 + population under 20 + UI benefit length + UI benefit amount	US Department of Labor, Center on Budget and Policy Priorities, Missouri Economic Research and Information Center
**Vaccine mandates (school employees)**	History of COVID vaccine mandate for school employees (Yes/No)	COVID policy	None	3/15/21 - 06/30/22	log(cumulative infections per capita)	education + population over 65 + population under 20 + UI benefit length + UI benefit amount	Kaiser Family Foundation
**Vaccine mandates (state employees)**	History of COVID vaccine mandate for state employees (Yes/No/Partial)	COVID policy	None	3/15/21 - 06/30/22	log(cumulative infections per capita)	education + population over 65 + population under 20 + UI benefit length + UI benefit amount	Kaiser Family Foundation

**Mask use**	Average proportion of the population who always wear a mask when leaving home	COVID behavior	None	4/1/20 - 06/30/22	mean(log(daily infections per capita))	education + population over 65 + population under 20 + UI benefit length + UI benefit amount	IHME COVID-19 database
**Mobility**	Average daily relative change in mobility from pre-pandemic baseline	COVID behavior	None	4/1/20 - 06/30/22	mean(log(daily infections per capita))	education + population over 65 + population under 20 + UI benefit length + UI benefit amount	Google Global COVID-19 Community Mobility Report; Apple Inc. COVID-19 Mobility Trends Report; United States Mobility Changes in Response to COVID-19 2020 - Descarte Labs; United States SafeGraph Social Distancing Metrics 2020.
**Vaccine uptake**	Fully vaccinated person-days per total person-days, where fully vaccinated is defined as receiving 2 doses of a two-dose regimen or a single dose of a one-dose regimen	COVID behavior	None	3/15/21 - 06/30/22	mean(log(daily infections per capita))	education + population over 65 + population under 20 + UI benefit length + UI benefit amount	IHME COVID-19 database

**Infections**	Cumulative infections per capita	COVID outcomes	Log, Normalize	4/1/20 - 12/15/21	mean(log(daily infections per capita))	education + population over 65 + population under 20 + UI benefit length + UI benefit amount	IHME COVID-19 database
**Deaths**	Cumulative deaths per capita	COVID outcomes	Log, Normalize	4/1/20 - 06/30/22	mean(log(daily infections per capita))	education + population over 65 + population under 20 + UI benefit length + UI benefit amount	IHME COVID-19 database
**IFR**	Age-standardised Infection-fatality rate	COVID outcomes	Normalize	4/1/20 - 12/15/21	mean(log(daily infections per capita))	education + population over 65 + population under 20 + UI benefit length + UI benefit amount	IHME COVID-19 database
**Hospitalisations**	Cumulative hospitalisations per capita	COVID outcomes	Normalize	4/1/20 - 12/15/21	mean(log(daily infections per capita))	education + population over 65 + population under 20 + UI benefit length + UI benefit amount	IHME COVID-19 database

3.4 Employment model inputs

Description of all dependent and independent variables explored in the employment regression analysis, including mathematical transformations, relevant time period, control variables, and data sources.

Variable name	Definition	Category	Transformation	Analysis Time Period	Dependent variable transformation	Controls	Source
**Employment**	Sector-standardised employment relative to 2019	Dependent variable	None	NA	NA	NA	Bureau of Labor Statistics (accessed via Federal Reserve Economic Data)
**Education**	Average years of education, 2019	Control	Normalize	NA	NA	NA	GBD 2019
**Population 65+**	Proportion of the population aged 65 and up, 2019	Control	Normalize	NA	NA	NA	GBD 2019
**Population <20**	Proportion of the population under 20 years old, 2019	Control	Normalize	NA	NA	NA	GBD 2019
**Unemployment insurance, benefit length**	Average length (in weeks) of unemployment insurance benefit, 2019	Control	Normalize	NA	NA	NA	US Department of Labor, Center on Budget and Policy Priorities, Missouri Economic Research and Information Center
**Unemployment insurance, benefit amount**	Average price-adjusted unemployment insurance benefit amount (in dollars), 2019	Control	Normalize	NA	NA	NA	US Department of Labor, Center on Budget and Policy Priorities, Missouri Economic Research and Information Center

**Poverty**	% of the population living below the poverty line, 2019	Pre-COVID state characteristic	Normalize	1/1/20 - 7/31/22	log(cumulative infections per capita)	education + population over 65 + population under 20 + UI benefit length + UI benefit amount	GBD 2019
**Income inequality**	Gini coefficient, 2019	Pre-COVID state characteristic	Normalize	1/1/20 - 7/31/22	log(cumulative infections per capita)	education + population over 65 + population under 20 + UI benefit length + UI benefit amount	US Census Bureau
**Governor party affiliation**	Governor's political party affiliation (Republican/Democrat), 2019	Pre-COVID state characteristic	None	1/1/20 - 7/31/22	log(cumulative infections per capita)	education + population over 65 + population under 20 + UI benefit length + UI benefit amount	Harvard Dataverse
**Vote share (2020)**	% of the vote that went for the Republican presidential candidate in 2020	Pre-COVID state characteristic	Normalize	1/1/20 - 7/31/22	log(cumulative infections per capita)	education + population over 65 + population under 20 + UI benefit length + UI benefit amount	Harvard Dataverse
**Interpersonal trust**	Most people can be trusted	Pre-COVID state characteristic	Normalize	1/1/20 - 7/31/22	log(cumulative infections per capita)	education + population over 65 + population under 20 + UI benefit length	2016/2018 Cooperative Congressional Election Survey (CCES)

						+ UI benefit amount	
**Trust in science**	A lot or moderate amount of trust in the scientific community	Pre-COVID state characteristic	Normalize	1/1/20 - 7/31/22	log(cumulative infections per capita)	education + population over 65 + population under 20 + UI benefit length + UI benefit amount	2018 Cooperative Congressional Election Survey (CCES)
**Trust in government**	Always or almost always trust the federal government to do what is right	Pre-COVID state characteristic	Normalize	1/1/20 - 7/31/22	log(cumulative infections per capita)	education + population over 65 + population under 20 + UI benefit length + UI benefit amount	2016/2018 Cooperative Congressional Election Survey (CCES)
**Paid family/sick leave**	Existence of state-funded paid family or medical leave (Neither/One/Both), 2019	Pre-COVID state characteristic	None	1/1/20 - 7/31/22	log(cumulative infections per capita)	education + population over 65 + population under 20 + UI benefit length + UI benefit amount	Kaiser Family Foundation

**Mandate propensity**	First principal component of a PCA of the following variables: mask mandates, stay-at-home orders, restaurant closures, bar closures, gathering restrictions, primary school closures, higher education closures, and gym/pool/leisure closures.	COVID policy	None	4/1/20 - 6/1/21	mean(log(daily infections per capita))	population density + comorbidities	*Constructed*
**Mask mandates**	% of days during the analysis time period with a mask mandate in effect	COVID policy	None	4/1/20 - 6/1/21	mean(log(daily infections per capita))	education + population over 65 + population under 20 + UI benefit length + UI benefit amount + leave-one-out mandate PCA	COVID19StatePolicy.org; Christopher Adolph, Kenya Amano, Bree Bang-Jensen, Nancy Fullman, John Wilkerson. 2021. "Pandemic Politics: Timing State-Level Social Distancing Responses to COVID-19." Journal of Health Politics, Policy and Law; Christopher Adolph, Kenya Amano, Bree Bang-Jensen, Nancy Fullman, Beatrice Magistro, Grace Reinke, Rachel Castellano, Megan Erickson, John Wilkerson. 2021. "The
**Stay at home orders**	% of days during the analysis time period with a stay-at-home order in effect	COVID policy	None	4/1/20 - 6/1/21	mean(log(daily infections per capita))	education + population over 65 + population under 20 + UI benefit length + UI benefit amount + leave-one-out mandate PCA	

**Restaurant closures**	% of days during the analysis time period with restaurants mandated shut	COVID policy	None	4/1/20 - 6/1/21	mean(log(daily infections per capita))	education + population over 65 + population under 20 + UI benefit length + UI benefit amount + leave-one-out mandate PCA	Pandemic Policy U-Turn: Partisanship, Public Health, and Race in Decisions to Ease COVID-19 Social Distancing Policies in the United States." Perspectives on Politics.
**Bar closures**	% of days during the analysis time period with bars mandated shut	COVID policy	None	4/1/20 - 6/1/21	mean(log(daily infections per capita))	education + population over 65 + population under 20 + UI benefit length + UI benefit amount + leave-one-out mandate PCA	
**Gathering restrictions**	% of days during the analysis time period with restrictions in place for indoor gatherings of 50 people or more and outdoor gatherings of 100 people or more	COVID policy	None	4/1/20 - 6/1/21	mean(log(daily infections per capita))	education + population over 65 + population under 20 + UI benefit length + UI benefit amount + leave-one-out mandate PCA	
**Primary school closures**	% of days during the analysis time period with primary schools mandated shut for in-person activities	COVID policy	None	4/1/20 - 6/1/21	mean(log(daily infections per capita))	education + population over 65 + population under 20 + UI benefit length + UI benefit amount +	

						leave-one-out mandate PCA	
**Higher education closures**	% of days during the analysis time period with higher education institutions mandated shut for in-person activities	COVID policy	None	4/1/20 - 6/1/21	mean(log(daily infections per capita))	education + population over 65 + population under 20 + UI benefit length + UI benefit amount + leave-one-out mandate PCA	
**Gym/pool/leisure closures**	% of days during the analysis time period with gyms, pools, and other leisure destinations mandated shut	COVID policy	None	4/1/20 - 6/1/21	mean(log(daily infections per capita))	education + population over 65 + population under 20 + UI benefit length + UI benefit amount + leave-one-out mandate PCA	
**Federal unemployment insurance benefit duration**	Average length (in weeks) of unemployment insurance benefit, 2019	COVID policy	None	4/1/20 - 7/31/22	mean(log(daily infections per capita))	education + population over 65 + population under 20 + UI benefit length + UI benefit amount	US Department of Labor, Center on Budget and Policy Priorities, Missouri Economic Research and Information Center

**Federal unemployment insurance benefit amount**	Average price-adjusted unemployment insurance benefit amount (in dollars), 2019	COVID policy	None	4/1/20 - 7/31/22	mean(log(daily infections per capita))	education + population over 65 + population under 20 + UI benefit length + UI benefit amount	US Department of Labor, Center on Budget and Policy Priorities, Missouri Economic Research and Information Center
**Vaccine mandates (school employees)**	History of COVID vaccine mandate for school employees (Yes/No)	COVID policy	None	3/15/21 - 07/31/22	log(cumulative infections per capita)	education + population over 65 + population under 20 + UI benefit length + UI benefit amount	Kaiser Family Foundation
**Vaccine mandates (state employees)**	History of COVID vaccine mandate for state employees (Yes/No/Partial)	COVID policy	None	3/15/21 - 7/31/22	log(cumulative infections per capita)	education + population over 65 + population under 20 + UI benefit length + UI benefit amount	Kaiser Family Foundation
**Mask use**	Average proportion of the population who always wear a mask when leaving home	COVID behavior	None	4/1/20 - 7/31/22	mean(log(daily infections per capita))	education + population over 65 + population under 20 + UI benefit length + UI benefit amount	IHME COVID-19 database

**Mobility**	Average daily relative change in mobility from pre-pandemic baseline	COVID behavior	None	4/1/20 - 7/31/22	mean(log(daily infections per capita))	education + population over 65 + population under 20 + UI benefit length + UI benefit amount	Google Global COVID-19 Community Mobility Report; Apple Inc. COVID-19 Mobility Trends Report; United States Mobility Changes in Response to COVID-19 2020 - Descarte Labs; United States SafeGraph Social Distancing Metrics 2020.
**Vaccine uptake**	Fully vaccinated person-days per total person-days, where fully vaccinated is defined as receiving 2 doses of a two-dose regimen or a single dose of a one-dose regimen	COVID behavior	None	3/15/21 - 07/31/22	mean(log(daily infections per capita))	education + population over 65 + population under 20 + UI benefit length + UI benefit amount	IHME COVID-19 database
**Infections**	Cumulative infections per capita	COVID outcomes	Log, Normalize	4/1/20 - 12/15/21	mean(log(daily infections per capita))	education + population over 65 + population under 20 + UI benefit length + UI benefit amount	IHME COVID-19 database
**Deaths**	Cumulative deaths per capita	COVID outcomes	Log, Normalize	4/1/20 - 07/31/22	mean(log(daily infections per capita))	education + population over 65 + population under 20 + UI benefit length	IHME COVID-19 database

						+ UI benefit amount	
**IFR**	Age-standardised Infection-fatality rate	COVID outcomes	Normalize	4/1/20 - 12/15/21	mean(log(daily infections per capita))	education + population over 65 + population under 20 + UI benefit length + UI benefit amount	IHME COVID-19 database
**Hospitalisations**	Cumulative hospitalisations per capita	COVID outcomes	Normalize	4/1/20 - 12/15/21	mean(log(daily infections per capita))	education + population over 65 + population under 20 + UI benefit length + UI benefit amount	IHME COVID-19 database

3.5 Education model inputs

Description of all dependent and independent variables explored in the education regression analysis, including mathematical transformations, relevant time period, control variables, and data sources.

Variable name	Definition	Category	Transformation	Analysis Time Period	Dependent variable transformation	Controls	Source
**Education**	Change in mean test score from 2019 to 2022 for 4th and 8th grade math and reading	Dependent variable	None	NA	NA	NA	National Center for Education Statistics
**Poverty**	% of the population living below the poverty line, 2019	Pre-COVID state characteristic	Normalize	1/1/20 - 2/15/22	log(cumulative infections per capita)	NA	GBD 2019
**Income inequality**	Gini coefficient, 2019	Pre-COVID state characteristic	Normalize	1/1/20 - 2/15/22	log(cumulative infections per capita)	NA	US Census Bureau
**Governor party affiliation**	Governor's political party affiliation (Republican/Democrat), 2019	Pre-COVID state characteristic	None	1/1/20 - 2/15/22	log(cumulative infections per capita)	NA	Harvard Dataverse
**Vote share (2020)**	% of the vote that went for the Republican presidential candidate in 2020	Pre-COVID state characteristic	Normalize	1/1/20 - 2/15/22	log(cumulative infections per capita)	NA	Harvard Dataverse
**Interpersonal trust**	Most people can be trusted	Pre-COVID state characteristic	Normalize	1/1/20 - 2/15/22	log(cumulative infections per capita)	NA	2016/2018 Cooperative Congressional Election Survey (CCES)
**Trust in science**	A lot or moderate amount of trust in the scientific community	Pre-COVID state characteristic	Normalize	1/1/20 - 2/15/22	log(cumulative infections per capita)	NA	2018 Cooperative Congressional Election Survey (CCES)

**Trust in government**	Always or almost always trust the federal government to do what is right	Pre-COVID state characteristic	Normalize	1/1/20 - 2/15/22	log(cumulative infections per capita)	NA	2016/2018 Cooperative Congressional Election Survey (CCES)
**Mandate propensity**	First principal component of a PCA of the following variables: mask mandates, stay-at-home orders, restaurant closures, bar closures, gathering restrictions, primary school closures, higher education closures, and gym/pool/leisure closures.	COVID policy	None	4/1/20 - 6/1/21	mean(log(daily infections per capita))	population density + comorbidities	*Constructed*
**Mask mandates**	% of days during the analysis time period with a mask mandate in effect	COVID policy	None	4/1/20 - 2/15/22	mean(log(daily infections per capita))	leave-one-out mandate PCA	COVID19StatePolicy.org; Christopher Adolph, Kenya Amano, Bree Bang-Jensen, Nancy Fullman, John Wilkerson. 2021. "Pandemic Politics: Timing State-Level Social Distancing Responses to COVID-19." Journal of Health Politics, Policy and Law; Christopher Adolph, Kenya Amano, Bree Bang-Jensen, Nancy Fullman, Beatrice
**Stay at home orders**	% of days during the analysis time period with a stay-at-home order in effect	COVID policy	None	4/1/20 - 2/15/22	mean(log(daily infections per capita))	leave-one-out mandate PCA	
**Gathering restrictions**	% of days during the analysis time period with restrictions in place for indoor gatherings of 50 people or more and outdoor gatherings of 100 people or more	COVID policy	None	4/1/20 - 2/15/22	mean(log(daily infections per capita))	leave-one-out mandate PCA	

**Primary school closures**	% of days during the analysis time period with primary schools mandated shut for in-person activities	COVID policy	None	4/1/20 - 2/15/22	mean(log(daily infections per capita))	leave-one-out mandate PCA	Magistro, Grace Reinke, Rachel Castellano, Megan Erickson, John Wilkerson. 2021. "The Pandemic Policy U-Turn: Partisanship, Public Health, and Race in Decisions to Ease COVID-19 Social Distancing Policies in the United States." Perspectives on Politics.
**Higher education closures**	% of days during the analysis time period with higher education institutions mandated shut for in-person activities	COVID policy	None	4/1/20 - 2/15/22	mean(log(daily infections per capita))	leave-one-out mandate PCA	
**Vaccine mandates (school employees)**	History of COVID vaccine mandate for school employees (Yes/No)	COVID policy	None	1/1/20 - 2/15/22	log(cumulative infections per capita)	leave-one-out mandate PCA	Kaiser Family Foundation
**Vaccine mandates (state employees)**	History of COVID vaccine mandate for state employees (Yes/No/Partial)	COVID policy	None	1/1/20 - 2/15/22	log(cumulative infections per capita)	leave-one-out mandate PCA	Kaiser Family Foundation
**Mandate propensity**	First principal component of a PCA of the following variables: mask mandates, stay-at-home orders, restaurant closures, bar closures, gathering restrictions, primary school closures, higher education closures, and gym/pool/leisure closures.	COVID policy	None	4/1/20 - 6/1/21	mean(log(daily infections per capita))	population density + comorbidities	*Constructed*

**Mask use**	Average proportion of the population who always wear a mask when leaving home	COVID behavior	None	4/1/20 - 2/15/22	mean(log(daily infections per capita))	NA	IHME COVID-19 database
**Mobility**	Average daily relative change in mobility from pre-pandemic baseline	COVID behavior	None	4/1/20 - 2/15/22	mean(log(daily infections per capita))	NA	Google Global COVID-19 Community Mobility Report; Apple Inc. COVID-19 Mobility Trends Report; United States Mobility Changes in Response to COVID-19 2020 - Descarte Labs; United States SafeGraph Social Distancing Metrics 2020.
**Vaccine uptake**	Fully vaccinated person-days per total person-days, where fully vaccinated is defined as receiving 2 doses of a two-dose regimen or a single dose of a one-dose regimen	COVID behavior	None	4/1/20 - 2/15/22	mean(log(daily infections per capita))	NA	IHME COVID-19 database
**Infections**	Cumulative infections per capita	COVID outcomes	Log, Normalize	4/1/20 - 2/15/22	mean(log(daily infections per capita))	NA	IHME COVID-19 database
**Deaths**	Cumulative deaths per capita	COVID outcomes	Log, Normalize	4/1/20 - 2/15/22	mean(log(daily infections per capita))	NA	IHME COVID-19 database
**IFR**	Age-standardised Infection-fatality rate	COVID outcomes	Normalize	4/1/20 - 2/15/22	mean(log(daily infections per capita))	NA	IHME COVID-19 database

**Hospitalisations**	Cumulative hospitalisations per capita	COVID outcomes	Normalize	4/1/20 - 2/15/22	mean(log(daily infections per capita))	NA	IHME COVID-19 database

3.6 Dependent variables by state

Standardised cumulative infection and death rates, relative reduction in cumulative GDP and employment, and change in fourth-grade math and reading test scores for 50 US states and Washington DC

State	Standardized cumulative infection rate, per 10,000 (Jan 1, 2020 – Dec 15, 2021)	Standardized cumulative death rate, per 100,000 (Jan 1, 2020 – July 31, 2022)	Cumulative GDP relative to expected cumulative GDP (Jan 1, 2020 – July 31, 2022)	Cumulative employment relative to expected cumulative employment (Jan 1, 2020 – July 31, 2022)	Change in 4th grade reading scores (Fall 2019 to fall 2022)	Change in 4th grade math scores (Fall 2019 to fall 2022)
Alabama	5554	429	0.99	0.97	1.57	0.39
Alaska	4668	443	0.94	0.94	-0.54	-6.39
Arizona	4342	581	0.99	0.99	-0.36	-5.63
Arkansas	4918	400	1.01	0.99	-3.10	-5.11
California	3179	418	0.95	0.95	-2.09	-4.36
Colorado	4841	473	0.95	0.97	-2.01	-5.70
Connecticut	3662	293	0.96	0.95	-5.20	-6.90
Delaware	3979	311	0.93	0.96	-9.39	-13.74
District of Columbia	3869	526	0.97	0.91	-7.53	-11.99
Florida	5757	313	1.00	0.98	0.03	-5.13
Georgia	5453	447	0.96	0.98	-2.10	-2.74
Hawaii	1525	147	0.91	0.89	1.29	-2.07
Idaho	6246	469	1.00	1.02	-7.62	-5.70
Illinois	5748	342	0.98	0.95	-0.39	-0.32
Indiana	4278	332	0.98	0.97	-4.45	-5.73
Iowa	5939	344	0.95	0.96	-2.48	-0.86
Kansas	4616	371	0.98	0.96	-4.45	-4.41

Kentucky	5685	341	0.96	0.97	-4.33	-5.51
Louisiana	4785	385	0.93	0.94	2.47	-2.73
Maine	2914	218	1.00	0.96	-8.43	-7.69
Maryland	4224	285	0.96	0.95	-7.36	-9.97
Massachusetts	3707	355	0.98	0.94	-4.29	-5.59
Michigan	5395	326	1.00	0.94	-6.43	-3.95
Minnesota	4826	342	0.96	0.94	-6.92	-9.40
Mississippi	5425	488	0.99	0.98	-2.18	-7.15
Missouri	5227	342	0.97	0.97	-4.64	-6.03
Montana	5215	420	1.02	1.00	-2.95	-2.59
Nebraska	5883	298	0.97	0.97	-3.75	-2.21
Nevada	5082	453	0.93	0.96	-6.09	-6.28
New Hampshire	3453	215	1.05	0.96	-1.72	-5.17
New Jersey	4893	370	0.96	0.95	-4.46	-6.70
New Mexico	5014	521	0.91	0.95	-5.48	-9.86
New York	4916	325	0.96	0.92	-6.03	-9.64
North Carolina	5070	348	0.99	0.99	-5.15	-5.45
North Dakota	4626	328	0.94	0.95	-3.65	-2.85
Ohio	6472	293	0.97	0.95	-3.41	-3.38
Oklahoma	5041	412	0.92	0.96	-8.24	-7.71
Oregon	2878	330	0.97	0.95	-7.41	-8.16
Pennsylvania	5057	297	0.95	0.94	-4.32	-6.40
Rhode Island	3666	321	1.00	0.95	-2.93	-5.21
South Carolina	5060	415	0.96	0.97	0.41	-2.53

South Dakota	5542	354	1.04	0.99	-3.93	-2.29
Tennessee	5634	421	1.01	0.99	-5.06	-3.35
Texas	5718	429	0.94	0.98	-1.84	-5.04
Utah	5385	467	1.00	1.01	-4.06	-4.18
Vermont	1723	249	1.00	0.93	-4.85	-4.61
Virginia	2964	336	0.98	0.96	-9.60	-10.53
Washington	3753	286	0.99	0.96	-3.00	-4.58
West Virginia	4003	322	0.96	0.96	-7.87	-5.53
Wisconsin	5911	341	0.97	0.96	-2.33	-1.41
Wyoming	6144	422	0.91	0.97	-1.90	-2.69

A summary table of key outcomes. Cumulative infection rates (January 1, 2020 – December 15, 2021) were standardised for population density. Cumulative death rates (January 1, 2020 – July 31, 2022) were standardised for age and presence of comorbidities. Cumulative GDP and employment (January 1, 2020 – July 31, 2022) were measured relative to their expected values, based on pre-COVID-19 trends and were standardised to ensure each economic sector (i.e., tourism, agriculture, etc.) were equally represented in each state. Fourth-grade math and science test scores were measured in the fall of 2019 and fall of 2022, with the change over time reported.

3.7 Correlation among independent variables

Pearson correlation coefficients for key independent and control variables

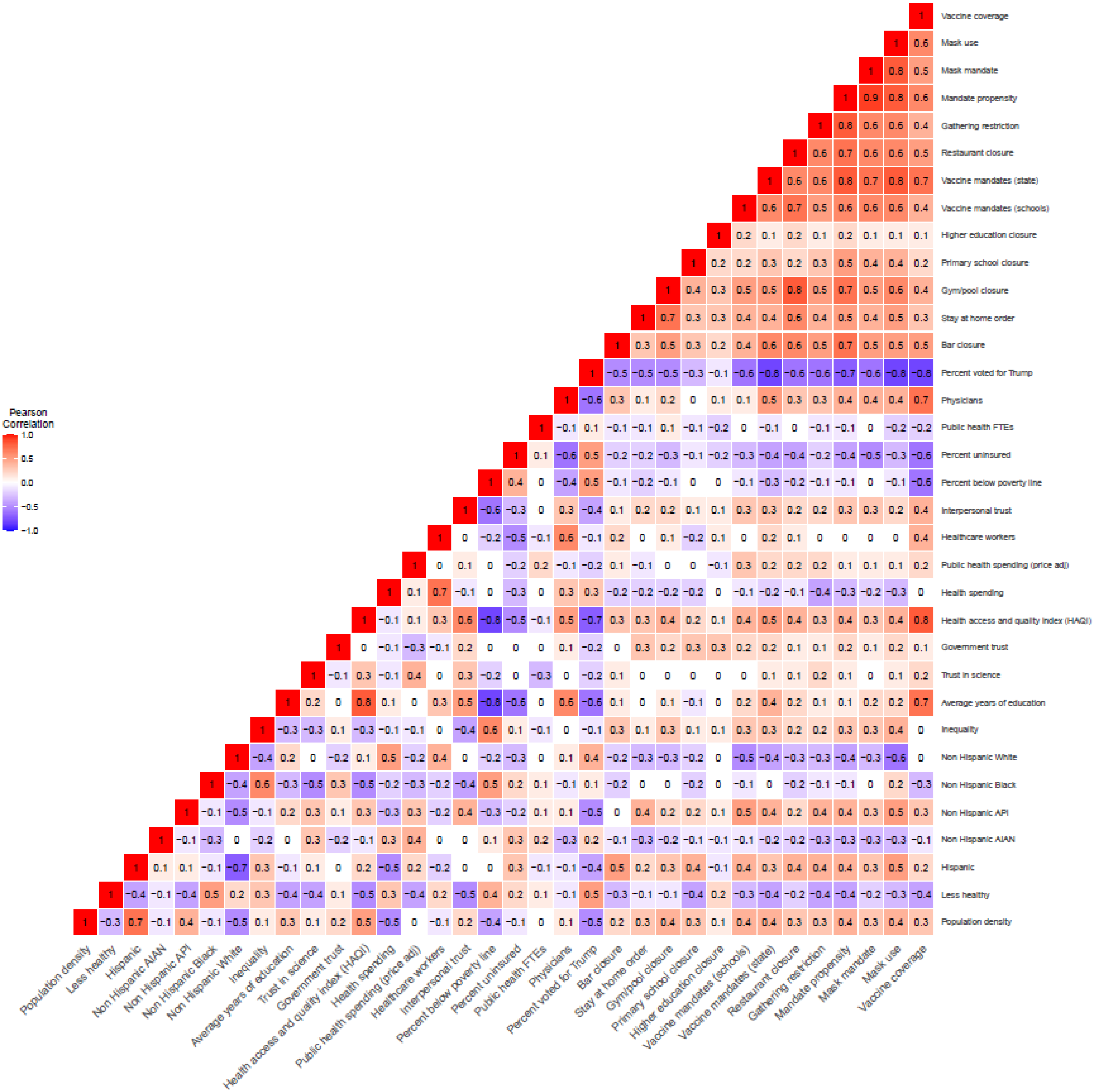

3.8 Reduction in economic indicators and education scores by state

Reductions in standardised GDP, standardised employment, and fourth-grade math and reading scores by state, January 1, 2020 to July 31, 2022

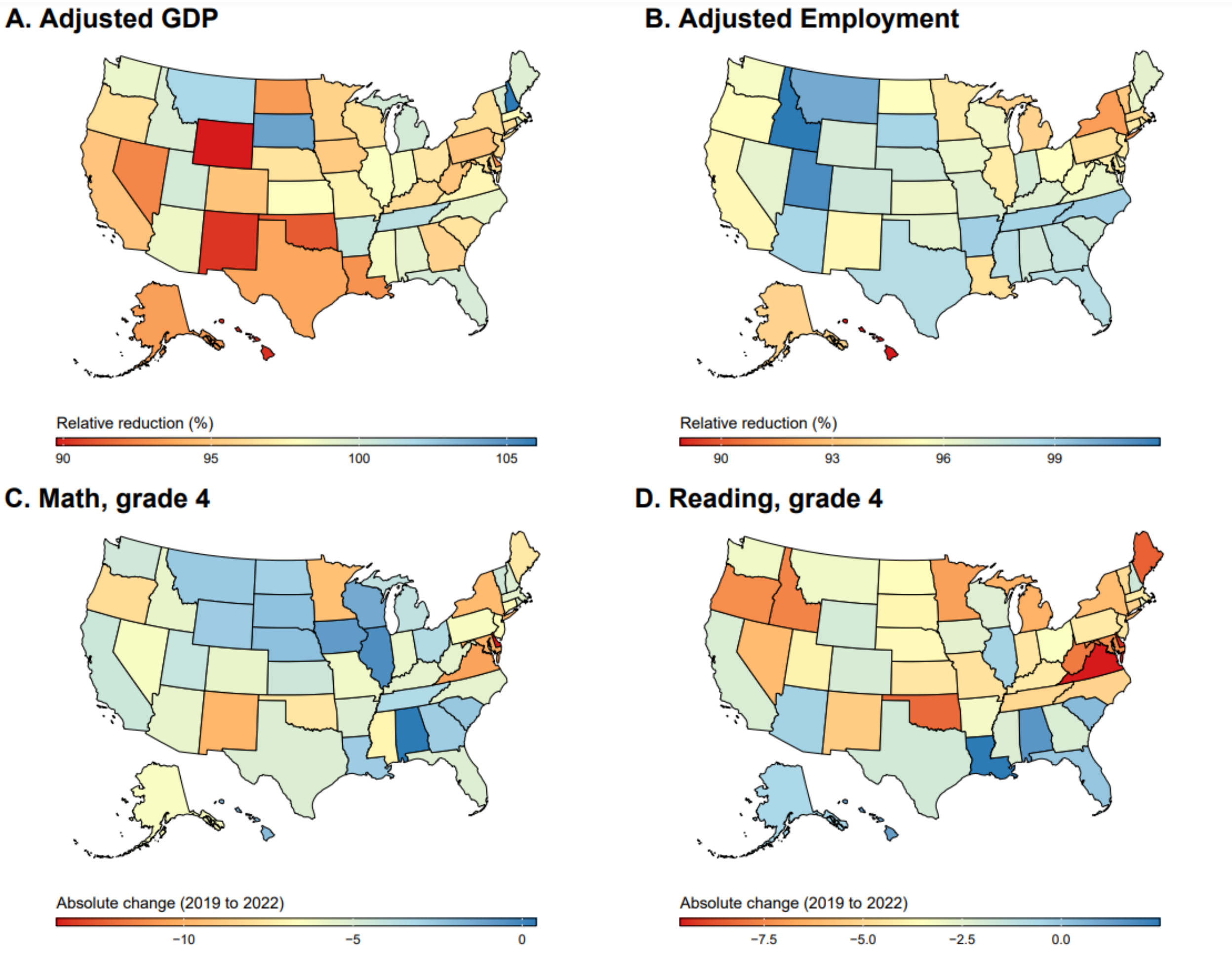

Each panel shows the reduction in economic indicators or education scores during the pandemic. Panel A shows the relative reduction of state GDP after the GDP has been standardised to address states' reliance on different economic sectors, such as tourism and agriculture. The reported relative reduction is the value of cumulative reduction in economic production (GDP) relative to the expected economic production based on projections from changes in standardised GDP in 2019. Panel B shows the relative reduction of state employment count after the employment rate has been standardised to address states' reliance on different economic sectors. The reported relative reduction is the value of cumulative reduction in person-days employed relative to the expected person-days employed had the employment rates stayed constant at the 2019 rate during the pandemic. Panel C shows the absolute reduction in the mean fourth-grade math score between fall of 2019 and fall of 2022. Panel D shows the absolute reduction in the mean fourth-grade reading score between fall of 2019 and fall of 2022. This figure describes changes and does not identify key association between these variables.

3.9 Syndemic framework for COVID-19

Syndemic framework for COVID-19: interaction of race, politics, poverty, education, trust, and access to health-care

Panel A. Low performers

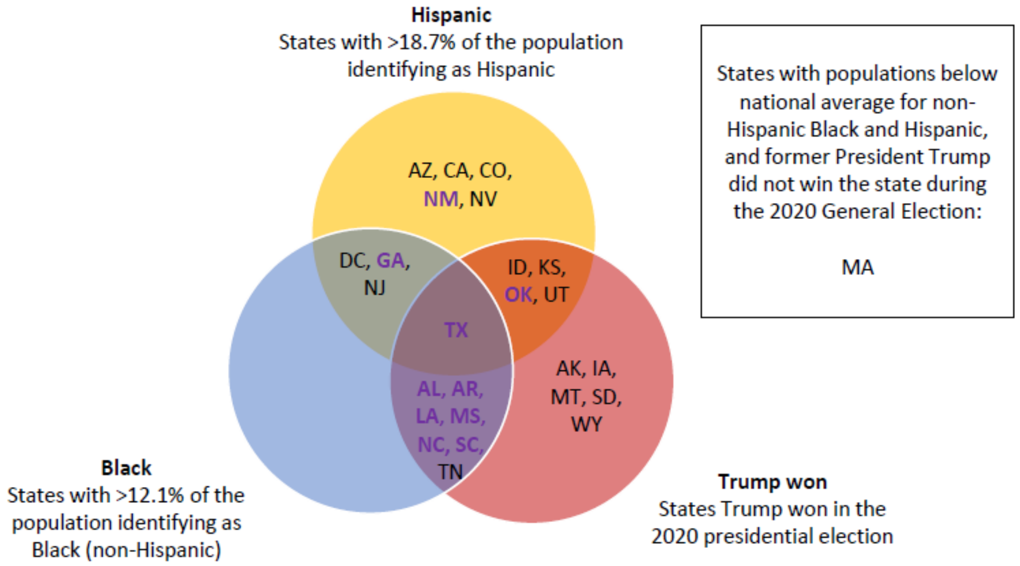

Panel B. High performers

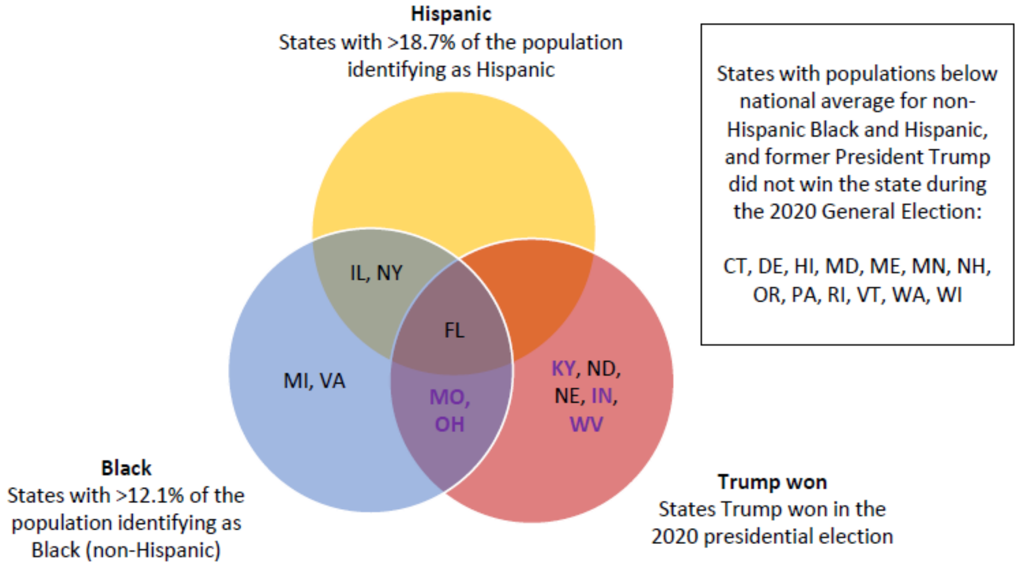

Panel A shows the 25 states, plus the District of Columbia, with the highest standardized cumulative COVID-19 death rate, categorized according to the following criteria: (1) states with populations exceeding the national value (18.7%) for percent of individuals identifying as Hispanic (2) states with populations exceeding the national value (12.1%) for percent of individuals identifying as Black (non-Hispanic) and (3) states where former President Trump won during the 2020 general election. Data on

race and ethnicity were sourced from the 2020 US Census. States that are shown in bold purple text exhibit high poverty rates, low education, low levels of interpersonal trust, and low access to quality healthcare, defined as being in the bottom 50th percentile of states across all four metrics. Panel B shows the same information for the 25 states with the lowest standardized cumulative COVID-19 death rate.

3.10 Mandate propensity

Mandate propensity is a summary measure describing a state's usage of physical distancing and mask mandates. It is calculated as the first component of a PCA of the following mandates: bar closures, restaurant closures, gathering restrictions, primary school closures, higher education closures, gym closures, mask mandates, and stay at home orders. Each mandate was expressed as the proportion of days that the mandate was in effect during the analysis period.

Mandate propensity score by state

State	Mandate Propensity
Alabama	0.33
Alaska	0.16
Arizona	0.70
Arkansas	0.39
California	0.95
Colorado	0.65
Connecticut	0.73
Delaware	0.55
District of Columbia	1.04
Florida	0.22
Georgia	0.29
Hawaii	0.83
Idaho	0.27
Illinois	0.81
Indiana	0.41
Iowa	0.24
Kansas	0.24
Kentucky	0.54
Louisiana	0.48
Maine	0.71
Maryland	0.48
Massachusetts	0.75
Michigan	0.73
Minnesota	0.61
Mississippi	0.39
Missouri	0.15

State	Mandate Propensity
Montana	0.28
Nebraska	0.20
Nevada	0.75
New Hampshire	0.41
New Jersey	0.52
New Mexico	0.86
New York	0.63
North Carolina	0.67
North Dakota	0.11
Ohio	0.51
Oklahoma	0.08
Oregon	0.83
Pennsylvania	0.57
Rhode Island	0.62
South Carolina	0.29
South Dakota	0.10
Tennessee	0.25
Texas	0.47
Utah	0.35
Vermont	0.50
Virginia	0.52
Washington	1.01
West Virginia	0.51
Wisconsin	0.28
Wyoming	0.38

Section 4: Methods

4.1 Modeling COVID-19 Deaths

Daily estimates of COVID-19 deaths by state from January 1, 2020 through July 31, 2022 were retrieved from IHME's COVID-19 database. These estimates were based on death reporting data from Johns Hopkins University and have been adjusted to correct for missingness and reporting lags. Mortality rates were then adjusted to account for under-reporting by applying a scaling factor based on estimated all-cause excess mortality, reported COVID-19 deaths, and estimated COVID-19 mortality assuming the maximum observed infection-detection ratio (IDR). The resulting estimates reflect all COVID-19 mortality, both reported and unreported.

To account for differences in age patterns across states, we indirectly age-standardized the death rate to the national age pattern. Estimated age-specific death proportions were based on age-specific mortality data as published by the National Center for Health Statistics (NCHS). These proportions were applied to the IHME's all-age mortality estimates to generate estimates of the age-specific death counts required to age-standardize.

We fit a series of log-linear models, regressing the logged age-standardized cumulative mortality rate during the period January 1, 2020 to July 31, 2022 on state-level factors of interest. These factors comprised 3 broad categories: pre-pandemic state characteristics, policies adopted by states during the pandemic, and population-level behavioral responses to the pandemic. Section 3.1 of this appendix describes all factors investigated in the mortality analysis, their data source, and important features of the model.

All mortality models controlled for age and the prevalence of comorbid conditions. Age was accounted for by age-standardizing the mortality rate, as described above. To adjust for comorbidities, we performed a principal component analysis (PCA) to create a summary metric of seven health conditions and risk behaviors that increase the risk of COVID-19. These conditions were age-standardized prevalence of asthma, cancer, chronic obstructive pulmonary disease (COPD), cardiovascular disease, and diabetes, along with body-mass index (BMI) and smoking rate. We included the first principal component of this PCA as a covariate in all mortality models.

Additionally, all models where the factor of interest was a COVID-19 mandate (such as school closures or stay at home orders) included a covariate to control for all other COVID-19 mandates. We used PCA to create a summary metric of all mandates besides the factor of interest and included the first principal component of this "leave-one-out" mandate PCA as a covariate in the regression.

For factors without daily data (all pre-COVID-19 policies, plus vaccine mandates), we defined the dependent variable as the logged cumulative infection rate. For factors with daily data, we defined the dependent variable as the mean of the logged daily infection rate. We specified a start date of April 1, 2020 for these models to avoid undefined values when taking the log of a 0 infection rate. The advantages to using the mean of the logged daily mortality rate instead of the logged cumulative mortality rate are described in Section 4.1.1 of this appendix.

To capture uncertainty associated with model inputs, we fit 100 independent models for each factor of interest, using the 100 estimates of mortality produced by IHME for each location-day. To capture parameter uncertainty, we generated 100 estimates of the beta coefficient by sampling from a multivariate normal distribution where the mean was defined by the point estimates for fitted betas and

the variance was defined by the variance-covariance matrix. Since each of the 100 draws of mortality generated 100 draws of the beta coefficients, we generated 10,000 estimates per factor to describe the relationship with mortality. We summarized these draws by reporting the mean value along with the 95% uncertainty interval (UI), which is constructed from the 2.5th and 97.5th percentile of the 10,000 estimates.

4.1.1 Dependent variable specification

Because of policy endogeneity, regressing the log of the cumulative death rate on daily mandate data results in biased estimates of the association between mandates and deaths. Instead, specifying the dependent variable as the mean of the logged daily death rate minimizes bias and reduces the risk of overestimating the mandate effect on mortality. COVID-19 behaviors (mask use, mobility, vaccine coverage) are also vulnerable to this endogeneity problem. Therefore, we used the mean of the logged daily rate as the dependent variable specification when examining the relationship between deaths and mandates, and between deaths and behaviors. For the same reasons, we used this approach for infections as well.

**Notation**	**Definition**
$f(i,t)$	Counterfactual infections at time t for location i
$\tilde{f}(i,t)$	Observed infections at time t for location i
$m(i,t)$	Fraction of mandates that are active at time t for location i
α	Effect of mandate intensity on infections
T	Number of days in dataset
y_i	Average of log infections across time in location i
m_i	Average of mandate intensity across time in location i

Model derivation: Using the above notation, we consider the following model that relates mandates and counterfactual infections (absence of mandates) to observed infections:

$$\tilde{f}(i,t) = \exp(\log(\alpha) m(i,t)) f(i,t)$$

When $m(i,t) = 0$, the counterfactual infections match the observed, while for $m(i,t) = 1$, we have

$$\tilde{f}(i,t) = \alpha f(i,t)$$

Taking the logarithm of the observation model, we obtain

$$\log(\tilde{f}(i,t)) = \alpha\, m(i,t) + \log(f(i,t))$$

Adding across the time interval and dividing by total time, we obtain the model

$$y_i = \alpha m_i + \frac{1}{T}\sum_t \log\left(f(i,t)\right)$$

where y_i is the average of log observed infections in location i, m_i is the average mandate intensity in location i, while the term $\frac{1}{T}\sum_t \log(f(i,t))$ is unobserved.

Regression approach: To approximate the ideal model, we consider the regression

$$y_i = \beta_0 + \beta_1 m_i + \sum_j \beta_j Cov_j$$

Here, β_0 captures the intercept, β_1 reflects the action of the average mandate with

$$\beta_1 \approx \log \alpha$$

and additional covariates Cov_j capture important features that are different between locations.

4.2 Modeling COVID-19 Infections

Daily estimates of COVID-19 infections by state from January 1, 2020 through July 31, 2022 were retrieved from IHME's COVID-19 database. These estimates were based on case reporting data from JHU. Estimated infection counts were intended to reflect all infections, including asymptomatic infections and infections not confirmed by a test. To capture uncertainty, IHME generated 100 estimates of infections for each location-day. This underlying data uncertainty was propagated throughout the analysis as described in this section.

We fit a series of log-linear models, regressing the logged cumulative infection rate during the period January 1, 2020 to December 15, 2021 on state-level factors of interest. As with the mortality regression analysis, the infection analysis investigated factors falling into three broad categories: pre-pandemic state characteristics, COVID-19 policies, and COVID-19 behaviors. Section 3.2 of this appendix describes all factors investigated in the infections analysis, their data source, and important features of the model.

For analysis of COVID-19 infections, we selected an end date of December 2021 to deliberately exclude the Omicron wave. Omicron's transmissibility and immune escape produced an explosion of infections across the entire United States, making it more difficult to discern which factors were associated with diminished infection rates. As a sensitivity analysis, we conducted our analysis of infections for alternative time periods (Supplementary Appendix: Section 5), including the Omicron period (defined as December 15, 2021 to July 31, 2022).

All infection models controlled for population density because it has a direct impact on transmission yet cannot readily be addressed by policy action. All models where the factor of interest was a COVID-19 mandate included an additional covariate to control for all other COVID-19 mandates. We used PCA to create a summary metric of all mandates besides the factor of interest and included the first principal component of this "leave-one-out" mandate PCA as a covariate in the regression.

For factors without daily data (all pre-COVID-19 policies, plus vaccine mandates), we defined the dependent variable as the logged cumulative infection rate. For factors with daily data, we defined the

dependent variable as the mean of the logged daily infection rate. We made this decision for the same reasons as described in Section 5.1.1. For models regressing the mean of the logged daily infection rate, we specified a start date of April 1, 2020 to avoid undefined values when taking the log of a 0 infection rate.

To capture uncertainty associated with model inputs, we fit 100 independent models for each factor of interest, using the 100 estimates of infections produced by IHME for each location-day. To capture parameter uncertainty, we generated 100 estimates of the beta coefficient by sampling from a multivariate normal distribution where the mean was defined by the point estimates for fitted betas and the variance was defined by the variance-covariance matrix. Since each of the 100 draws of infections generated 100 draws of the beta coefficients, the resulting analysis generated 10,000 estimates for each factor of interest. We summarized these draws by reporting the mean value as well as the 95% uncertainty interval (UI), which is based on the 2.5th and 97.5th percentile of the 10,000 estimates.

4.3 Modeling GDP

Quarterly Real GDP data by state and industry from 2018-2022 was downloaded from the U.S. Bureau of Economic Analysis (BEA) expressed in millions of chained 2012 dollars. In order to industry-standardise state GDP to compare across state, we generated industry weights using the Q4 2019 values. The table below lists all 23 industries included and the associated weight. As a robustness check we compared these weights to the average across each quarter of 2019 and found the values to be highly correlated.

The GDP data was very complete with less than 5% missingness due to disclosure suppressions or confidential information. However, suppressed estimates were included in higher level totals. We handled missing data through a series of imputation methods. For example, if an estimate was missing for a particular quarter, we forward filled state fractions using historical values. If estimates were missing across all quarters, we used the mean fraction from adjacent states. Once industry standardized, GDP was transformed into per capita space using 2019 population estimates from the Global Burden of Disease Study (CITE).

Our dependent variable for GDP is expressed as observed GDP relative to expected GDP over the time period of interest. In order to generate our counterfactual of expected GDP in absence of COVID-19 we used the average slope between 2018 – 2019 to forecast GDP through 2022. GDP was treated as a stepwise function over time meaning for each day within a quarter, the value was constant. Ultimately, the final variable was constructed by taking the ratio of cumulative observed GDP to cumulative expected GDP. A value less than 100 represents a state that did not meet their expected GDP in absence of COVID-19 while a value above 100 represents a state that had their cumulative GDP surpass the expected GDP during the timer period of interest.

Industry	National Fraction
Agriculture, forestry, fishing and hunting	0.0115
Mining, quarrying, and oil and gas extraction	0.0263
Utilities	0.0154
Construction	0.0352
Durable goods manufacturing	0.0645

Nondurable goods manufacturing	0.0531
Wholesale trade	0.0578
Retail trade	0.0589
Transportation and warehousing	0.0293
Information	0.0665
Finance and insurance	0.0669
Real estate and rental and leasing	0.1227
Professional, scientific, and technical services	0.0811
Management of companies and enterprises	0.0229
Administrative and support and waste management and remediation services	0.0304
Educational services	0.0119
Health care and social assistance	0.0762
Arts, entertainment, and recreation	0.0107
Accommodation and food services	0.0277
Other services (except government and government enterprises)	0.0194
Federal civilian	0.0221
Military	0.0096
State and local	0.0798

4.4 Modeling Employment

Because the covid pandemic had a differential impact on different sectors of the economy, we sector-standardised our employment measure. Sector weights were created using the U.S. employment for Q4 of 2019. As a robustness check, all 12 months of 2019 were used to create sector weights, which resulted in nearly identical weights. Monthly, seasonally adjusted employment time series data for each state-sector was obtained from FRED (fred.stlouisfed.org). The sectors used in our analysis were:

- Construction, Mining, and Logging
- Education and Health Services
- Financial Activities
- Government
- Information
- Leisure and Hospitality
- Manufacturing
- Professional and Business Services
- Other Services
- Trade, Transportation, and Utilities

The employment metric used as the dependent value was constructed as follows. First, we construct the ratio of sector-standardised employment to a counterfactual in which per-capita employment remained constant at the average Q4 2019 level. This ratio is summed across the time period and then normalized so that a value of 1 would indicate full employment (matching the counterfactual) across the entire time period.

4.5 Modeling Education

National Assessment of Educational Progress (NAEP) assessment scores were downloaded from the National Center for Education Statistics. State level scores were downloaded for 2019 and 2022 for the following grade and subject combinations:

- 4th grade math
- 8th grade math
- 4th grade reading
- 8th grade reading

The NAEP sampling process is designed to be representative of the entire student population in the USA; therefore we did not make additional adjustments to the reported results.

A model is estimated for each subject-grade. Our dependent variable for education is expressed as the change in score from 2019 to 2022. The NAEP was administered between January and March in 2022. Therefore, the midpoint of this range, February 15, 2022 was used as the end date for test score analyses.

Section 5: Sensitivity Analyses

5.1 Infection and death results using the logged cumulative rate as the dependent variable for all models

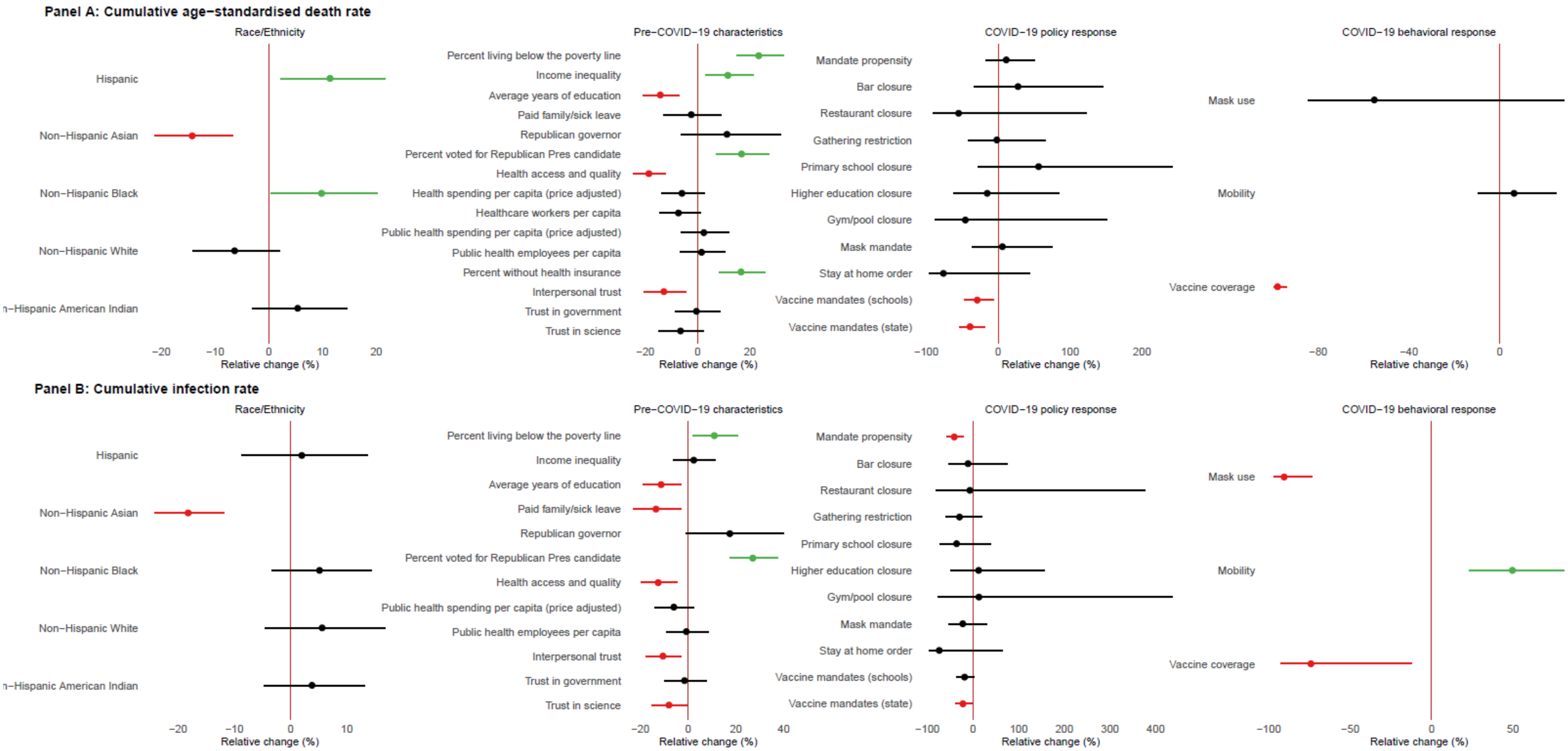

Panel A reports the relative reduction or increase in cumulative age-adjusted deaths per capita that was associated with race/ethnicity, pre-COVID-19 state characteristics, COVID-19 policy response, and COVID-19 behavioural response. The dependent variable is specified as the logged cumulative death rate. The cumulative death rate models control for comorbidity, constructed as the first principal component of asthma, cancer, COPD, cardiovascular disease, diabetes, BMI, and smoking prevalence. The models assessing COVID-19 policy response (other than mandate propensity) also include an additional control variable that was the first component of all the other policy responses. These estimated associations are not reported. The reported associations for the policy response should be interpreted as additional to the association tied to the mandate propensity variable. Panel B reports the relative reduction or increase in cumulative infections per capita that was associated with race/ethnicity, pre-COVID-19 state characteristics, COVID-19 policy response, and COVID-19 behavioural response. The cumulative infection rate models control for population density. The dependent variable is specified as the logged cumulative infection rate. Error bars show

95% confidence intervals that account for death data uncertainty, and markers that are green or red are statistically significant at the 95% level. In this sensitivity analysis, all dependent variables were specified as the logged cumulative rate. This is in contrast to the main analysis, for which models examining time-varying factors (such as policy and behavioral response variables) employed the mean of the log-transformed daily rate as the dependent variable.

5.2 Infection and death results using negative binomial model

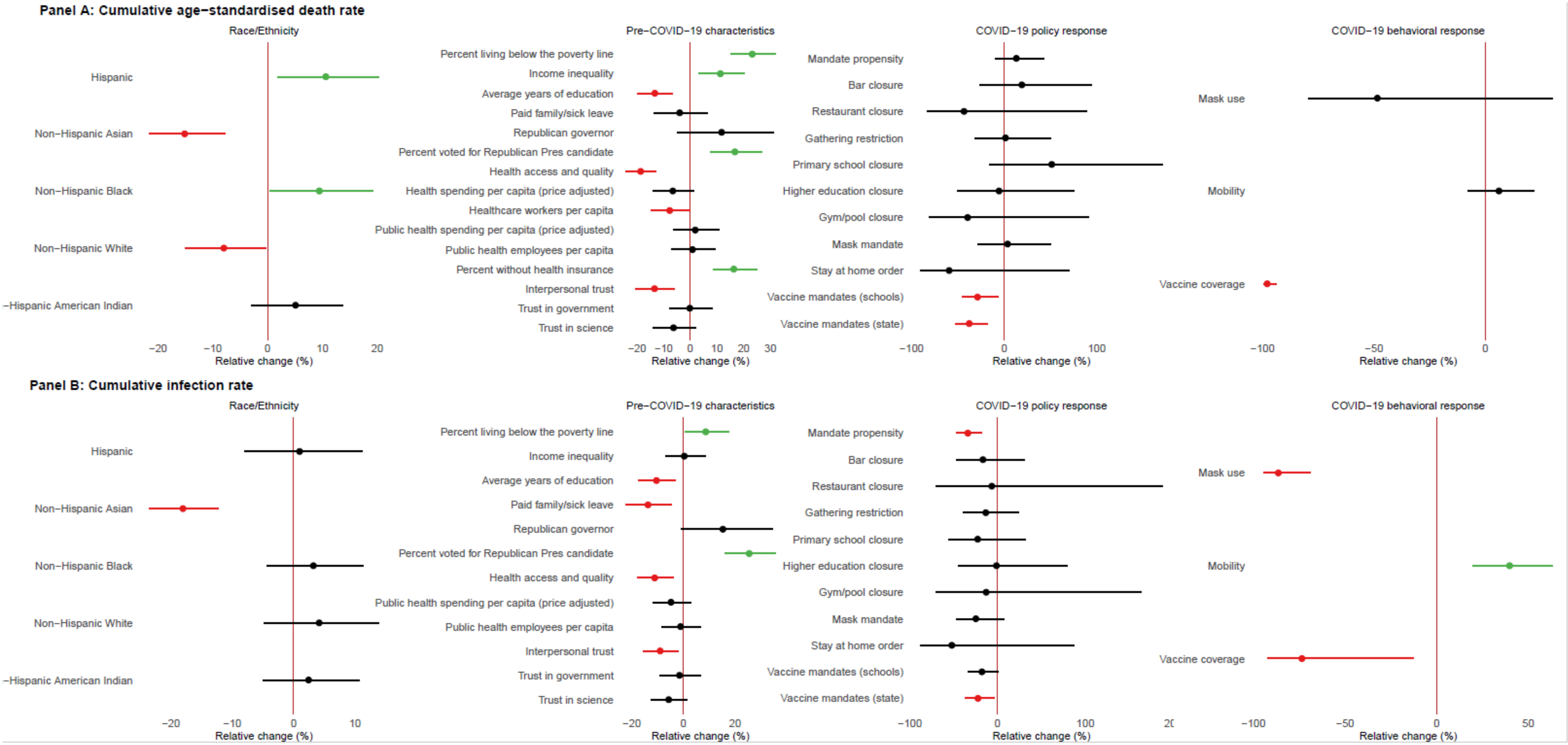

Panel A reports the relative reduction or increase in cumulative age-adjusted deaths per capita that was associated with race/ethnicity, pre-COVID-19 state characteristics, COVID-19 policy response, and COVID-19 behavioural response. The cumulative death rate models control for comorbidity, constructed as the first principal component of asthma, cancer, COPD, cardiovascular disease, diabetes, BMI, and smoking prevalence. The models assessing COVID-19 policy response (other than mandate propensity) also include an additional control variable that was the first component of all the other policy responses. These estimated associations are not reported. The reported associations for the policy response should be interpreted as additional to the association tied to the mandate propensity variable. Panel B reports the relative reduction or increase in cumulative infections per capita that was associated with race/ethnicity, pre-COVID-19 state characteristics, COVID-19 policy response, and COVID-19 behavioural response. The cumulative infection rate models control for population density. Error bars show 95% confidence intervals that account for death data uncertainty, and markers that are green or red are statistically significant at the 95% level. All models in this sensitivity analysis were specified as a negative binomial generalized linear model.

5.3 Infection and death results including poverty as a control for COVID-19 policies and behaviors

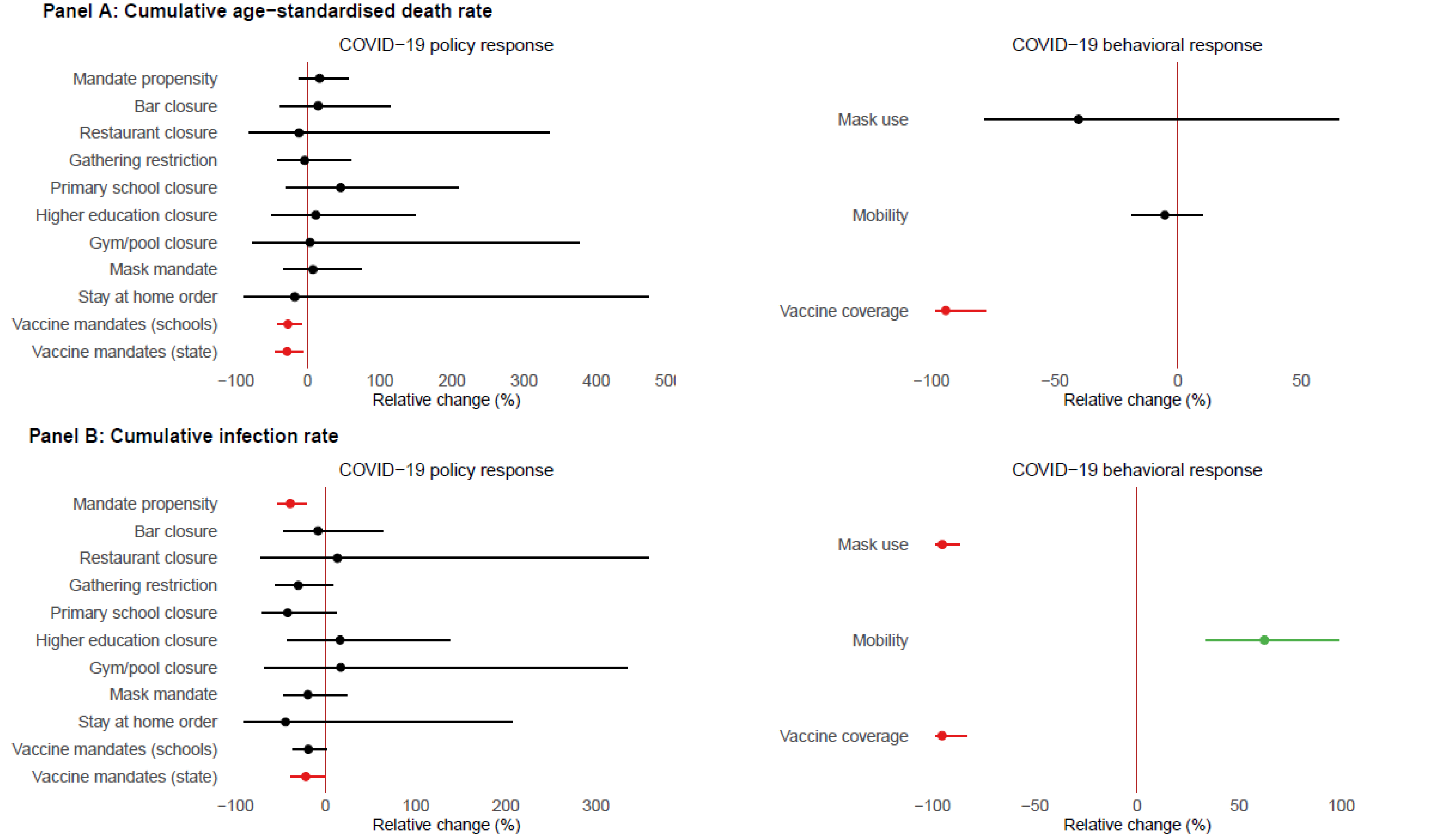

Panel A reports the relative reduction or increase in cumulative age-adjusted deaths per capita that was associated with COVID-19 policy and behavioural response. The cumulative death rate models control for poverty and comorbidity, constructed as the first principal component of asthma, cancer, COPD, cardiovascular disease, diabetes, BMI, and smoking prevalence. Panel B reports the relative reduction or increase in cumulative infections per capita that was associated with COVID-19 policy and behavioural response. The cumulative infection rate models control for poverty and population density. The models assessing COVID-19 policy response (other than mandate propensity) also include an additional control variable that was the first component of all the other

policy responses. These estimated associations are not reported. The reported associations for the policy response should be interpreted as additional to the association tied to the mandate propensity variable. Error bars show 95% confidence intervals that account for death data uncertainty, and markers that are green or red are statistically significant at the 95% level.

5.4 Infection and death results including the first principal component of a PCA of all statistically significant pre-COVID-19 factors as control variables for COVID-19 policies and behaviours

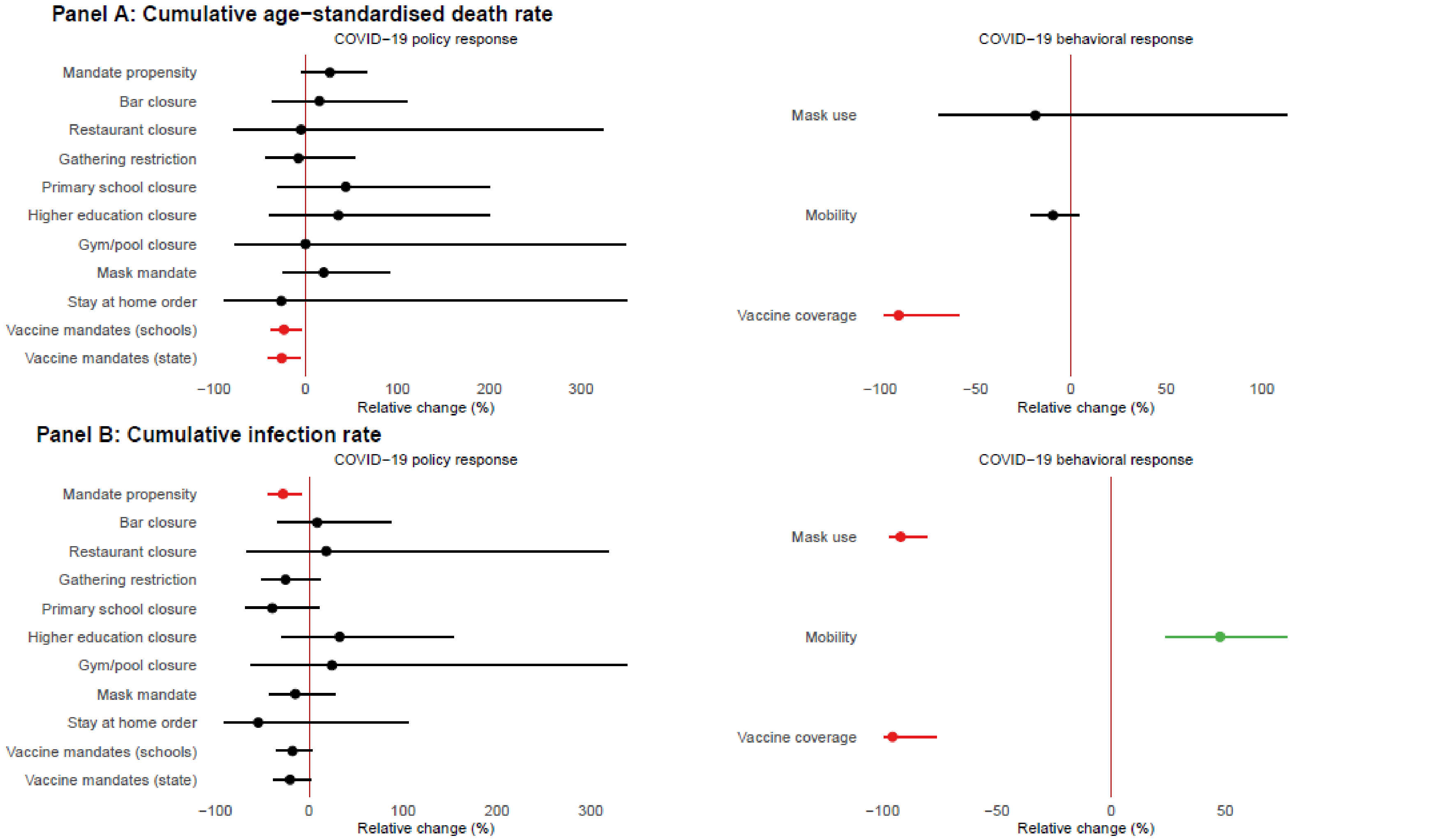

Panel A reports the relative reduction or increase in cumulative age-adjusted deaths per capita that was associated with COVID-19 policy and behavioural response. The cumulative death rate models control for comorbidity and statistically significant pre-COVID factors from the main analysis, constructed as the first principal component of poverty, inequality, education attainment, health access and quality, percent of the population without health insurance, and interpersonal trust. Panel B reports the relative reduction or increase in cumulative infections per capita that was associated with COVID-19 policy and behavioural response. The cumulative infection rate models control for population density and statistically significant pre-COVID factors from the main analysis, constructed as the first principal component of poverty, education attainment, existence of state-funded paid medical or family leave, health access and quality,

interpersonal trust, and trust in science. The models assessing COVID-19 policy response (other than mandate propensity) also include an additional control variable that was the first component of all the other policy responses. Error bars show 95% confidence intervals that account for death data uncertainty, and markers that are green or red are statistically significant at the 95% level.

5.5 Death results without control variables

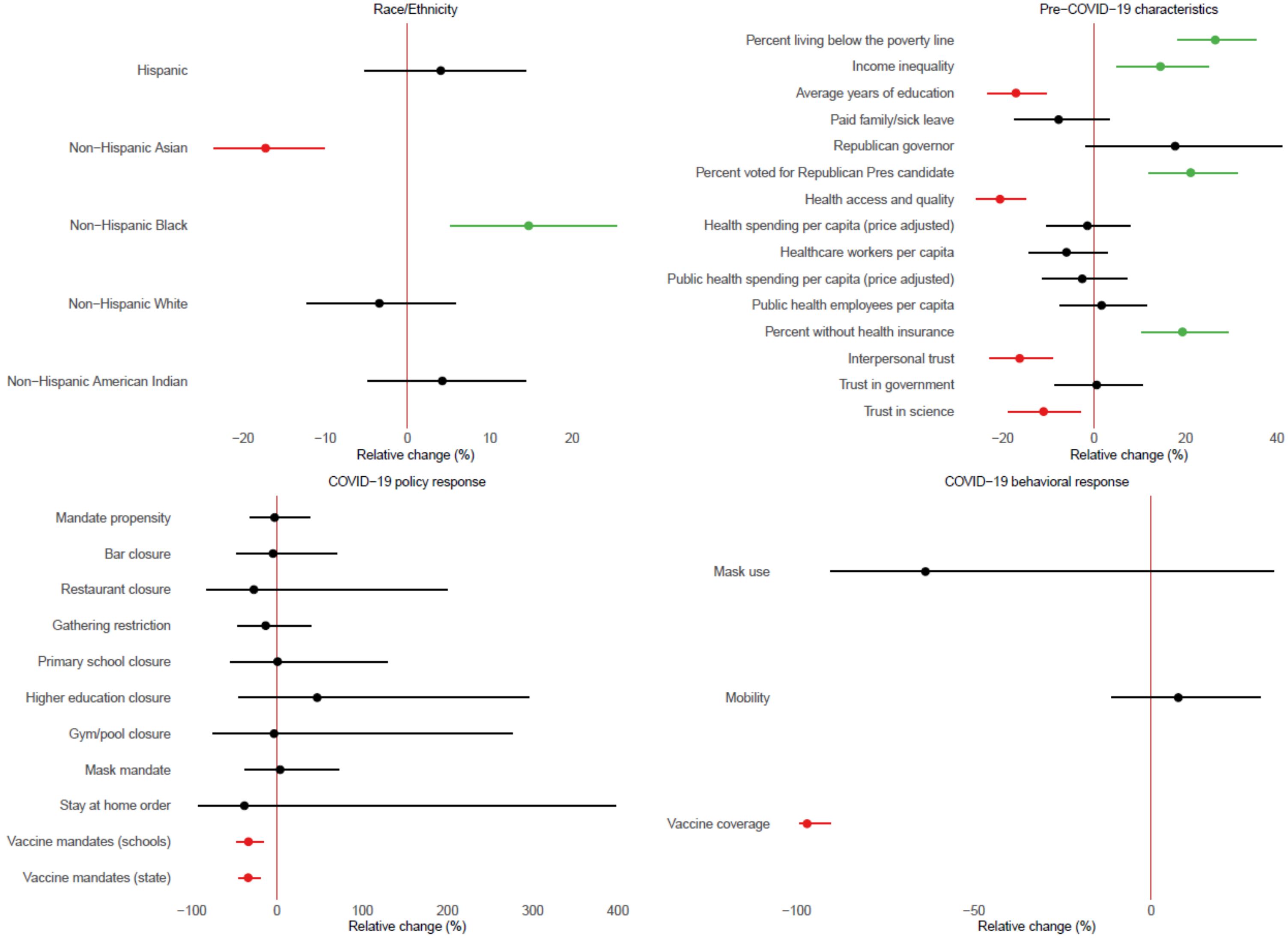

Figure 5.5 reports the relative reduction or increase in cumulative age-adjusted deaths per capita that was associated with race/ethnicity, pre-COVID-19 state characteristics, COVID-19 policy response, and COVID-19 behavioural response. In this sensitivity analysis, we did not adjust for underlying comorbidities. The models assessing COVID-19 policy response (other than mandate propensity) do include an additional covariate representing the first principal component of a PCA of all other policy responses. Error bars show 95% confidence intervals that account for death data uncertainty, and markers that are green or red are statistically significant at the 95% level.

5.6 Death results with no control variables and no age standardization of cumulative death rate

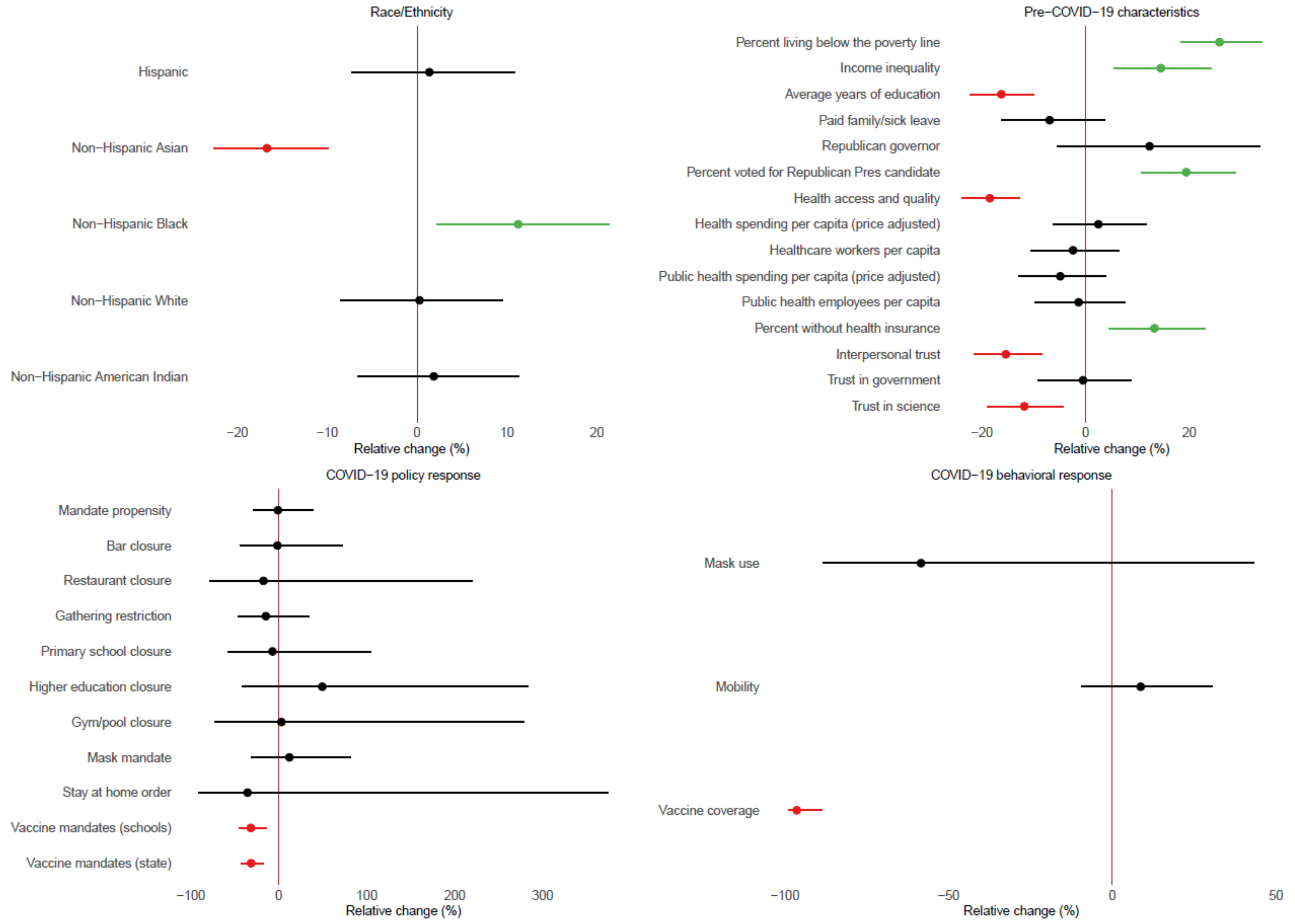

Figure 5.6 reports the relative reduction or increase in cumulative deaths per capita that was associated with race/ethnicity, pre-COVID-19 state characteristics, COVID-19 policy response, and COVID-19 behavioural response. In this sensitivity analysis, we did not age-adjust the cumulative death rate and did not adjust for underlying comorbidities or any other control variables. The models assessing COVID-19 policy response (other than mandate propensity) do include a covariate representing the first principal component of a PCA of all other policy responses. Error bars show 95% confidence intervals that account for death data uncertainty, and markers that are green or red are statistically significant at the 95% level.

5.7 Death results without control variables, dependent variable specified as cumulative reported deaths per capita

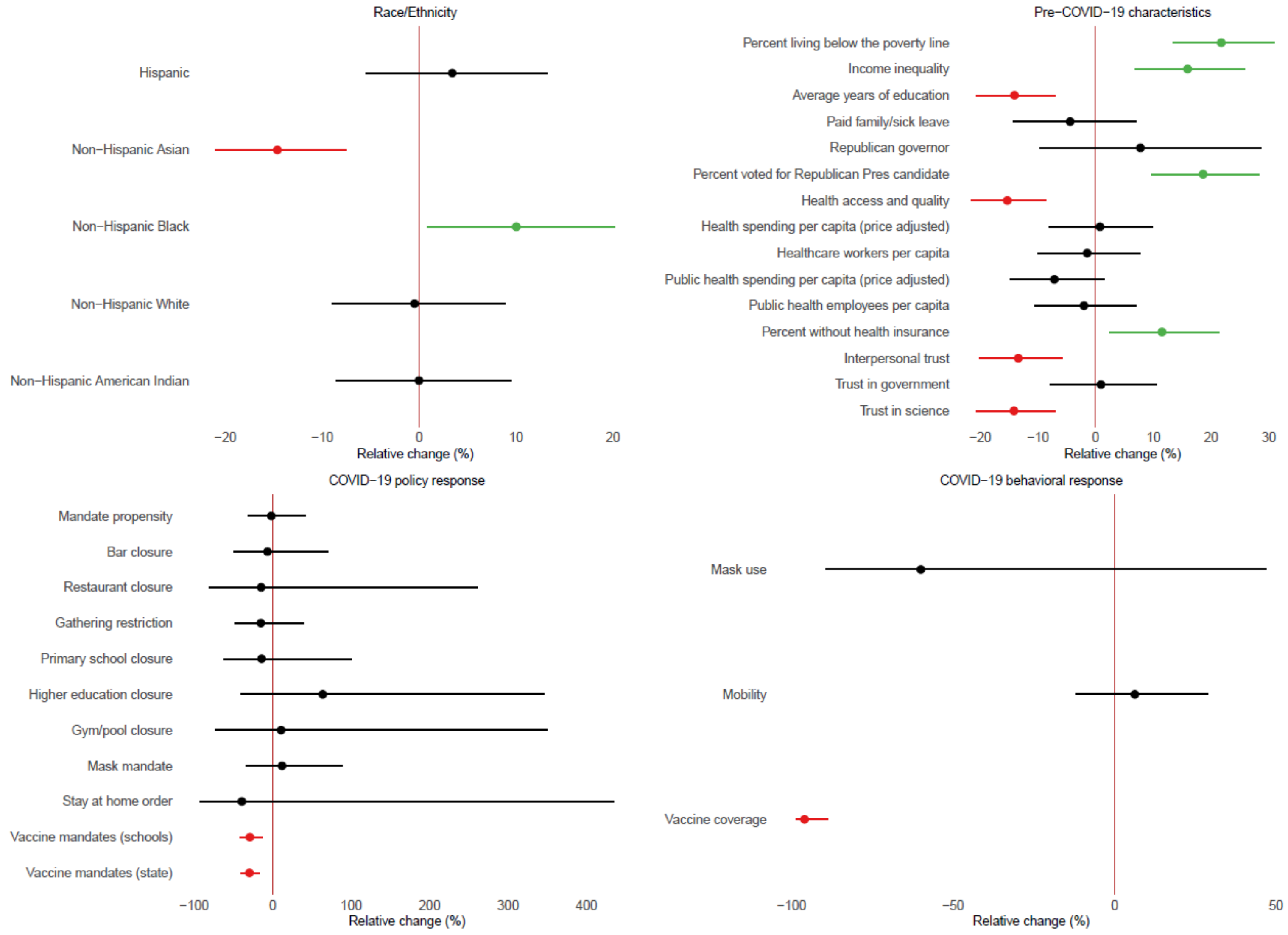

Figure 5.7 reports the relative reduction or increase in cumulative deaths per capita that was associated with race/ethnicity, pre-COVID-19 state characteristics, COVID-19 policy response, and COVID-19 behavioural response. In this sensitivity analysis, the dependent variable was specified as cumulative reported deaths per capita. Additionally, we did not adjust for underlying comorbidities or any other control variables. The models assessing COVID-19 policy response (other than mandate propensity) do include a covariate representing the first principal component of a PCA of all other policy responses. Error bars show 95% confidence intervals that account for death data uncertainty, and markers that are green or red are statistically significant at the 95% level.

5.8 Reporting E-Values for unmeasured confounding

We used the `EValue` package in R (VanderWeele TJ, Ding P (2011). "Sensitivity analysis in observational research: introducing the E-value." Annals of Internal Medicine, 167(4), 268–274.) to conduct a sensitivity analysis, calculating E-values for the assessment of unmeasured confounding. E-value is defined as the minimum association (on a risk ratio scale) that an unmeasured confounder would need to have with both the exposure and the outcome to fully explain away the observed treatment-outcome association, conditioned on measured covariates. A small E-value indicates that only a small amount of unmeasured confounding is needed to explain away the effect size estimate. Conversely, a large E-value indicates that a substantial amount of unmeasured confounding would be needed to explain away the effect size estimate.

E-Values for cumulative infection rate regressions

**Factor**	**E-value**
Non-Hispanic White	1.6
Non-Hispanic Black	1.6
Non-Hispanic Asian	3.1
Non-Hispanic American Indian	1.5
Hispanic	1.3
percent living below the poverty line	2.1
income inequality	1.4
health access and quality	2.4
public health employees per capita	1.2
public health spending per capita (price adjusted)	1.7
Republican governor	2.7
percent voted for Republican Pres candidate	3.6
interpersonal trust	2.2
trust in science	1.9
trust in government	1.3
paid family/sick leave	2.5
average years of education	2.2
citizen party affiliation	2.9
govt party affiliation	3.3
state house polarization	1.2
state senate polarization	1.2
affective polarization	1.8
economic connectedness	2.2
social clustering	1.6

volunteering rate	1.4
mask mandate	2.7
stay at home order	13.6
restaurant closure	2.2
bar closure	1.4
gathering restriction	2.8
primary school closure	3.1
higher education closure	2.7
gym/pool closure	1.8
vaccine mandates (schools)	3.6
vaccine mandates (state)	4.3
mandate propensity	3.9
mask use	1157.7
mobility	5.2
vaccine coverage	5104.8

E-Values for cumulative death rate regressions

**Factor**	**E-value**
Non-Hispanic White	1.7
Non-Hispanic Black	1.9
Non-Hispanic Asian	2.5
Non-Hispanic American Indian	1.6
Hispanic	2.1
percent living below the poverty line	3.1
income inequality	2.1
health access and quality	3.0
health spending per capita (price adjusted)	1.7
public health spending per capita (price adjusted)	1.3
public health employees per capita	1.3
hospital beds per capita	1.6
ICU beds per capita	2.2
physicians per capita	1.7
healthcare workers per capita	1.8

percent without health insurance	2.5
Republican governor	2.1
percent voted for Republican Pres candidate	2.5
interpersonal trust	2.3
trust in science	1.7
trust in government	1.1
paid family/sick leave	1.4
average years of education	2.5
citizen party affiliation	2.2
govt party affiliation	2.5
state house polarization	1.1
state senate polarization	1.2
affective polarization	1.8
economic connectedness	3.0
social clustering	1.6
volunteering rate	1.8
mask mandate	1.1
stay at home order	8.2
restaurant closure	4.7
bar closure	2.3
gathering restriction	1.6
primary school closure	4.4
higher education closure	1.2
gym/pool closure	4.5
vaccine mandates (schools)	4.1
vaccine mandates (state)	6.3
mandate propensity	2.1
mask use	4.5
mobility	1.3
vaccine coverage	11174.8

Section 6: Supplementary Results

6.1 Factors associated with age-adjusted cumulative death rates and cumulative infection rates

The below table shows relative reductions or increases in cumulative age-adjusted deaths or infections per capita that were associated with race and ethnicity (proportions of state population), pre-COVID-19 state characteristics, COVID-19 policy responses, and COVID-19 behavioural responses. The uncertainty interval (UI) around the estimate accounts for uncertainty in death or infection data. The cumulative death rate models controlled for comorbidity using the first principal component of a PCA of asthma, cancer, chronic obstructive pulmonary disease, cardiovascular disease, diabetes, BMI, and smoking prevalence. The models assessing COVID-19 policy responses (other than mandate propensity) also include an additional control variable that was the first component of all the other policy responses. The reported associations for the policy response should be interpreted as additional to the association tied to the mandate propensity variable. The values shown in this table correspond with Figure 3 in the main text.

Factors associated with age-adjusted cumulative death rates and cumulative infection rates

Dependent Variable	Factor	Category	Analysis Period	Relative Change	Lower UI	Upper UI	p-value	Relative change expressed with respect to change of
Deaths	Hispanic	Race/Ethnicity	01 January, 2020 - 31 July, 2022	11.38	2.14	21.65	0.008	0% Hispanic vs 100% Hispanic
Deaths	Non-Hispanic Asian	Race/Ethnicity	01 January, 2020 - 31 July, 2022	-14.27	-21.24	-6.67	>0.001	0% Asian vs 100% Asian
Deaths	Non-Hispanic Black	Race/Ethnicity	01 January, 2020 - 31 July, 2022	9.81	0.43	20.21	0.021	0% Black vs 100% Black
Deaths	Non-Hispanic White	Race/Ethnicity	01 January, 2020 - 31 July, 2022	-6.37	-14.17	2.11	0.070	0% White vs 100% White
Deaths	Non-Hispanic American Indian	Race/Ethnicity	01 January, 2020 - 31 July, 2022	5.36	-2.99	14.50	0.107	0% American Indian vs 100% American Indian
Deaths	Percent living below the poverty line	Pre-COVID-19 characteristic	01 January, 2020 - 31 July, 2022	23.42	14.84	32.77	>0.001	1 SD increase from the national mean
Deaths	Income inequality	Pre-COVID-19 characteristic	01 January, 2020 - 31 July, 2022	11.59	3.13	21.30	0.004	1 SD increase from the national mean
Deaths	Average years of education	Pre-COVID-19 characteristic	01 January, 2020 - 31 July, 2022	-14.25	-20.83	-6.99	>0.001	1 SD increase from the national mean
Deaths	Paid family/sick leave	Pre-COVID-19 characteristic	01 January, 2020 - 31 July, 2022	-2.41	-12.89	9.00	0.329	Reference category: no leave
Deaths	Republican governor	Pre-COVID-19 characteristic	01 January, 2020 - 31 July, 2022	11.25	-6.28	31.97	0.114	Reference category: Democrat

Deaths	Percent voted for Republican Pres candidate	Pre-COVID-19 characteristic	01 January, 2020 - 31 July, 2022	16.83	7.08	27.48	>0.001	1 SD increase from the national mean
Deaths	Health access and quality	Pre-COVID-19 characteristic	01 January, 2020 - 31 July, 2022	-18.62	-24.51	-12.27	>0.001	1 SD increase from the national mean
Deaths	Health spending per capita (price adjusted)	Pre-COVID-19 characteristic	01 January, 2020 - 31 July, 2022	-5.96	-13.71	2.66	0.085	1 SD increase from the national mean
Deaths	Healthcare workers per capita	Pre-COVID-19 characteristic	01 January, 2020 - 31 July, 2022	-7.30	-14.62	1.10	0.041	1 SD increase from the national mean
Deaths	Public health spending per capita (price adjusted)	Pre-COVID-19 characteristic	01 January, 2020 - 31 July, 2022	2.41	-6.24	12.10	0.300	1 SD increase from the national mean
Deaths	Public health employees per capita	Pre-COVID-19 characteristic	01 January, 2020 - 31 July, 2022	1.53	-6.56	10.44	0.364	1 SD increase from the national mean
Deaths	Percent without health insurance	Pre-COVID-19 characteristic	01 January, 2020 - 31 July, 2022	16.64	8.39	25.84	>0.001	1 SD increase from the national mean
Deaths	Interpersonal trust	Pre-COVID-19 characteristic	01 January, 2020 - 31 July, 2022	-12.88	-20.44	-4.45	0.002	1 SD increase from the national mean
Deaths	Trust in government	Pre-COVID-19 characteristic	01 January, 2020 - 31 July, 2022	-0.43	-8.68	8.52	0.459	1 SD increase from the national mean
Deaths	Trust in science	Pre-COVID-19 characteristic	01 January, 2020 - 31 July, 2022	-6.51	-14.81	2.40	0.072	1 SD increase from the national mean
Deaths	Mandate propensity	COVID-19 policy response	01 April, 2020 - 01 June, 2021	23.59	-9.53	70.70	0.100	Mandates never used vs mandate usage similar to that of Washington state
Deaths	Bar closure	COVID-19 policy response	01 April, 2020 - 01 June, 2021	27.88	-35.08	159.83	0.243	Mandate never used vs mandate in effect for 100% of analysis period
Deaths	Restaurant closure	COVID-19 policy response	01 April, 2020 - 01 June, 2021	-47.06	-90.19	192.98	0.233	Mandate never used vs mandate in effect for 100% of analysis period
Deaths	Gathering restriction	COVID-19 policy response	01 April, 2020 - 01 June, 2021	8.96	-36.97	89.40	0.382	Mandate never used vs mandate in effect for 100% of analysis period

Deaths	Primary school closure	COVID-19 policy response	01 April, 2020 - 01 June, 2021	79.68	-20.83	307.42	0.081	Mandate never used vs mandate in effect for 100% of analysis period
Deaths	Higher education closure	COVID-19 policy response	01 April, 2020 - 01 June, 2021	1.78	-58.76	145.63	0.481	Mandate never used vs mandate in effect for 100% of analysis period
Deaths	Gym/pool closure	COVID-19 policy response	01 April, 2020 - 01 June, 2021	-44.89	-88.90	187.30	0.236	Mandate never used vs mandate in effect for 100% of analysis period
Deaths	Mask mandate	COVID-19 policy response	01 April, 2020 - 01 June, 2021	0.54	-39.96	69.02	0.492	Mandate never used vs mandate in effect for 100% of analysis period
Deaths	Stay at home order	COVID-19 policy response	01 April, 2020 - 01 June, 2021	-62.04	-95.16	189.81	0.181	Mandate never used vs mandate in effect for 100% of analysis period
Deaths	Vaccine mandates (schools)	COVID-19 policy response	15 March, 2021 - 31 July, 2022	-29.30	-47.02	-5.85	0.009	Reference category: no history of mandate
Deaths	Vaccine mandates (state)	COVID-19 policy response	15 March, 2021 - 31 July, 2022	-39.22	-54.17	-18.62	>0.001	Reference category: no history of mandate
Deaths	Mask use	COVID-19 behavioral response	01 April, 2020 - 31 July, 2022	-34.09	-81.10	129.46	0.261	0% to 100% of the population wears a mask every time they leave the house
Deaths	Mobility	COVID-19 behavioral response	01 April, 2020 - 31 July, 2022	-1.76	-18.50	18.31	0.424	1 SD increase from the national mean change from pre-pandemic baseline
Deaths	Vaccine coverage	COVID-19 behavioral response	15 March, 2021 - 31 July, 2022	-97.42	-99.27	-90.97	>0.001	0% of population vaccinated vs 100% vaccinated for entire analysis period
Infections	Hispanic	Race/Ethnicity	01 January, 2020 - 15 December, 2021	1.99	-8.65	13.73	0.358	0% Hispanic vs 100% Hispanic
Infections	Non-Hispanic Asian	Race/Ethnicity	01 January, 2020 - 15 December, 2021	-18.20	-24.14	-11.86	>0.001	0% Asian vs 100% Asian
Infections	Non-Hispanic Black	Race/Ethnicity	01 January, 2020 - 15 December, 2021	5.14	-3.29	14.42	0.120	0% Black vs 100% Black
Infections	Non-Hispanic White	Race/Ethnicity	01 January, 2020 - 15 December, 2021	5.60	-4.50	16.81	0.141	0% White vs 100% White

Infections	Non-Hispanic American Indian	Race/Ethnicity	01 January, 2020 - 15 December, 2021	3.80	-4.80	13.14	0.195	0% American Indian vs 100% American Indian
Infections	Percent living below the poverty line	Pre-COVID-19 characteristic	01 January, 2020 - 15 December, 2021	10.96	1.93	20.93	0.007	1 SD increase from the national mean
Infections	Income inequality	Pre-COVID-19 characteristic	01 January, 2020 - 15 December, 2021	2.39	-5.98	11.58	0.295	1 SD increase from the national mean
Infections	Average years of education	Pre-COVID-19 characteristic	01 January, 2020 - 15 December, 2021	-11.29	-18.98	-3.02	0.004	1 SD increase from the national mean
Infections	Paid family/sick leave	Pre-COVID-19 characteristic	01 January, 2020 - 15 December, 2021	-13.43	-22.83	-3.02	0.008	Reference category: no leave
Infections	Republican governor	Pre-COVID-19 characteristic	01 January, 2020 - 15 December, 2021	17.48	-0.93	39.79	0.067	Reference category: Democrat
Infections	Percent voted for Republican Pres candidate	Pre-COVID-19 characteristic	01 January, 2020 - 15 December, 2021	27.08	17.33	37.72	>0.001	1 SD increase from the national mean
Infections	Health access and quality	Pre-COVID-19 characteristic	01 January, 2020 - 15 December, 2021	-12.51	-19.74	-4.68	>0.001	1 SD increase from the national mean
Infections	Public health spending per capita (price adjusted)	Pre-COVID-19 characteristic	01 January, 2020 - 15 December, 2021	-5.95	-13.82	2.56	0.082	1 SD increase from the national mean
Infections	Public health employees per capita	Pre-COVID-19 characteristic	01 January, 2020 - 15 December, 2021	-0.74	-8.84	8.33	0.427	1 SD increase from the national mean
Infections	Interpersonal trust	Pre-COVID-19 characteristic	01 January, 2020 - 15 December, 2021	-10.51	-17.66	-2.78	0.005	1 SD increase from the national mean
Infections	Trust in government	Pre-COVID-19 characteristic	01 January, 2020 - 15 December, 2021	-1.50	-9.90	7.72	0.375	1 SD increase from the national mean
Infections	Trust in science	Pre-COVID-19 characteristic	01 January, 2020 - 15 December, 2021	-8.00	-15.14	-0.49	0.019	1 SD increase from the national mean
Infections	Mandate propensity	COVID-19 policy response	01 April, 2020 - 01 June, 2021	-36.27	-52.66	-14.55	0.002	Mandates never used vs mandate usage similar to that of Washington state
Infections	Bar closure	COVID-19 policy response	01 April, 2020 - 01 June, 2021	-4.77	-49.19	82.79	0.437	Mandate never used vs mandate in effect for 100% of analysis period

Infections	Restaurant closure	COVID-19 policy response	01 April, 2020 - 01 June, 2021	-17.57	-83.14	303.78	0.402	Mandate never used vs mandate in effect for 100% of analysis period
Infections	Gathering restriction	COVID-19 policy response	01 April, 2020 - 01 June, 2021	-26.06	-55.58	21.06	0.119	Mandate never used vs mandate in effect for 100% of analysis period
Infections	Primary school closure	COVID-19 policy response	01 April, 2020 - 01 June, 2021	-29.47	-67.62	50.05	0.190	Mandate never used vs mandate in effect for 100% of analysis period
Infections	Higher education closure	COVID-19 policy response	01 April, 2020 - 01 June, 2021	32.39	-40.08	204.60	0.248	Mandate never used vs mandate in effect for 100% of analysis period
Infections	Gym/pool closure	COVID-19 policy response	01 April, 2020 - 01 June, 2021	-11.35	-80.27	318.34	0.435	Mandate never used vs mandate in effect for 100% of analysis period
Infections	Mask mandate	COVID-19 policy response	01 April, 2020 - 01 June, 2021	-24.63	-54.11	21.81	0.131	Mandate never used vs mandate in effect for 100% of analysis period
Infections	Stay at home order	COVID-19 policy response	01 April, 2020 - 01 June, 2021	-67.32	-95.37	117.72	0.127	Mandate never used vs mandate in effect for 100% of analysis period
Infections	Vaccine mandates (schools)	COVID-19 policy response	15 March, 2021 - 15 December, 2021	-18.65	-35.18	1.88	0.064	Reference category: no history of mandate
Infections	Vaccine mandates (state)	COVID-19 policy response	15 March, 2021 - 15 December, 2021	-22.19	-38.24	-2.18	0.016	Reference category: no history of mandate
Infections	Mask use	COVID-19 behavioral response	01 April, 2020 - 15 December, 2021	-94.60	-98.46	-81.99	>0.001	0% to 100% of the population wears a mask every time they leave the house
Infections	Mobility	COVID-19 behavioral response	01 April, 2020 - 15 December, 2021	62.13	29.61	102.54	>0.001	1 SD increase from the national mean change from pre-pandemic baseline
Infections	Vaccine coverage	COVID-19 behavioral response	15 March, 2021 - 15 December, 2021	-93.11	-97.74	-78.57	>0.001	0% of population vaccinated vs 100% vaccinated for entire analysis period

6.2 Factors associated with reduction in standardised GDP, employment rate, and mathematics and reading test scores

The table below shows estimated associations of COVID-19 policy and behavioural responses with state GDP (sector-standardised and defined as the ratio of expected to actual GDP) and employment per capita (sector-standardised and defined as the ratio of expected to actual employment). The GDP and employment models control for education, proportion of the population older than 65 years, proportion of the population younger than 20 years, mean weekly state unemployment benefits, and mean state unemployment benefit duration. The subsequent table shows estimated associations of COVID-19 policy and behavioural responses with changes in fourth-grade mathematics and reading test scores. The values shown in these two tables correspond with Figure 7 in the main text.

Factors associated with reduction in standardised GDP and employment rate

Dependent Variable	Factor	Category	Analysis Period	Relative Change	Lower UI	Upper UI	Relative change expressed with respect to change of
GDP	Mask mandate	COVID-19 policy response	01 April, 2020 - 01 June, 2021	0.63	-2.53	3.89	Mandate never used vs mandate in effect for 100% of analysis period
GDP	Stay at home order	COVID-19 policy response	01 April, 2020 - 01 June, 2021	1.03	-9.94	13.33	Mandate never used vs mandate in effect for 100% of analysis period
GDP	Restaurant closure	COVID-19 policy response	01 April, 2020 - 01 June, 2021	-4.29	-12.47	4.66	Mandate never used vs mandate in effect for 100% of analysis period
GDP	Bar closure	COVID-19 policy response	01 April, 2020 - 01 June, 2021	-1.13	-4.62	2.49	Mandate never used vs mandate in effect for 100% of analysis period
GDP	Gathering restriction	COVID-19 policy response	01 April, 2020 - 01 June, 2021	-0.77	-3.62	2.17	Mandate never used vs mandate in effect for 100% of analysis period
GDP	Primary school closure	COVID-19 policy response	01 April, 2020 - 01 June, 2021	2.43	-2.02	7.10	Mandate never used vs mandate in effect for 100% of analysis period

GDP	Higher education closure	COVID-19 policy response	01 April, 2020 - 01 June, 2021	2.43	-2.39	7.48	Mandate never used vs mandate in effect for 100% of analysis period
GDP	Gym/pool closure	COVID-19 policy response	01 April, 2020 - 01 June, 2021	2.16	-6.29	11.36	Mandate never used vs mandate in effect for 100% of analysis period
GDP	Vaccine mandates (schools)	COVID-19 policy response	15 March, 2021 - 30 June, 2022	-2.48	-5.12	0.22	Reference category: no history of mandate
GDP	Vaccine mandates (state)	COVID-19 policy response	01 April, 2020 - 01 June, 2021	-1.85	-4.49	0.88	Reference category: no history of mandate
GDP	Mandate propensity	COVID-19 policy response	01 April, 2020 - 01 June, 2021	0.36	-1.67	2.43	Mandates never used vs mandate usage similar to that of Washington state
GDP	Federal UI benefit duration	COVID-19 policy response	01 April, 2020 - 01 June, 2021	-3.39	-10.02	3.73	1% increase in coverage duration relative to the maximum duration
GDP	Federal UI benefit amount	COVID-19 policy response	01 April, 2020 - 01 June, 2021	-0.01	-0.03	0.01	One dollar increase in the mean weekly benefit amount
GDP	Mask use	COVID-19 behavioral response	01 April, 2020 - 01 June, 2021	-7.47	-18.87	5.54	0% to 100% of the population wears a mask every time they leave the house
GDP	Vaccine coverage	COVID-19 behavioral response	01 April, 2020 - 01 June, 2021	7.65	-14.96	36.27	0% of population vaccinated vs 100% vaccinated for entire analysis period
GDP	Mobility	COVID-19 behavioral response	01 April, 2020 - 30 June, 2022	0.07	-1.92	2.10	1 SD increase from the national mean change from pre-pandemic baseline

Employment	Mask mandate	COVID-19 policy response	01 April, 2020 - 01 June, 2021	-0.53	-2.98	1.98	Mandate never used vs mandate in effect for 100% of analysis period
Employment	Stay at home order	COVID-19 policy response	01 April, 2020 - 01 June, 2021	-5.67	-13.63	3.03	Mandate never used vs mandate in effect for 100% of analysis period
Employment	Restaurant closure	COVID-19 policy response	01 April, 2020 - 01 June, 2021	-6.74	-12.83	-0.23	Mandate never used vs mandate in effect for 100% of analysis period
Employment	Bar closure	COVID-19 policy response	01 April, 2020 - 01 June, 2021	0.99	-1.80	3.86	Mandate never used vs mandate in effect for 100% of analysis period
Employment	Gathering restriction	COVID-19 policy response	01 April, 2020 - 01 June, 2021	-0.52	-2.78	1.78	Mandate never used vs mandate in effect for 100% of analysis period
Employment	Primary school closure	COVID-19 policy response	01 April, 2020 - 01 June, 2021	1.20	-2.27	4.80	Mandate never used vs mandate in effect for 100% of analysis period
Employment	Higher education closure	COVID-19 policy response	01 April, 2020 - 01 June, 2021	-0.34	-4.06	3.52	Mandate never used vs mandate in effect for 100% of analysis period
Employment	Gym/pool closure	COVID-19 policy response	01 April, 2020 - 01 June, 2021	-4.09	-10.26	2.50	Mandate never used vs mandate in effect for 100% of analysis period
Employment	Vaccine mandates (schools)	COVID-19 policy response	15 March, 2021 - 31 July, 2022	-1.42	-2.94	0.11	Reference category: no history of mandate
Employment	Vaccine mandates (state)	COVID-19 policy response	15 March, 2021 - 31 July, 2022	-0.29	-1.85	1.29	Reference category: no history of mandate

Employment	Mandate propensity	COVID-19 policy response	01 April, 2020 - 01 June, 2021	-0.59	-2.16	1.01	Mandates never used vs mandate usage similar to that of Washington state
Employment	Federal UI benefit duration	COVID-19 policy response	01 April, 2020 - 31 July, 2022	-4.13	-8.39	0.32	1% increase in coverage duration relative to the maximum duration
Employment	Federal UI benefit amount	COVID-19 policy response	01 April, 2020 - 31 July, 2022	-0.01	-0.02	0.00	One dollar increase in the mean weekly benefit amount
Employment	Mask use	COVID-19 behavioral response	01 April, 2020 - 31 July, 2022	-11.04	-18.07	-3.40	0% to 100% of the population wears a mask every time they leave the house
Employment	Vaccine coverage	COVID-19 behavioral response	15 March, 2021 - 15 December, 2021	-2.08	-14.65	12.33	0% of population vaccinated vs 100% vaccinated for entire analysis period
Employment	Mobility	COVID-19 behavioral response	01 April, 2020 - 31 July, 2022	0.69	-0.58	1.99	1 SD increase from the national mean change from pre-pandemic baseline

Factors associated with reduction in standardized test scores for 4th grade mathematics and reading

Dependent Variable	Factor	Category	Analysis Period	Absolute Change	Lower UI	Upper UI	Marginal change expressed with respect to
Math score	Mask mandate	COVID-19 policy response	01 April, 2020 - 15 February, 2022	-3.85	-6.92	-0.78	Mandate never used vs mandate in effect for 100% of analysis period
Math score	Stay at home order	COVID-19 policy response	01 April, 2020 - 15 February, 2022	-6.93	-23.62	9.77	Mandate never used vs mandate in effect for 100% of analysis period
Math score	Gathering restriction	COVID-19 policy response	01 April, 2020 - 15 February, 2022	-2.80	-6.40	0.80	Mandate never used vs mandate in effect for 100% of analysis period

Math score	Primary school closure	COVID-19 policy response	01 April, 2020 - 15 February, 2022	-5.36	-10.98	0.25	Mandate never used vs mandate in effect for 100% of analysis period
Math score	Higher education closure	COVID-19 policy response	01 April, 2020 - 15 February, 2022	-4.09	-11.72	3.53	Mandate never used vs mandate in effect for 100% of analysis period
Math score	Vaccine mandates (schools)	COVID-19 policy response	01 January, 2020 - 15 February, 2022	-2.26	-4.13	-0.39	Reference category: no history of mandate
Math score	Vaccine mandates (state)	COVID-19 policy response	01 January, 2020 - 15 February, 2022	-3.04	-4.48	-1.60	Reference category: no history of mandate
Math score	Mandate propensity	COVID-19 policy response	01 January, 2020 - 15 February, 2022	-3.68	-6.56	-0.79	Mandates never used vs mandate usage similar to that of Washington state
Math score	Mask use	COVID-19 behavioral response	01 April, 2020 - 15 February, 2022	-12.97	-20.98	-4.96	0% to 100% of the population wears a mask every time they leave the house
Math score	Vaccine coverage	COVID-19 behavioral response	01 April, 2020 - 15 February, 2022	-31.29	-51.82	-10.77	0% of population vaccinated vs 100% vaccinated for entire analysis period
Math score	Mobility	COVID-19 behavioral response	01 April, 2020 - 15 February, 2022	2.18	0.94	3.43	1 SD increase from the national mean change from pre-pandemic baseline
Reading score	Mask mandate	COVID-19 policy response	01 April, 2020 - 15 February, 2022	-1.56	-4.69	1.57	Mandate never used vs mandate in effect for 100% of analysis period
Reading score	Stay at home order	COVID-19 policy response	01 April, 2020 - 15 February, 2022	0.25	-16.08	16.59	Mandate never used vs mandate in effect for 100% of analysis period
Reading score	Gathering restriction	COVID-19 policy response	01 April, 2020 - 15 February, 2022	-1.55	-5.11	2.00	Mandate never used vs mandate in effect for 100% of analysis period
Reading score	Primary school closure	COVID-19 policy response	01 April, 2020 - 15 February, 2022	-1.66	-7.29	3.97	Mandate never used vs mandate in effect for 100% of analysis period

Reading score	Higher education closure	COVID-19 policy response	01 April, 2020 - 15 February, 2022	-2.07	-9.54	5.40	Mandate never used vs mandate in effect for 100% of analysis period
Reading score	Vaccine mandates (schools)	COVID-19 policy response	01 January, 2020 - 15 February, 2022	-0.75	-2.65	1.16	Reference category: no history of mandate
Reading score	Vaccine mandates (state)	COVID-19 policy response	01 January, 2020 - 15 February, 2022	-1.79	-3.34	-0.24	Reference category: no history of mandate
Reading score	Mandate propensity	COVID-19 policy response	01 January, 2020 - 15 February, 2022	-1.60	-4.54	1.35	Mandates never used vs mandate usage similar to that of Washington state
Reading score	Mask use	COVID-19 behavioral response	01 April, 2020 - 15 February, 2022	-2.79	-11.30	5.72	0% to 100% of the population wears a mask every time they leave the house
Reading score	Vaccine coverage	COVID-19 behavioral response	01 April, 2020 - 15 February, 2022	-22.20	-42.98	-1.41	0% of population vaccinated vs 100% vaccinated for entire analysis period
Reading score	Mobility	COVID-19 behavioral response	01 April, 2020 - 15 February, 2022	0.72	-0.61	2.05	1 SD increase from the national mean change from pre-pandemic baseline
